# C6orf203 is an RNA-binding protein involved in mitochondrial protein synthesis

**DOI:** 10.1093/nar/gkz684

**Published:** 2019-08-09

**Authors:** Shreekara Gopalakrishna, Sarah F Pearce, Adam M Dinan, Florian A Rosenberger, Miriam Cipullo, Henrik Spåhr, Anas Khawaja, Camilla Maffezzini, Christoph Freyer, Anna Wredenberg, Ilian Atanassov, Andrew E Firth, Joanna Rorbach

**Affiliations:** Department of Medical Biochemistry and Biophysics, Division of Molecular Metabolism, Karolinska Institutet, Solnavägen 9, 171 77 Stockholm, Sweden; Max Planck Institute Biology of Ageing - Karolinska Institutet Laboratory, Karolinska Institutet, 171 77 Stockholm, Sweden; Department of Medical Biochemistry and Biophysics, Division of Molecular Metabolism, Karolinska Institutet, Solnavägen 9, 171 77 Stockholm, Sweden; Max Planck Institute Biology of Ageing - Karolinska Institutet Laboratory, Karolinska Institutet, 171 77 Stockholm, Sweden; Department of Pathology, University of Cambridge, CB2 0QQ Cambridge, UK; Max Planck Institute Biology of Ageing - Karolinska Institutet Laboratory, Karolinska Institutet, 171 77 Stockholm, Sweden; Department of Molecular Medicine and Surgery, Karolinska Institutet, 171 76 Stockholm, Sweden; Department of Medical Biochemistry and Biophysics, Division of Molecular Metabolism, Karolinska Institutet, Solnavägen 9, 171 77 Stockholm, Sweden; Max Planck Institute Biology of Ageing - Karolinska Institutet Laboratory, Karolinska Institutet, 171 77 Stockholm, Sweden; Department of Medical Biochemistry and Biophysics, Division of Molecular Metabolism, Karolinska Institutet, Solnavägen 9, 171 77 Stockholm, Sweden; Max Planck Institute Biology of Ageing - Karolinska Institutet Laboratory, Karolinska Institutet, 171 77 Stockholm, Sweden; Department of Medical Biochemistry and Biophysics, Division of Molecular Metabolism, Karolinska Institutet, Solnavägen 9, 171 77 Stockholm, Sweden; Max Planck Institute Biology of Ageing - Karolinska Institutet Laboratory, Karolinska Institutet, 171 77 Stockholm, Sweden; Department of Medical Biochemistry and Biophysics, Division of Molecular Metabolism, Karolinska Institutet, Solnavägen 9, 171 77 Stockholm, Sweden; Max Planck Institute Biology of Ageing - Karolinska Institutet Laboratory, Karolinska Institutet, 171 77 Stockholm, Sweden; Department of Medical Biochemistry and Biophysics, Division of Molecular Metabolism, Karolinska Institutet, Solnavägen 9, 171 77 Stockholm, Sweden; Max Planck Institute Biology of Ageing - Karolinska Institutet Laboratory, Karolinska Institutet, 171 77 Stockholm, Sweden; Department of Medical Biochemistry and Biophysics, Division of Molecular Metabolism, Karolinska Institutet, Solnavägen 9, 171 77 Stockholm, Sweden; Max Planck Institute Biology of Ageing - Karolinska Institutet Laboratory, Karolinska Institutet, 171 77 Stockholm, Sweden; Proteomics Core Facility, Max-Planck-Institute for Biology of Ageing, Joseph-Stelzmann-Str. 9b, 50931 Cologne, Germany; Department of Pathology, University of Cambridge, CB2 0QQ Cambridge, UK; Department of Medical Biochemistry and Biophysics, Division of Molecular Metabolism, Karolinska Institutet, Solnavägen 9, 171 77 Stockholm, Sweden; Max Planck Institute Biology of Ageing - Karolinska Institutet Laboratory, Karolinska Institutet, 171 77 Stockholm, Sweden

## Abstract

In all biological systems, RNAs are associated with RNA-binding proteins (RBPs), forming complexes that control gene regulatory mechanisms, from RNA synthesis to decay. In mammalian mitochondria, post-transcriptional regulation of gene expression is conducted by mitochondrial RBPs (mt-RBPs) at various stages of mt-RNA metabolism, including polycistronic transcript production, its processing into individual transcripts, mt-RNA modifications, stability, translation and degradation. To date, only a handful of mt-RBPs have been characterized. Here, we describe a putative human mitochondrial protein, C6orf203, that contains an S4-like domain—an evolutionarily conserved RNA-binding domain previously identified in proteins involved in translation. Our data show C6orf203 to bind highly structured RNA *in vitro* and associate with the mitoribosomal large subunit in HEK293T cells. Knockout of *C6orf203* leads to a decrease in mitochondrial translation and consequent OXPHOS deficiency, without affecting mitochondrial RNA levels. Although mitoribosome stability is not affected in *C6orf203*-depleted cells, mitoribosome profiling analysis revealed a global disruption of the association of mt-mRNAs with the mitoribosome, suggesting that C6orf203 may be required for the proper maturation and functioning of the mitoribosome. We therefore propose C6orf203 to be a novel RNA-binding protein involved in mitochondrial translation, expanding the repertoire of factors engaged in this process.

## INTRODUCTION

Ribonucleic acid (RNA)-binding proteins (RBPs) play a central role in mediating the regulation of gene expression in all organisms. They associate with nascent transcripts and subsequently regulate all steps of mRNA life from processing, localization, translation to turnover. Multiple RBPs also associate with noncoding RNA (ncRNA), affecting their structures and catalytic activities. The number of novel RBPs is rapidly growing due to the implementation of highly sensitive identification tools (1). In recent years, RNA interactome capture and its modifications have identified over 1000 eukaryotic RBPs, many of which had no prior RNA-related annotation (2,3). Importantly, defects in many of these factors have been implicated in a broad spectrum of human pathologies, prominently neurological, sensory, and muscular disorders and cancers (4–6). A deeper insight into the role of RBPs in genetic disease requires the in-depth characterization of their mechanism of action *in vivo* and dynamic mapping of their target interactions.

In human mitochondria, post-transcriptional gene expression is orchestrated by numerous RBPs that are produced in the cytosol and imported into the organelle. The mitochondrial genome (mtDNA) encodes 13 proteins, all of which are essential components of the oxidative phosphorylation (OXPHOS) system. In addition to mt-mRNAs, all RNA elements required for *in organellar* translation, two ribosomal RNAs (mt-rRNAs: 12S and 16S) incorporated into the mitochondrial ribosome (mitoribosome), and a complete set of transfer RNAs (22 mt-tRNAs) are also encoded by mtDNA. From the moment the nascent polycistronic transcripts are produced, mtRBPs are involved in their processing and maturation: long polycistronic transcripts undergo endonucleolytic processing by RNase P and ELAC2 that releases individual transcripts. Most of the mRNAs are polyadenylated and stabilized by interaction with the LRPPRC–SLIRP complex, mt-rRNAs are subjected to methylation and pseudouridylation, and mt-tRNAs undergo several types of nucleotide modifications, CCA addition and aminoacylation (7). Many of these processes take place in mitochondrial RNA granules, dynamic structures that also serve as hubs for early mitoribosome assembly (8).

Human mitoribosomes are complex machineries consisting of 82 protein and 3 RNA components (2 rRNAs and mt-tRNA^Val^) (9,10). Their assembly proceeds in a highly organized manner, requiring the action of numerous factors that help mt-rRNA folding, coordinate sequential arrival of integral ribosomal proteins and allow association of the mitoribosome with the mitochondrial inner membrane. Several GTPases, RNA helicases and chaperone‐like factors have been implicated in mitoribosome biogenesis, although our understanding of this process is far from complete (11,12). The high complexity of the human mitoribosome and numerous differences between the mitochondrial and bacterial translation systems suggest the involvement of as-yet-unknown mitochondria-specific factors, with no bacterial descendants.

This missing information led us in search of putative mitochondrial RBPs involved in mitochondrial gene expression and mitoribosome maturation. *In silico* analysis of the proteins that contain RNA-binding domains, together with our systematic SILAC-based mass spectrometry detection of the components of mitochondrial translation machinery (unpublished), directed our focus to C6orf203 (Chromosome 6 open reading frame 203), a putative mitochondrial protein listed in the MitoCarta inventory of mammalian mitochondrial proteins (13). C6orf203 is predicted to contain an S4-like domain, an RNA-binding domain found in several translation-associated RBPs, including ribosomal protein S4 (14). Here, we show that C6orf203 binds highly structured RNA *in vitro* and interacts with the mitochondrial ribosomal large subunit (mt-LSU) in human cells. Loss of *C6orf203* leads to diminished mitochondrial translation and consequently, to respiratory incompetence. We propose that C6orf203 is required for the maturation/modulation of activity of the mitoribosomal large subunit and, therefore, efficient mitochondrial translation.

## MATERIALS AND METHODS

For a comprehensive list of antibodies and oligonucleotides used in this study, please refer to accompanying Supplementary Tables S1–S5.

### Cell maintenance and generation of stable cell lines

To generate stable mammalian cell lines that possess a doxycycline-inducible expression of the C-terminal FLAG tagged C6orf203 (C6orf203::FLAG) in a dose-dependent manner, Flp-In T-Rex human embryonic kidney 293T (HEK293T, Invitrogen) cell line was used. HEK293T cells were cultured in DMEM (Dulbecco’s modified Eagle medium) supplemented with 10% (v/v) tetracycline-free fetal bovine serum (FBS), 2 mM GlutaMax (Gibco), 1× Penicillin/Streptomycin (Gibco), 50 μg/ml uridine, 100 μg/ml Zeocin (Invitrogen) and 10 μg/ml blasticidin (Gibco) at 37°C under 5% CO_2_ humidified atmosphere.

Twenty-four hours prior to transfection, cells were split to 100-mm plates in culture medium lacking selective antibiotics and grown to 80–90% confluence. Transfection of pcDNA5/FRT/TO-*C6orf203::FLAG* construct and pOG44 was performed using Lipofectamine 3000 (Invitrogen) according to the manufacturer’s instructions. Forty-eight hours after transfection, the selective antibiotics hygromycin (100 μg/ml, Invitrogen) and blasticidin (10 μg/ml) were added, and selective medium was replaced every 3–4 days.

For immunocytochemistry, human 143B osteosarcoma (HOS) cells were cultured in DMEM supplemented with 10% (v/v) FBS, 2 mM GlutaMax and 1× Penicillin/Streptomycin at 37°C under 5% CO_2_ humidified atmosphere. Cells were transfected with pcDNA5/FRT/TO-*C6orf203::FLAG* using Lipofectamine 3000 according to manufacturer’s instructions.

The generation of the HEK293T knockout cell line for *C6orf203* was performed using CRISPR/Cas9 system as described in (15). Briefly, guide RNAs (gRNAs) designed to generate an out-of-frame deletion in exon 1 were cloned into pSpCas9(BB)-2A-Puro (pX459) V2.0 vector. *C6orf203* targeting pX459 vectors were transfected to cells using Lipofectamine 3000 according to the manufacturer’s instructions. After 48 h of puromycin (1.5 μg/ml) selection, cells were serially diluted to plate single cells in each well of a 96-well plate. Resultant clones were screened by PCR and Western blotting. Loss of C6orf203 in selected clones was confirmed by Sanger sequencing.

For growth measurements, 50 000 cells were seeded in six-well plates in glucose-free DMEM containing 10 mM Galactose, 10% (v/v) FBS, 2 mM GlutaMax, 1× Penicillin/Streptomycin and 1× Sodium Pyruvate (Gibco). Cell number was counted every 48 h up to 6 days with EVE™ Automated Cell Counter (NanoEnTek) as per the manufacturer’s instructions.

### Immunodetection of proteins

For immunoblot analysis, cultured cells or isolated mitochondria were lysed and the protein concentration was determined by Pierce™ BCA Protein Assay Kit (Thermo Fisher Scientific). Equal amounts of proteins corresponding to total cell lysates or protein fractions were subjected to SDS-PAGE, wet transferred to PVDF membranes, blocked in 5% non-fat milk (Semper) in PBS for 1 h and incubated with specific primary antibodies in 5% non-fat milk in PBS for 1 h or overnight. The blots were further incubated with HRP-conjugated secondary antibodies in 5% non-fat milk in PBS for 1 h and visualized using ECL (BioRad). The list of antibodies used are listed in Supplementary Table S1.

For immunofluorescence studies, 143B cells were plated on coverslips (at ∼70% confluency), incubated with MitoTracker Red CMXRos for 15 min and fixed with 3% formaldehyde. Following membrane permeabilization (1% Triton in PBS for 5 min) and blocking (3% FBS in PBS, 1 h), cells were incubated with anti-FLAG monoclonal antibodies (1:200) for 2 h, washed with PBS and incubated with Alexa Fluor 488 conjugated anti-mouse antibodies for 1 h. Immunofluorescence images were then captured using a Zeiss LSM800 confocal microscope.

### Cell fractionation

Cell fractionation was performed as described in (16) without the sucrose gradient step.

### [^35^S]-Methionine *in vivo* mitochondrial translation assay

Cells on a six-well plate were washed twice for 5 min each in methionine/cysteine-free DMEM and incubated with methionine/cysteine-free DMEM supplemented with 10% dialyzed FBS, GlutaMax 100X (Gibco), sodium pyruvate 100× (Gibco) and 100 μg/ml emetine (Sigma-Aldrich) (an irreversible cytosolic translation inhibitor) for 20 min at 37°C. Cells were then incubated in the same media supplemented with 166.7 μCi/ml of [^35^S]-methionine (Perkin Elmer) for 30 min at 37°C to label the newly synthesized mitochondrially encoded proteins. After washing three times with PBS, cells were lysed and 30 μg aliquots were separated on 10–20% Tris-glycine (Invitrogen) SDS-PAGE gels. Gels were dried for 2 h at 65°C and exposed on a phosphoimager screen prior to visualization with Typhoon FLA 7000 phosphoimager.

### In-gel activity of mitochondrial respiratory complexes

Mitochondria isolated from the different cell types were resuspended in 1.5 M aminocaproic acid (ACNA), quantified by Qubit (for loading 50 μg), lysed with 4% w/v digitonin and separated by Blue-Native Polyacrylamide Gel Electrophoresis (BN-PAGE). Mitochondrial respiratory chain enzyme activities were determined in-gel with OXPHOS complex-specific substrate buffers as described in (17), with minor adaptation. About 2.5 mg/ml of iodonitrotetrazolium chloride was used instead of nitrotetrazolium blue in complex-I and complex-II substrate buffers.

### Measurement of mitochondrial respiration

To determine mitochondrial respiration in wild-type, C6orf203KO^1^ and C6orf203KO^1^ overexpressing C6orf203::FLAG cell lines, high-resolution respirometry with Oroboros Oxygraph-2K (Oroboros Instruments, Austria) was performed at 37°C. Basal mitochondrial oxygen consumption was measured in MIR05 medium, followed by injection of 4.05 μM of digitonin to permeabilize the cells. After adding 2.5 μM of oligomycin (Complex V inhibitor), maximal mitochondrial oxygen consumption was measured with 0.5 μM steps of carbonyl cyanide m-chlorophenyl hydrazone (CCCP) titration.

### Immunoprecipitation of C6orf203::FLAG

Mitochondria were isolated from HEK293T Flp-In cells overexpressing C6orf203::FLAG as described in (18) and lysed in 1× lysis buffer (Sigma-Aldrich) supplemented with 5 mM MgCl_2_ and 1× protease inhibitor cocktail (Roche). Anti-FLAG M2 Agarose Affinity gel beads (Sigma-Aldrich) were used for immunoprecipitation according to manufacturer’s instructions. The wash and elution buffers were supplemented with 5 mM MgCl_2_. Elution was performed with the 3× FLAG peptide (Sigma-Aldrich). The eluate was immediately stored at −80°C and used for immunoblotting or processed for mass spectrometry.

### Liquid chromatography (LC)–mass spectrometry (MS)/MS analysis

Mass spectrometric analysis of immunoprecipitated complexes was performed as described in Supplementary Data. A list of proteins detected by MS is presented in Supplementary Table S6.

### RNA isolation and northern blot analysis

TRIzol (Invitrogen) reagent was used to isolate total RNA from cells as per the manufacturer’s instructions. About 4 μg of total RNA was resolved in 1.2% agarose gels with 0.7% formaldehyde and GelRed® (Biotium) in 1× MOPS buffer. The RNA was transferred to a nylon membrane overnight by capillary-based transfer and used for probing rRNAs and mRNAs. For tRNA northern blots, RNA was resolved in TBE-Urea Gel (15%) in 1× TBE buffer followed by wet transfer at 30 V for 1 h in 0.5× TBE buffer. Membranes were then UV crosslinked and hybridized with probes corresponding to the mitochondrially encoded mRNAs or tRNAs. Washing the membranes after probing was performed with 1× SSC buffer (150 mM NaCl, 15 mM tri-sodium citrate (pH 7.0)) supplemented with 0.1% SDS. Probes for mRNA and tRNA were prepared by Prime-It II Random Primer Labeling Kit (Agilent technologies) with 50 μCi of CTP and Riboprobe® *in vitro* Transcription Systems (Promega) with 50 μCi of UTP respectively, according to manufacturer’s instructions. Oligonucleotides and primers used for the synthesis of tRNA and mRNA probes are listed in the Supplementary Tables S3 and S4, respectively. Membranes were then exposed on a phosphoimager screen and visualized using Typhoon FLA 7000 phosphoimager.

### qRT-PCR to determine RNA interactors of C6orf203

Mitochondria isolated from HEK293T Flp-In cells and HEK293T Flp-In cells overexpressing C6orf203::FLAG were lysed in 1× lysis buffer (Sigma-Aldrich) supplemented with 5 mM MgCl_2_ and 1× protease inhibitor cocktail (Roche). Protein content of mitolysates were quantified via BCA assay to load the same protein mass on Anti-FLAG M2 Agarose Affinity gel beads (Sigma-Aldrich) for immunoprecipitation. RNA from mitolysates (inputs) and FLAG-immunoprecipitated eluates were extracted by TRIzol (Invitrogen) according to manufacturer’s instructions. RNAs in the inputs were quantified and used for cDNA synthesis (reaction volume = 20 μl) with high-capacity cDNA reverse transcription kit (Applied Biosystems) and ProFlex PCR System (Applied Biosystems). RNA purity was assessed by the ratio of *A*_260_/*A*_280_ by NanoDrop ND-1000 UV-Vis Spectrophotometer (Thermo Fisher Scientific), to be 2.0. In addition, an equal volume of eluates was used for reverse transcription. qPCR was performed by QuantStudio 6 Flex Real-Time PCR System (Applied Biosystems) with the cDNA using TaqMan Universal PCR Master Mix (Applied Biosystems) and TaqMan probes with FAM (6-carboxyfluorescein) reporter (Applied Biosystems) (listed in Supplementary Table S5) with 5–10 ng of cDNA per 10 μl reaction mix. *C*_t_ values were obtained from the in-built software (QuantStudio Real-Time PCR software v1.1) associated with the qPCR machine and data analysis was performed using Microsoft Office Excel. The relative fold change of transcripts in the FLAG-primed immunoprecipitated eluates versus the non-primed eluates were calculated using the formula: ((2^*C*t^ of eluate – 2^*C*t^ of input) of non-FLAG primed IP) / ((2^*C*t^ of eluate – 2^*C*t^ of input) of FLAG primed IP). Figure 5C represents three biological replicates and the error bars represents the standard error of mean (SEM).

### Analysis of mitochondrial ribosomes on sucrose gradients

Mitochondria were isolated from cells, as described in (18), and lysed in 1× lysis buffer (Sigma-Aldrich) containing 5 mM MgCl_2_, 1× protease inhibitor cocktail (Roche) and RNase block (Agilent). Approximately 500 μg of protein was loaded onto linear sucrose gradients (10–30% (v/v)), prepared in the gradient buffer (20 mM Tris-HCl (pH 7.4), 100 mM NaCl, 20 mM MgCl_2_). The samples were centrifuged for 15 h at 79 000 × *g* at 4°C (Beckman Coulter; SW41 rotor). 21 fractions of 450 μl each were collected and the first 17 fractions were used for western blot analysis.

### Purification of C6orf203 and electrophoretic mobility shift assay (EMSA)

A codon-optimized (Genscript) DNA construct corresponding to the mature form of human C6orf203 (amino acids 85–240) was cloned into a Fh8-Pet24d vector. Human C6orf203 was expressed in Arctic express cells (Agilent) at 16°C for 48 h in Magic Media (Thermo Fisher Scientific) with an approximate yield of 5 mg/l, assessed by Bradford protein assay. After lysis, the proteins were purified over a His-Select Ni^2+^ resin (Sigma-Aldrich) and dialyzed against H-0.2 (25 mM Tris-HCl (pH 7.4), 0.5 mM EDTA, 10% glycerol, 3 mM β-mercaptoethanol and 200 mM NaCl) after the addition of TEV protease at a 1:25 protease:protein ratio. The dialyzed protein was loaded on a second Ni^2+^ resin for TEV protease removal and the flow-through was collected. Further purification was conducted over a HiLoad 16/60 Superdex 200 pg gel filtration column (GE Healthcare) in buffer H-0.2 lacking glycerol with the addition of 2 mM dithiothreitol instead of β-mercaptoethanol. The purity was estimated at ∼95% following SDS-PAGE gel electrophoresis and staining with Coomassie (Supplementary Figure S1A). RNA EMSA were performed as previously described (19).

### Mitoribosome profiling

The ribosome profiling experiment was performed as described previously (20), with minor modification. During separation of ribosome protected fragments (RFPs) on 15% denaturing polyacrylamide gel, a wider range of RNA species (migrating between 10 and 50 nt) was excised from the gel. Computational analysis is described in Supplementary Data.

### Statistics

The data represented from oxygraph and growth curve are derived from the mean values. Error bars represent the standard deviation. Statistical analyses were performed as two-tailed, unpaired-unequal variance Student’s *t*-tests. The significance threshold was set at *P* < 0.05; indicated as * for *P* < 0.05, ** for *P* < 0.01 and *** for *P* < 0.001.

## RESULTS

### C6orf203 has a predicted S4-like RNA-binding domain

**Figure 1.**
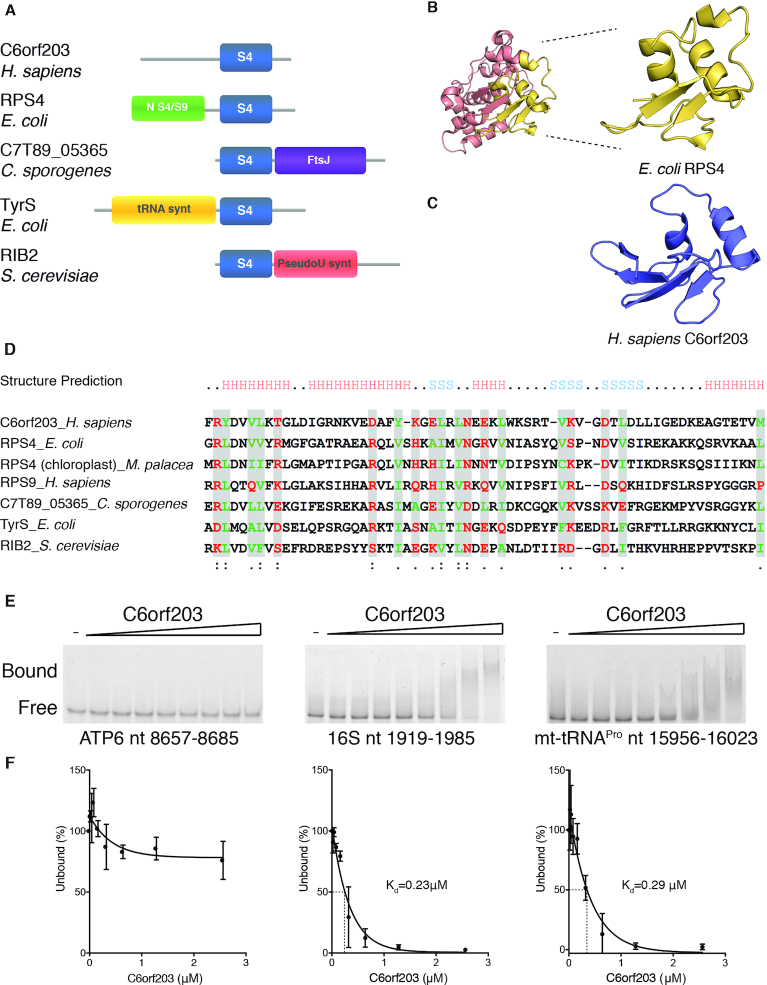
C6orf203 contains an S4-like RNA-binding domain (**A**). Domain architecture of a diverse selection of proteins containing S4-like RNA-binding domains, including *Homo sapiens* C6orf203 (Q9P0P8), *Escherichia coli* 30S ribosomal protein S4 (RPS4) (P0A7V8), *Clostridium sporogenes* NJ4 rRNA methyltransferase (A0A2P8MJ46), *E. coli* tyrosyl-tRNA synthetase (P0AGJ9) and *Saccharomyces cerevisiae* bifunctional protein RIB2 pseudouridine synthetase (Q12362). Proteins are aligned with respect to their S4-like domain (blue) with additional characterized functional domains indicated for each protein. Additional functional domains: NS4/S9 – N-terminal S4/S9 RNA binding domain; FtsJ, FtsJ-like methyltransferase; tRNA synth, tRNA synthetase class I domain (W and Y), PseudoU synt, RNA pseudouridylate synthetase. (**B**) Previously resolved structure of *E. coli* ribosomal protein S4 (PDB: 3J9Y). The C-terminal S4-like RNA-binding domain is enlarged and highlighted in yellow. (**C**) Structural prediction of the S4-like RNA-binding domain of human C6orf203. Prediction was generated in SWISS-MODEL (38) using the primary sequence residues 143–216 of human C6orf203 (Q9P0P8), and modeled in USCF Chimera (39). (**D**) Multiple protein sequence alignment of the S4-like RNA-binding domain of several family member proteins, including *H. sapiens* C6orf203. Alignment of protein sequence was constructed using Clustal Omega (40). The conserved positions are shaded using the 80% consensus rule. The amino acid classes used in building the consensus were as follows: red, polar residues; green, hydrophobic residues; gray boxes, small residues. Names of the proteins are as assigned in UniProt: RPS4_*E. coli*: ribosomal protein S4, *Escherichia coli* (P0A7V8); RS4 (chloroplast)_*M. palacea*: chloroplast ribosomal protein S4, *Marchantia paleacea* (P06358); RPS9_*H. sapiens*: 40S ribosomal protein S9, *Homo sapiens* (P46781); rRNA methylase_*C. sporogenes*: rRNA methyltransferase, *Clostridium sporogenes* (A0A2P8MJ46); TyrS_*E. coli*: Tyrosyl-tRNA synthetase, *Escherichia coli* (P0AGJ9); RIB2_*S. cerevisiae*: RIB2, *Saccharomyces cerevisiae* (Q12362). (**E**) RNA electrophoretic mobility shift assay (EMSA) indicating preference of C6orf203 for binding double stranded RNA. Recombinant human C6orf203 was incubated with fluorescein-labeled RNAs (100 nM). RNA template sequences used were as indicated (nt. position in mtDNA). Protein concentrations used were 0, 0.02, 0.04, 0.08, 0.16, 0.36, 0.64, 1.28, 2.56 μM, respectively. (**F**) Quantification of proportion of unbound RNA ligand relative to no addition of C6orf203 protein, as in Figure 1E. Dissociation constant (*K*_d_) of RNA ligand with C6orf203 is indicated for experiments where it could be determined.

BLAST analysis revealed an S4-like RNA-binding domain within the human C6orf203 protein (Figure 1A). This domain received its name through its initial identification in the prokaryotic integral ribosomal protein S4 (RPS4) (14). RPS4 binds to 16S rRNA in the maturing 30S subunit of the bacterial ribosome, and promotes correct rRNA folding during ribosomal subunit assembly (21), as well as binding to its own mRNA to repress translation (22). The RPS4 protein consists of two RNA-binding domains (Figure 1A), both of which form extensive contacts with rRNA in the 30S subunit (23). However, it is the C-terminal domain of RPS4, termed the S4-like RNA binding domain, which demonstrates conservation to the C-terminal domain of C6orf203 (Figure 1A). Comparison of the predicted folding of the S4-like RNA-binding domain of C6orf203 to the previously resolved S4-like RNA-binding domain of *Escherichia coli* RPS4 (PDB:3J9Y) suggests structural conservation between the two domains (Figure 1B and C), characterized by an α-helical bundle packed against anti-parallel β-sheets (23).

The S4-like RNA-binding domain consists of 60–65 amino acids, with key conserved residues coordinating RNA binding (Figure 1D). The S4-like domain has been found in a diverse range of RNA-interacting proteins across different species. The exact location of the S4-like domain can vary within protein sequence and is often found in combination with additional functional protein domains, including those which confer enzymatic activity (Figure 1A). Alignment of primary sequences of proteins across phylogeny was performed to demonstrate the diversity of S4-like domain containing proteins: *H. sapiens* ribosomal protein S9, *E. coli* tyrosyl-tRNA synthetase, *Marchantia paleacea* chloroplast ribosomal protein S4, *C. sporogenes* rRNA methyltransferase, *S. cerevisiae* bifunctional protein RIB2 pseudouridylate synthase (Figure 1A and D). The S4-like domain provides RNA-binding ability to these proteins, positioning the functional domains to their cognate RNAs (14). Whilst the biological role of these extra domains has been characterized in many of these proteins (Figure 1A), our attempts to predict previously characterized functional domains in C6orf203, aside from the S4-like domain, through either BLAST search or iTASSER prediction (Iterative Threading ASSEmbly Refinement (24) ), were unsuccessful.

With evidence suggesting that C6orf203 contains a functional S4-like RNA binding domain, we sought to determine if recombinant human C6orf203 possesses the ability to bind RNA *in vitro* by performing electrophoretic mobility shift assays (EMSA). EMSA did not indicate affinity of C6orf203 to a fluorescein-labeled single stranded (ss)RNA template (a portion of ATP6 mRNA, mtDNA nt. 8657–8685) (Figure 1E and F). In contrast, when incubated with double-stranded (ds)RNA templates, either portions of 16S mt-rRNA (mtDNA nt. 1919–1985) or mt-tRNA^Pro^ (mtDNA 15956–16023), C6orf203 demonstrated binding affinity (Figure 1E and F), with *K*_d_ values of 0.23 and 0.29μM, respectively. In addition, we repeated our C6orf203 EMSA with fluorescein-labeled mt-tRNA^Pro^ dsRNA in the presence of either unlabeled ssRNA or structured dsRNA molecules at a concentration 100-fold higher than the labeled mt-tRNA^Pro^ (Supplementary Figure S1B). Whilst the unlabeled mt-tRNA^Pro^ dsRNA was successful in competing with the labeled RNA, preventing binding of C6orf203, no such competition was observed for the unlabeled ssRNA template, supporting the notion that C6orf203 preferentially binds structured RNA over linear templates. Together, these data suggest that C6orf203 possesses RNA-binding ability, with specificity for binding ds and/or highly structured RNA.

### C6orf203 localizes to the mitochondrial matrix

**Figure 2.**
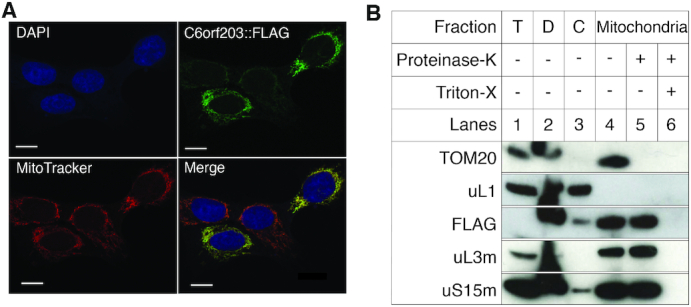
C6orf203 localizes to the mitochondrial matrix in human cells. (**A**) Intracellular localization of C6orf203 via immunocytochemistry. *C6orf203::FLAG* cDNA was transiently transfected into HOS cells. Cell nuclei were stained with DAPI (blue). The C6orf203::FLAG protein product was detected via an anti-FLAG antibody and visualized using a secondary antibody conjugated to Alexa Fluor 488 (green). Mitochondria were visualized using MitoTracker Red CMXRos (red). A digitally merged image of DAPI, Alexa Fluor 488 and MitoTracker signals reveals colocalization of C6orf203::FLAG with the mitochondrial network; scale bar = 10 μm. (**B**) Subcellular fractionation of HEK293T cells expressing C6orf203::FLAG protein. HEK293T cell lysates were fractionated into cytosolic and mitochondrial fractions. In addition, aliquots of the mitochondrial fractions were treated with 25 μg/ml Proteinase K with or without treatment with 1% Triton X-100. Fractions (40 μg) were analyzed by western blotting and the localization of C6orf203::FLAG was assessed in comparison to that of protein markers of the cytosol (cytosolic ribosomal protein uL1), outer mitochondrial membrane (TOM20), and mitochondrial matrix (mitochondrial ribosomal protein uL3m and uS15m). T, total cell lysate; D, debris; C, cytosolic fraction.

We next sought to characterize the subcellular localization of C6orf203. According to the MitoCarta 2.0 inventory, C6orf203 is predicted to localize to mitochondria in humans (13). In order to confirm the prediction, we performed immunocytochemistry (ICC) of human 143B osteosarcoma (HOS) cells transiently transfected with cDNA of C6orf203 tagged with a C-terminal FLAG epitope tag (C6orf203::FLAG). Resultant ICC images indicated strong colocalization of C6orf203::FLAG with MitoTracker Red CMXRos (Figure 2A), indicating a predominantly mitochondrial localization of the recombinant C6orf203::FLAG protein.

We next generated a HEK293T cell line, which allowed for inducible overexpression of C6orf203::FLAG via the Flp-In TREx system (Invitrogen). Subcellular fractionation of HEK293T cells overexpressing C6orf203::FLAG further supported our ICC findings that C6orf203 localizes to human mitochondria (Figure 2B). Although FLAG signal for the C6orf203::FLAG was too weak to detect in total lysate (T), C6orf203::FLAG was evident in the mitochondrial fraction, indicating an enriched localization of the protein to mitochondria (Figure 2B). Following Proteinase K treatment of isolated mitochondria, C6orf203::FLAG remains intact, similar to the behavior of mitoribosome subunit proteins uL3m and uS15m, while cytosolic ribosomal protein uL1 and outer mitochondrial membrane protein TOM20 are both degraded by Proteinase K treatment (Figure 2B). This indicates that the C6orf203::FLAG protein remains protected from proteolysis within mitochondria. Together, these results confirm that C6orf203 localizes to mitochondria in human cultured cells.

### Mitochondrial gene expression is perturbed in the absence of C6orf203

**Figure 3.**
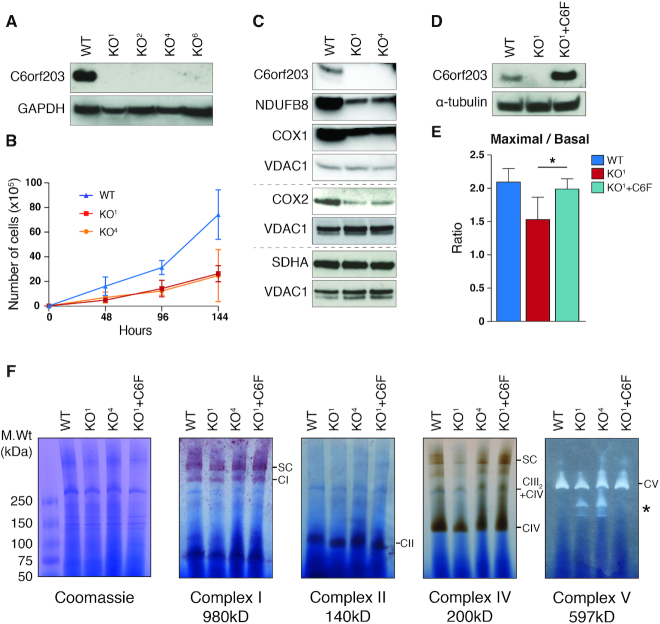
Mitochondrial gene expression is perturbed in the absence of C6orf203. (**A**) Western blotting to confirm knockout of C6orf203 in HEK293T lines KO^1^, KO^2^, KO^4^ and KO^6^. Total lysate from WT and KO clones was resolved via SDS-PAGE, immunoblotting was performed and membranes were probed with anti-C6orf203 antibody and anti-GAPDH as a loading control. (**B**) Growth curve of WT HEK293T, and C6orf203 KO cells in DMEM containing 0.9 g/l galactose (three biological replicates were performed and mean average cell numbers at each time point is indicated, error bars = 1 SD). (**C**) Western blotting of OXPHOS subunits in C6orf203 KO lines. Total lysate from WT HEK293T and C6orf203 KO^1^ and KO^4^ was resolved via SDS-PAGE; Western blotting was performed and membranes were probed with antibodies against C6orf203 and both mtDNA-encoded (COX1, COX2) and nuclear-encoded (NDUFB8, SDHA, ATP5a) OXPHOS subunits. Anti-VDAC1 antibody was used as loading control. (**D**) Western blotting was performed on the control HEK293T (WT), C6orf203 KO^1^ cells and KO^1^ cells expressing C6orf203::FLAG (KO^1^+C6::F) that were used for Oxygraph in (E). Antibody staining against C6orf203 was performed to confirm knockout of C6orf203, and re-expression of the C6orf203::FLAG protein. α-tubulin was used as loading control. (**E**) Ratio of maximal and basal mitochondrial respiration (each expressed in pmol of oxygen flux/mg of protein) measured by Oroboros oxygraph. Maximal respiration was measured after permeabilization of the cells (with Digitonin), inhibition of complex V (with Oligomycin) and treatment with the protonophore (CCCP) (*n* = 3, * *P*-value = 0.0308). (**F**) BN-PAGE and in-gel activities of complex I, complex II, complex IV and complex V activities in mitochondrial protein extracts from WT, C6orf203 KO clones and KO^1^ cells expressing C6orf203::FLAG (KO^1^+ C6::F). Coomassie staining of the gel is shown to indicate equal loading. Asterisk indicates accumulated F_1_-containing sub-complexes of complex V.

As the EMSA results have suggested that C6orf203 may have RNA-binding capability (Figure 1E), we sought to explore the role of C6orf203 in mitochondrial gene expression. To investigate this, several knockout HEK293T cell lines (KOs) were produced using CRISPR/Cas9 technology (15) and knockout was confirmed via western blotting (Figure 3A) and PCR analysis (Supplementary Figure S2B). KO^1^ and KO^4^ were chosen for future analysis.

To assess mitochondrial function in the C6orf203 knockout lines, clones were cultured in media containing galactose as the sole carbon source, which forces cells to rely almost entirely on mitochondria for ATP production. A reduced ability of KO clones to proliferate in galactose media relative to the growth of wild-type (WT) HEK293T cell line population was observed (Figure 3B), suggesting a mitochondrial dysfunction.

To further investigate this observation, western blotting of OXPHOS subunits was performed, indicating a mild reduction in the steady-state levels of components of complex I (NDUFB8) and IV (COX1 and COX2) (Figure 3C), suggesting that the C6orf203 knockout interferes with mitochondrial gene expression.

In order to confirm that the observed OXPHOS deficiency was due to the loss of the C6orf203 protein, we complemented the KO^1^ clone via the Flp-In T-Rex system to allow for inducible expression of wild-type C6orf203::FLAG cDNA (Figure 3D). We next measured oxygen consumption rates in knockout and complemented cell lines. The ratio of maximal respiration to basal respiration was modestly decreased in the absence of C6orf203 (Figure 3E), which was in line with the moderate decrease in the steady-state levels of components of OXPHOS complexes. This mild decrease was rescued by re-expression of C6orf203::FLAG protein, also confirming that FLAG-tagged protein can functionally substitute endogenous C6orf203.

In addition, in-gel activity of OXPHOS complexes were analyzed for both knockout and complemented lines (Figure 3F). Activity of complexes I, II and IV was unchanged or mildly affected for tested KO clones (Figure 3F). However, upon staining for in-gel activity of complex V, we observed activity derived from complexes of a reduced molecular mass relative to the F_1_F_0_ holoenzyme (Figure 3F). Due to the presence of ATP hydrolysis, these are likely formed due to F_1_-containing subcomplexes. This suggests that a mild assembly defect is occurring in complex V in the absence of C6orf203, which is rescued upon the re-expression of *C6orf203::FLAG* cDNA (Figure 3F). Together, these results support that C6orf203 contributes to efficient OXPHOS integrity within mitochondria, due to the mild phenotype present upon C6orf203 ablation.

**Figure 4.**
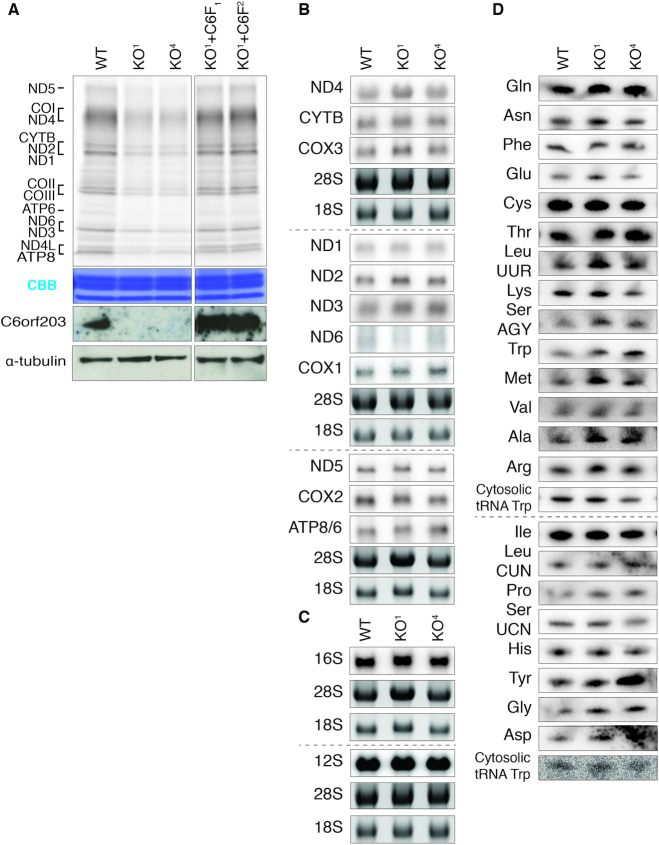
Knockout of C6orf203 leads to decreased mitochondrial translation without affecting the steady-state levels of mitochondrial RNAs. (**A**) Mitochondrial translation in C6orf203 KO cell lines. Following inhibition of cytosolic translation, products of mitochondrial translation were labeled with [^35^S]-methionine in WT, C6orf203 KO clones and KO^1^ cells expressing C6orf203::FLAG (KO^1^+C6::F, numbers indicate clones 1 and 2). Mitochondrial proteins were separated by SDS-PAGE and visualized by autoradiography. Coomassie Brilliant Blue (CBB) staining is provided to confirm equal protein loading. Immunoblotting was used to show C6orf203 expression. (**B**) Northern blotting of mt-mRNAs (B), mt-rRNAs (**C**) and mt-tRNAs (**D**) for WT and C6orf203 KO clones. Nuclear-encoded 28S and 18S rRNA were used as a loading control for mt-mRNAs and mt-rRNAs, and cytosolic tRNA (Tryptophan) was used as a loading control for mt-tRNA blots.

In light of the localization of C6orf203 to mitochondria, we sought to determine if the mild OXPHOS phenotype in C6orf203 KO was directly attributable to a defect in mtDNA expression. Metabolic labeling of mitochondrial translation products indicated a strong, general translation defect in independent KO clones (about 50% compared to control, *n* = 3), which was restored upon expression of *C6orf203::FLAG* cDNA in KO^1^ (Figure 4A). As our earlier findings (Figures 1E and 2) suggest that C6orf203 may function as a mitochondrial RNA-binding protein, we next investigated whether alteration to the mitochondrial transcriptome was an underlying factor to the mitochondrial translation defect observed in C6orf203 KO. However, northern blotting of all mtDNA-encoded mRNAs, rRNAs and tRNAs (Figure 4B, C and D) did not indicate any change to steady-state levels, nor disturbance in RNA processing, that would explain the severe, general mitochondrial translation defect observed.

### C6orf203 interacts with the mitochondrial ribosomal large subunit

**Figure 5.**
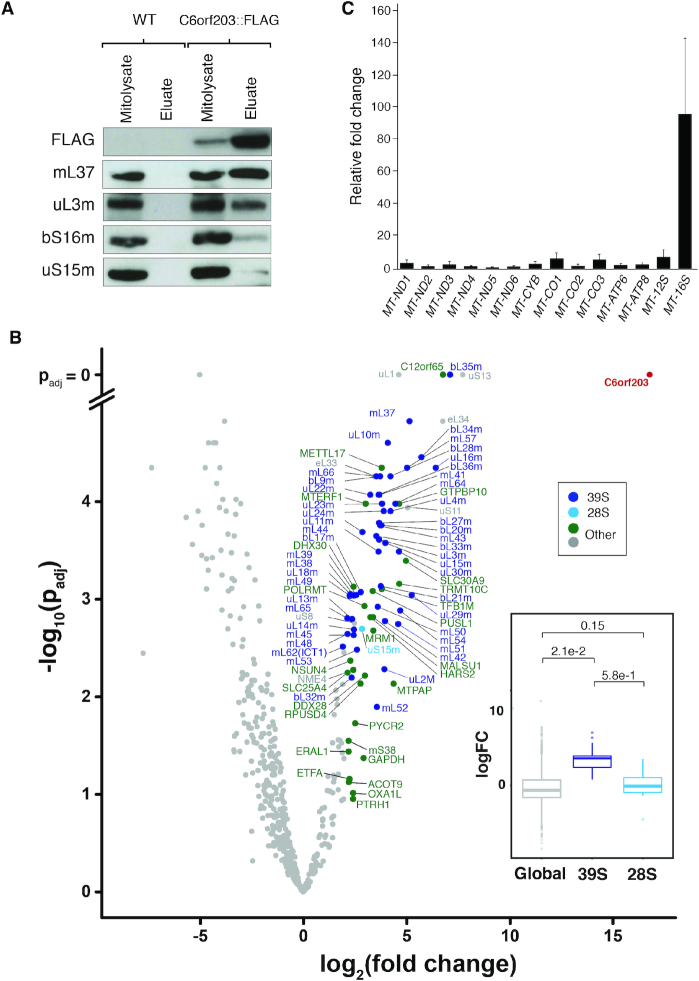
C6orf203 interacts with the mt-LSU. (**A**) Immunoblotting of C6orf203::FLAG pulldown. Input mitochondrial lysates and eluate of FLAG-IP from HEK293T expressing C6orf203::FLAG and control HEK293T without FLAG protein expression (WT) were resolved via SDS-PAGE; western blotting was performed and subsequent membranes were probed with antibodies against FLAG and for proteins of either the mt-LSU proteins (mL37, uL3m) or mt-SSU proteins (bS16m, uS15m). (**B**) Mass spectrometry analysis of proteins interacting with C6orf203::FLAG. Following FLAG-immunoprecipitation, eluates were analyzed by label-free quantitative mass spectrometry (LFQ) (*n* = 3). Volcano plot indicates only proteins found in the Mitocarta 2.0 database. Proteins with fold change >2.5 are marked. Inset: Boxplot displaying comparison of the logFC (log fold change) of proteins (as in main B) of the mt-LSU or mt-SSU in comparison to global proteins. Stated *P*-values indicate pair-wise significance of difference in logFC between global proteins, 39S mt-LSU proteins or 28 mt-SSU proteins as determined via Welch’s unequal variances *t*-test. (**C**) Quantitative real-time PCR to assess enrichment of mt-RNAs upon C6orf203::FLAG pulldown. Anti-FLAG Immunoprecipitation was performed on mitochondrial lysate from HEK293 cells and HEK293T cells overexpressing C6orf203::FLAG. RNA was extracted from both mitochondrial lysates and elution fractions, reverse transcribed, and qPCR was performed using primer and probes pairs to mitochondrial DNA encoded transcripts. The relative fold change of transcript abundance in the C6orf203::FLAG immunoprecipitated eluates versus the HEK293 eluates relative to transcript abundance in their respective input mitochondrial lysates were calculated and are shown here. Graph represents combination of three biological replicate experiments; error bars = S.E.M.

As no steady-state level change of any of the mitochondrial transcripts was observed, we next sought to identify specific interactions of C6orf203, as this may provide more insight into the underlying mechanism of the observed mitochondrial translation defect. Following C6orf203::FLAG induction in our overexpressing HEK293T line, FLAG immunoprecipitation (IP) without crosslinking was performed. To control for non-specific binding, FLAG-tagged mitochondrially targeted luciferase was expressed in HEK293T and immunoprecipitated by the same procedure (Supplementary Figure S3B). Western blotting of the eluate revealed specific enrichment of proteins of the mt-LSU (mL37 and uL3m) in C6orf203 pulldown, while protein subunits of the mt-SSU were not enriched (Figure 5A). Additionally, we found that the observed interaction with mt-LSU subunits was RNA-dependent, since RNase A treatment led to a loss of interaction of C6orf203 with mL37 and uL3m (Supplementary Figure S3C).

Next, we performed label-free quantitative mass spectrometry on the eluate from the C6orf203::FLAG IP relative to a negative control (HEK293T without FLAG protein expression). This revealed enrichment of a large number of proteins of the mt-LSU that co-immunoprecipitated with C6orf203::FLAG (Figure 5B), while only a single subunit of the mt-SSU was observed (uS15m) with a logFC of >2. Indeed, when the enrichment profiles of the constituent proteins from the mt-LSU and the mt-SSU were considered separately, all mt-LSU subunits showed a consistent enrichment over global protein distribution, while mt-SSU proteins were not enriched over that of global proteins (Figure 5B, inset). We also observed enrichment of several cytosolic ribosomal proteins. However, this interaction was reduced upon Proteinase K treatment of mitochondria prior to IP, as tested via western blotting (Supplementary Figure S3A).

In addition to mt-LSU components, a number of other proteins were highly enriched in C6orf203::FLAG IP, including a number of factors known to be involved in assembly of the mt-LSU (i.e. GTPBP10, DHX30, MALSU1, RPUSD4, TRMT10C) (Figure 5B). As many of these assembly factors would likely be excluded from the mature ribosome, this may indicate that the observed interaction of C6orf203 occurs within an assembly intermediate of the mt-LSU.

Curiously, one of the most highly enriched proteins observed upon C6orf203::FLAG pulldown was C12orf65, a member of a family of four mitochondrial class I peptide release factors. However, the exact role of C12orf65 in mitochondrial translation is currently unknown, with family member mtRF1a having been found to be sufficient for termination of translation in all 13 mitochondrial open reading frames (25). The interaction of C12orf65 with C6orf203 requires further investigation to determine functional relevance.

As we have earlier characterized C6orf203 as having RNA-binding affinity, we sought to investigate whether C6orf203 interacted with mitochondrial RNAs and so repeated our C6orf203::FLAG immunoprecipitations and extracted RNA from both input and eluates for the C6orf203::FLAG line and WT HEK293 control. Through quantitative RT-PCR focused on mt-mRNAs and mt-rRNAs, we observed specific enrichment of 16S mt-rRNA upon C6orf203::FLAG IP compared to control (Figure 5C). This observation further supports our proteomic findings concerning interaction of C6orf203 with either the full or a near-assembled intermediate of mt-LSU.

### Absence of C6orf203 leads to reduction of mt-mRNAs loaded onto mitoribosomes

**Figure 6.**
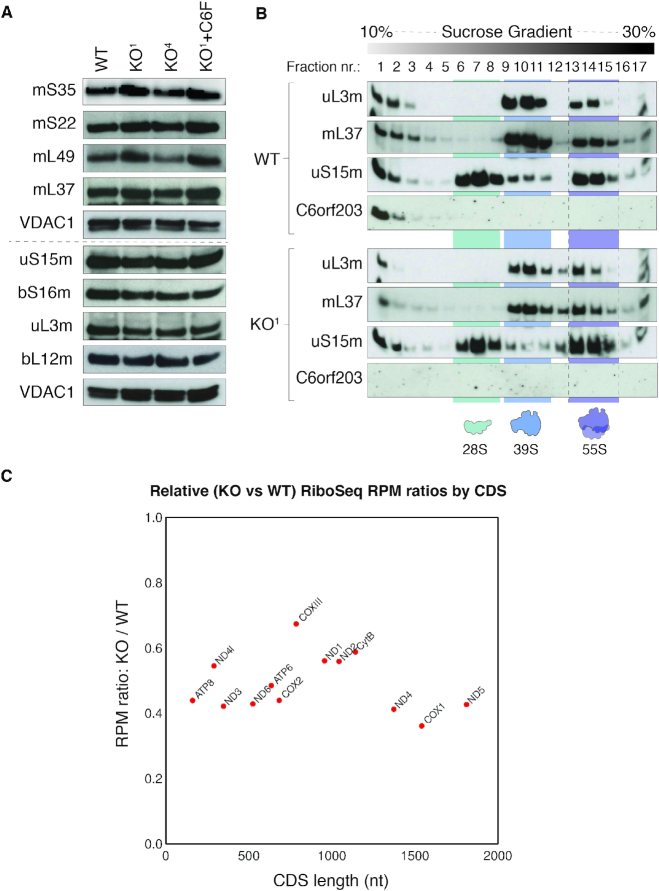
C6orf203 loss affects the engagement of mt-mRNAs with mitoribosomes. (**A**) Western blotting of steady-state levels of mitochondrial ribosomal proteins in WT, C6orf203 KO and KO^1^ cells expressing C6orf203::FLAG (KO^1^+C6::F). Total cell lysate from WT HEK293T, C6orf203 KO clones and KO^1^ cells expressing C6orf203::FLAG (KO^1^+C6F) was resolved via SDS-PAGE; Western blotting was performed and membranes were probed with antibodies against proteins of either the mt-LSU or mt-SSU, and VDAC1 was used as loading control. (**B**) Sedimentation of mitochondrial ribosomes on 10–30% isokinetic sucrose gradients for WT HEK293T and C6orf203 KO^1^. Mitochondria were isolated from cells, and lysates were loaded onto gradients. Following centrifugation, obtained fractions were analyzed by western blotting with antibodies against proteins of the mt-LSU (uL3m and mL37), the mt-SSU (uS15m) and C6orf203. (**C**) Mitoribosome profiling analysis. Relative ratio of mitoribosome-protected fragments (per million mapped reads, RPM) for C6orf203 KO^1^ versus WT HEK293T for each mt-mRNA ORF determined via MitoRiboSeq. Individuals CDSs are displayed according to their ORF length. Reads with 5′ ends mapping between the first nucleotide of the start codon and 30 nt 5′ of the stop codon were counted for each library. Overlapping regions for ORFs on bicistronic transcripts (ATP8/ATP6 and ND4l/ND4) were excluded from the analysis. Results represents data from a single MitoRiboSeq experiment.

In light of the observed interaction between C6orf203 and the mt-LSU, as well as the enrichment of a complement of known mt-LSU assembly factors (Figure 5B), we next sought to assess the integrity of the mitoribosomes in our C6orf203 KO lines. Western blotting revealed no decrease of steady-state levels of a number of constituent proteins of either the mt-LSU or mt-SSU (Figure 6A), and sucrose gradient fractionation indicated that overall integrity of both the mt-SSU and mt-LSU was retained in the C6orf203 KO clones (Figure 6B). This suggests that there is no global defect in the assembly of either subunit of the mitoribosome in the absence of C6orf203 (Figure 6B), and so C6orf203 is unlikely to play a role in the early stages of assembly of the mt-LSU. This is consistent with the observation of a near-full complement of mt-LSU proteins upon C6orf203::FLAG pulldown (Figure 5B), suggesting instead that C6orf203 may act on a later stage assembly intermediate of mt-LSU or the full mt-LSU.

Curiously, despite the observed translational defect in C6orf203 KO cells (Figure 4A), mitochondrial monosome formation is not affected in the absence of C6orf203 (Figure 6B). To investigate this disparity between mitochondrial monosome formation and the strong translation defect present in C6orf203 KO, we employed mitochondrial ribosome profiling (MitoRiboSeq) in one of the C6orf203 KO lines (KO^1^). Through this, we observed that occupancy of all mt-mRNAs on mitochondrial monosomes was greatly reduced relative to WT HEK293T (Figure 6C), although no specific mt-mRNAs were affected to a greater extent than others (Figure 6C). The MitoRiboSeq results are in accordance with the mitochondrial translation defect observed in C6orf203 KO (Figure 4A).

Further analysis of MitoRiboSeq revealed that, although the engagement of mRNAs with mitoribosomes is decreased, once the mRNAs are loaded and translation initiated, there is no major disturbance in the elongation efficiency, as no obvious difference in mitoribosome pausing was observed between KO and WT cells (Supplementary Figure S4A and B), nor mitoribosome drop-off across the length of translated transcripts (which could indicate spurious loss of reading frame) (Supplementary Figure S4C). Additionally, we did not observe any increase in occupancy of the mitoribosomes on specific codons in the C6orf203 knockout lines, which suggests that there is no perturbation of the availability of any individual aminoacylated mt-tRNA (Supplementary Figure S5). Taken together, in the absence of C6orf203, mitochondrial monosomes are formed, but their function is compromised, resulting in reduced mitochondrial translation.

## DISCUSSION

Many aspects of mitochondrial gene expression remain elusive, including assembly of the mitoribosome and regulation of mitochondrial translation. These processes require the assistance of multiple trans-acting factors, and in recent years, the identity of a number of proteins involved have been discovered (26). Here, we present the initial characterization of the role of the putative RNA-binding protein C6orf203 in mitochondrial gene expression, suggesting that C6orf203 can be added to the growing group of factors that interact with the mitoribosome and are required for efficient mitochondrial translation.

In our study, knockout of C6orf203 in HEK293T did not lead to a strong OXPHOS deficiency, despite the mitochondrial translation defect observed. This milder phenotype may explain why *C6orf203* has not previously been identified as an essential gene for OXPHOS (27).

In the C6orf203 knockout cell lines, we saw defects in in-gel activity only for complex V, in the form of sub-assemblies, which retained ATP hydrolysis, whilst the activity of other OXPHOS complexes was largely unaffected (Figure 3F). It has previously been noted that such sub-assemblies of complex V are rather a sensitive marker of overall mitochondrial gene expression, as opposed to a specific defect in mtDNA-encoded complex V subunit expression (28). Indeed, our MitoRiboSeq data do not suggest that the translation of ATP6 and ATP8 is disproportionately affected when compared to that of other mt-mRNA ORFs (Figure 6C). Therefore, we conclude that our observed defect to complex V is likely to be due to the nature of sensitivity of ATP synthase assembly to a reduction in mitochondrial gene expression, and not a specific effect of the loss of C6orf203 function.

### C6orf203 is a conserved mitochondrial RNA-binding protein that interacts with the mt-LSU

An NCBI SMARTBLAST (https://blast.ncbi.nlm.nih.gov/smartblast/) using the primary sequence of human C6orf203 revealed homologues with high levels of identity not only in vertebrates, but across the whole animal kingdom (Supplementary Figure S6). As with human C6orf203, each of these proteins are predicted to possess a conserved S4-like RNA-binding domain, suggesting functional conservation.

Many of the proteins identified thus far to contain the S4-like RNA-binding domain interact with highly structured RNA, such as the prokaryotic ribosomal protein S4 that interacts with 16S rRNA (29) or tyrosyl-tRNA synthetase that binds to tRNA^Tyr^ (14). Indeed, our EMSA assays indicated a preference of human C6orf203 to bind structured RNA *in vitro*, displaying higher affinity to a portion of 16S mt-rRNA and a portion of mt-tRNA^Pro^, both predicted to form dsRNA, as compared to a single-stranded section of ATP8/6 mt-mRNA. This preference for binding dsRNA was previously observed upon application of EMSA for other mitochondrial proteins known to bind the mitoribosome, mTERF3 and the NSUN4/MTERF4 complex (30,31). Of particular note, the NSUN4/MTERF4 complex displayed no affinity for ssRNA but was able to bind predicted dsRNA portions of 16S mt-rRNA (31), similar to the behavior we have observed here for C6orf203 (Figure 1E).

With EMSA suggesting that C6orf203 has the capacity to bind RNA *in vitro*, determining the exact target RNAs of C6orf203 in the mitochondrial transcriptome would be advantageous to discern the exact function of C6orf203. Whilst it cannot be excluded that C6orf203 has multiple binding sites in the mitochondrial transcriptome, the enrichment of core mt-LSU proteins upon C6orf203::FLAG-IP may indicate C6orf203 binding to RNA integral to the mt-LSU i.e. 16S mt-rRNA, or possibly mt-tRNA^Val^ incorporated into mt-LSU (32), supported by our observed enrichment of 16S mt-rRNA in IP eluates, determined via qRT-PCR (Figure CC).

In support of our observation of direct interaction of C6orf203 with the mtLSU, C6orf203 was observed in the previously published study where interacting partners of integral mitochondrial ribosomal protein ICT1 (mL62) were identified (18). As ICT1 is an integral protein of mt-LSU, it is likely that the observed interaction of ICT1 with C6orf203 in this study is mediated through an mt-LSU complex.

Indeed, the interaction of C6orf203 with mt-LSU subunits was found to be RNA dependent, as RNase treatment led to loss of interaction with mt-LSU proteins (Supplementary Figure S3C). Whilst this does not necessarily indicate that C6orf203 binds directly to RNA to facilitate binding to mt-LSU, it does indicate that the association of C6orf203 to at least some mt-LSU proteins observed in the FLAG-IP (Figure 5) are likely mediated through an intact mt-LSU subunit or assembly intermediate.

The mitochondrial translation defect in the absence of C6orf203, assessed via both [^35^S]-Met labeling and MitoRiboSeq profiling, is global in nature with translation of all mtDNA-encoded open reading frames affected to a similar extent. If the primary mechanism of C6orf203 knockout was mediated through the lack of C6orf203 action on a specific mt-tRNA or mt-mRNA species, it is anticipated that a more specific effect on translation would be observed, either by reduced translation of affected mRNAs or reduced use of tRNAs. As we did not observe these specific changes, a loss of interaction of C6orf203 with mt-LSU, therefore, has the greatest potential to be the underlying primary defect in the global mitochondrial translation phenotype observed.

Interestingly, despite specific enrichment of core mt-LSU proteins observed in our FLAG::IP experiments, we did not observe co-sedimentation of the C6orf203 protein with either the mt-LSU or monosome in our sucrose gradient fractionation experiments (Figure 6B). However, this observation does not exclude that C6orf203 may bind to mt-LSU or a late stage assembly intermediate, as previously the interactions of the factors that transiently associate with the mitochondrial ribosome (e.g. mtRRF and GTPBP10) were also not seen in standard sucrose gradient fractionations, but were detected in immunoprecipitation experiments (33, 34-for HEK293T cells). This could instead indicate that the interaction of C6orf203 with mt-LSU is either transient in nature, or difficult to capture in the experimental conditions employed in our sucrose gradient fractionation.

### Role of C6orf203 in mitoribosome function

Through bioinformatic analysis, we failed to attribute any previously characterized enzymatic functionality to the N-terminal portion of C6orf203. It is possible instead that portions of the C6orf203 protein beyond the S4-like domain are required for co-ordinating RNA binding, or for enabling further protein–protein interactions. For example, the resolved structure of the bacterial ribosome (PDB: 4V7U) indicates that *E. coli* ribosomal protein S4 facilitates RNA binding via amino acid residues throughout the protein sequence, not just limited to the S4-like domain (23,29). Through this extensive RNA binding, RPS4 acts at multiple stages in bacterial ribosome assembly to aid the folding of a highly structured conformation of 16S rRNA (21). More in-depth analysis into the exact residues of C6orf203 required for RNA binding, through either amino-acid substitution, or by obtaining high-resolution structures of C6orf203 bound to mt-LSU, would reveal the contribution of specific C6orf203 residues for mt–RNA interaction.

Alternatively, it is possible that the N-terminal portion of C6orf203 is required for recruiting further protein factors to the mt-LSU. In this manner, C6orf203 binding may aid directly in recruitment of mt-LSU proteins or other assembly factors to maturing late stage assembly intermediates, or through priming RNA structure for protein binding. This function may be especially important in light of the increased protein complement in the human mitochondrial ribosome as compared to the prokaryotic ribosome (10).

Despite the observed defect in mitochondrial translation upon C6orf203 knockout, sucrose gradient fractionation indicated that 55S monosomes are still formed. However, MitoRiboSeq revealed a reduced amount of mRNA associating with these monosomes, suggesting a problem with mRNA loading or its increased dissociation from the 55S prior to translation elongation.

Previously, a knockout mouse model of SLIRP, a protein which binds to and stabilizes mRNA-binding protein LRPPRC, led to a loss of engagement of mt-mRNAs with the mitochondrial monosome in liver (35). In this case, despite the observation that mitochondrial monosome assembly is unaffected, the engagement of mRNAs with the mitoribosomes is impaired, leading to decreased mitochondrial translation. Together, the SLIRP study along with our findings herein support the idea that the mitochondrial monosome can exist without mRNA being required to be loaded onto it. In bacteria, initiation of translation by 70S monosome is a frequent mode of translation of non-canonical transcripts (leaderless and polycistronic mRNAs) (36,37). Considering the fact that mitochondrial mRNAs are leaderless, it is tempting to speculate that also in mitochondria, loading of mRNA is initiated by the monosome, not the mt-SSU, and C6orf203 action on mt-LSU is necessary to generate functionally competent 55S ready for translation initiation.

In conclusion, we identify C6orf203 as a novel RNA-binding protein that interacts with the mt-LSU and is required for efficient translation in human mitochondria. Further structural studies would be invaluable in discerning both the interaction site of C6orf203, and whether an as-yet-uncharacterized late stage assembly intermediate of mt-LSU is present in the absence of C6orf203.

## DATA AVAILABILITY

Sequencing data for MitoRibo-Seq have been deposited in Gene Expression Omnibus (GEO, https://www.ncbi.nlm.nih.gov/geo/) under the accession number GSE133315.

## Supplementary Material

gkz684_Supplemental_Files

## References

[1] Hentze M.W., Castello A., Schwarzl T., Preiss T. A brave new world of RNA-binding proteins. Nat. Rev. Mol. Cell Biol. 2018; 19:327–341. 29339797 10.1038/nrm.2017.130

[2] Castello A., Fischer B., Eichelbaum K., Horos R., Beckmann B.M., Strein C., Davey N.E., Humphreys D.T., Preiss T., Steinmetz L.M. et al. Insights into RNA biology from an atlas of mammalian mRNA-Binding proteins. Cell. 2012; 149:1393–1406. 22658674 10.1016/j.cell.2012.04.031

[3] Baltz A.G., Munschauer M., Schwanhäusser B., Vasile A., Murakawa Y., Schueler M., Youngs N., Penfold-Brown D., Drew K., Milek M. et al. The mRNA-Bound proteome and its global occupancy profile on protein-coding transcripts. Mol. Cell. 2012; 46:674–690. 22681889 10.1016/j.molcel.2012.05.021

[4] Castello A., Fischer B., Hentze M.W., Preiss T. RNA-binding proteins in Mendelian disease. Trends Genet. 2013; 29:318–327. 23415593 10.1016/j.tig.2013.01.004

[5] Lukong K.E., Chang K. wei, Khandjian E.W., Richard S. RNA-binding proteins in human genetic disease. Trends Genet. 2008; 24:416–425. 18597886 10.1016/j.tig.2008.05.004

[6] Corbett A.H. Post-transcriptional regulation of gene expression and human disease. Curr. Opin. Cell Biol. 2018; 52:96–104. 29518673 10.1016/j.ceb.2018.02.011 PMC5988930

[7] Rorbach J., Minczuk M. The post-transcriptional life of mammalian mitochondrial RNA. Biochem. J. 2012; 444:357–373. 22642575 10.1042/BJ20112208

[8] Jourdain A.A., Boehm E., Maundrell K., Martinou J.C. Mitochondrial RNA granules: Compartmentalizing mitochondrial gene expression. J. Cell Biol. 2016; 212:611–614. 26953349 10.1083/jcb.201507125 PMC4792075

[9] O’Brien T.W., Kalf G.F. Ribosomes from rat liver mitochondria. I. Isolation procedure and contamination studies. J. Biol. Chem. 1967; 242:2172–2179. 6022863

[10] Amunts A., Brown A., Toots J., Scheres S.H.W., Ramakrishnan V. The structure of the human mitochondrial ribosome. Science. 2015; 348:95–98. 25838379 10.1126/science.aaa1193 PMC4501431

[11] De Silva D., Tu Y.T., Amunts A., Fontanesi F., Barrientos A. Mitochondrial ribosome assembly in health and disease. Cell Cycle. 2015; 14:2226–2250. 26030272 10.1080/15384101.2015.1053672 PMC4615001

[12] Bogenhagen D.F., Ostermeyer-Fay A.G., Haley J.D., Garcia-Diaz M. Kinetics and mechanism of mammalian mitochondrial ribosome assembly. Cell Rep. 2018; 22:1935–1944. 29444443 10.1016/j.celrep.2018.01.066 PMC5855118

[13] Calvo S.E., Clauser K.R., Mootha V.K. MitoCarta2.0: an updated inventory of mammalian mitochondrial proteins. Nucleic Acids Res. 2016; 44:D1251–D1257. 26450961 10.1093/nar/gkv1003 PMC4702768

[14] Aravind L., Koonin E. V. Novel predicted RNA-binding domains associated with the translation machinery. J. Mol. Evol. 1999; 48:291–302. 10093218 10.1007/pl00006472

[15] Ran F.A., Hsu P.D., Lin C.-Y., Gootenberg J.S., Konermann S., Trevino A.E., Scott D.A., Inoue A., Matoba S., Zhang Y. et al. Double nicking by RNA-guided CRISPR … + SUPPL. Cell. 2013; 154:1380–1389. 23992846 10.1016/j.cell.2013.08.021 PMC3856256

[16] Minczuk M., He J., Duch A.M., Ettema T.J., Chlebowski A., Dzionek K., Nijtmans L.G.J., Huynen M.A., Holt I.J. TEFM (c17orf42) is necessary for transcription of human mtDNA. Nucleic Acids Res. 2011; 39:4284–4299. 21278163 10.1093/nar/gkq1224 PMC3105396

[17] Nijtmans L.G.J., Henderson N.S., Holt I.J. Blue Native electrophoresis to study mitochondrial and other protein complexes. Methods. 2002; 26:327–334. 12054923 10.1016/S1046-2023(02)00038-5

[18] Richter R., Rorbach J., Pajak A., Smith P.M., Wessels H.J., Huynen M.A., Smeitink J.A., Lightowlers R.N., Chrzanowska-Lightowlers Z.M. A functional peptidyl-tRNA hydrolase, ICT1, has been recruited into the human mitochondrial ribosome. EMBO J. 2010; 29:1116–1125. 20186120 10.1038/emboj.2010.14 PMC2845271

[19] Spåhr H., Rozanska A., Li X., Atanassov I., Lightowlers R.N., Chrzanowska-Lightowlers Z.M.A., Rackham O., Larsson N.G. SLIRP stabilizes LRPPRC via an RRM-PPR protein interface. Nucleic Acids Res. 2016; 44:6868–6882. 27353330 10.1093/nar/gkw575 PMC5001613

[20] Pearce S.F., Rorbach J., Van Haute L., D’Souza A.R., Rebelo-Guiomar P., Powell C.A., Brierley I., Firth A.E., Minczuk M. Maturation of selected human mitochondrial tRNAs requires deadenylation. Elife. 2017; 6:e27596. 28745585 10.7554/eLife.27596 PMC5544427

[21] Mayerle M., Woodson S.A. Specific contacts between protein S4 and ribosomal RNA are required at multiple stages of ribosome assembly. RNA. 2013; 19:574–585. 23431409 10.1261/rna.037028.112 PMC3677267

[22] Schlax P.J., Xavier K.A., Gluick T.C., Draper D.E. Translational repression of the Escherichia coli α operon mRNA. Importance of an mRNA conformational switch and a ternary entrapment complex. J. Biol. Chem. 2001; 276:38494–38501. 11504736 10.1074/jbc.M106934200

[23] Davies C., Gerstner R.B., Draper D.E., Ramakrishnan V., White S.W. The crystal structure of ribosomal protein S4 reveals a two-domain molecule with an extensive RNA-binding surface: one domain shows structural homology to the ETS DNA-binding motif. EMBO J. 1998; 17:4545–4558. 9707415 10.1093/emboj/17.16.4545 PMC1170785

[24] Zhang Y. I-TASSER server for protein 3D structure prediction. BMC Bioinformatics. 2008; 9:40. 18215316 10.1186/1471-2105-9-40 PMC2245901

[25] Soleimanpour-Lichaei H.R., Kühl I., Gaisne M., Passos J.F., Wydro M., Rorbach J., Temperley R., Bonnefoy N., Tate W., Lightowlers R. et al. mtRF1a is a human mitochondrial translation release factor decoding the major termination codons UAA and UAG. Mol. Cell. 2007; 27:745–757. 17803939 10.1016/j.molcel.2007.06.031 PMC1976341

[26] Pearce S.F., Rebelo-Guiomar P., D’Souza A.R., Powell C.A., Van Haute L., Minczuk M. Regulation of mammalian mitochondrial gene expression: recent advances. Trends Biochem. Sci. 2017; 42:625–639. 28285835 10.1016/j.tibs.2017.02.003 PMC5538620

[27] Arroyo J.D., Jourdain A.A., Calvo S.E., Ballarano C.A., Doench J.G., Root D.E., Mootha V.K. A Genome-wide CRISPR death screen identifies genes essential for oxidative phosphorylation. Cell Metab. 2016; 24:875–885. 27667664 10.1016/j.cmet.2016.08.017 PMC5474757

[28] Mourier A., Ruzzenente B., Brandt T., Kühlbrandt W., Larsson N.G. Loss of LRPPRC causes ATP synthase deficiency. Hum. Mol. Genet. 2014; 23:2580–2592. 24399447 10.1093/hmg/ddt652 PMC3990160

[29] Brodersen D.E., Clemons W.M., Carter A.P., Wimberly B.T., Ramakrishnan V. Crystal structure of the 30 S ribosomal subunit from Thermus thermophilus: structure of the proteins and their interactions with 16 S RNA. J. Mol. Biol. 2002; 316:725–768. 11866529 10.1006/jmbi.2001.5359

[30] Wredenberg A., Lagouge M., Bratic A., Metodiev M.D., Spåhr H., Mourier A., Freyer C., Ruzzenente B., Tain L., Grönke S. et al. MTERF3 regulates mitochondrial ribosome biogenesis in invertebrates and mammals. PLoS Genet. 2013; 9:e1003178. 23300484 10.1371/journal.pgen.1003178 PMC3536695

[31] Metodiev M.D., Spåhr H., Loguercio Polosa P., Meharg C., Becker C., Altmueller J., Habermann B., Larsson N.G., Ruzzenente B. NSUN4 is a dual function mitochondrial protein required for both methylation of 12S rRNA and coordination of mitoribosomal assembly. PLoS Genet. 2014; 10:e1004110. 24516400 10.1371/journal.pgen.1004110 PMC3916286

[32] Brown A., Amunts A., Bai X.C., Sugimoto Y., Edwards P.C., Murshudov G., Scheres S.H.W., Ramakrishnan V. Structure of the large ribosomal subunit from human mitochondria. Science. 2014; 346:718–722. 25278503 10.1126/science.1258026 PMC4246062

[33] Rorbach J., Richter R., Wessels H.J., Wydro M., Pekalski M., Farhoud M., Kühl I., Gaisne M., Bonnefoy N., Smeitink J.A. et al. The human mitochondrial ribosome recycling factor is essential for cell viability. Nucleic Acids Res. 2008; 36:5787–5799. 18782833 10.1093/nar/gkn576 PMC2566884

[34] Maiti P., Kim H.-J., Tu Y.-T., Barrientos A. Human GTPBP10 is required for mitoribosome maturation. Nucleic Acids Res. 2018; 46:11423–11437. 30321378 10.1093/nar/gky938 PMC6265488

[35] Lagouge M., Mourier A., Lee H.J., Spåhr H., Wai T., Kukat C., Silva Ramos E., Motori E., Busch J.D., Siira S. et al. SLIRP regulates the rate of mitochondrial protein synthesis and protects LRPPRC from degradation. PLoS Genet. 2015; 11:e1005423. 26247782 10.1371/journal.pgen.1005423 PMC4527767

[36] Moll I., Hirokawa G., Kiel M.C., Kaji A., Bläsi U. Translation initiation with 70S ribosomes: An alternative pathway for leaderless mRNAs. Nucleic Acids Res. 2004; 32:3354–3363. 15215335 10.1093/nar/gkh663 PMC443539

[37] Pech M., Yamamoto H., Qin B., Gupta R., Krause R., Yamamoto K., Albrecht R., Ueda T., Nierhaus K.H., Wittek D. 70S-scanning initiation is a novel and frequent initiation mode of ribosomal translation in bacteria. Proc. Natl. Acad. Sci. U.S.A. 2016; 113:E1180–E1189. 26888283 10.1073/pnas.1524554113 PMC4780633

[38] Waterhouse A., Bertoni M., Bienert S., Studer G., Tauriello G., Gumienny R., Heer F.T., De Beer T.A.P., Rempfer C., Bordoli L. et al. SWISS-MODEL: homology modelling of protein structures and complexes. Nucleic Acids Res. 2018; 46:W296–W303. 29788355 10.1093/nar/gky427 PMC6030848

[39] Pettersen E.F., Goddard T.D., Huang C.C., Couch G.S., Greenblatt D.M., Meng E.C., Ferrin T.E. UCSF Chimera - a visualization system for exploratory research and analysis. J. Comput. Chem. 2004; 25:1605–1612. 15264254 10.1002/jcc.20084

[40] Sievers F., Higgins D.G. Clustal Omega. Curr. Protoc. Bioinforma. 2014; 2014:3.13.1–3.13.16. 10.1002/0471250953.bi0313s48 25501942

