# Supplementary material for: C6orf203 is an RNA-binding protein involved in mitochondrial protein synthesis
Source: Nucleic Acids Res. 2019 Aug 9;47(17):9386–99. doi: 10.1093/nar/gkz684 (PMC6755124; doi:10.1093/nar/gkz684)
Supplement: gkz684_Supplemental_Files [file nar_47_17_9386_s1.zip › Supp Table 6_2507.pdf]

Supplementary Table S6. List of proteins pulled down by anti-FLAG-immunoprecipitation and characterised by Mass Spectrometry. HEK293T cells with no FLAG-tag was used as a control for mock immunoprecipitation. The presented protein list is in the descending order of logFC. LogFC represents the enrichment score and the adjusted p-value represents the significance score.

Protein ID	Protein name	Gene names	LogFC	Adjusted p-values	MitoCarta 2.0
Q9P0P8	Uncharacterized protein C6orf203	C6orf203	16.75	0	Yes
P40429	60S ribosomal protein L13a	RPL13A	8.78	0	No
Q9NR30	Nucleolar RNA helicase 2	DDX21	7.8	1,00E-05	No
P46779	60S ribosomal protein L28	RPL28	7.77	0	No
P62269	40S ribosomal protein S18	RPS18	7.7	0	Yes
P52272	Heterogeneous nuclear ribonucleoprotein M	HNRNPM	7.46	0	No
P47914	60S ribosomal protein L29	RPL29	7.37	1.1e-4	No
P11387	DNA topoisomerase 1	TOP1	7.28	0	No
Q9NZE8	39S ribosomal protein L35, mitochondrial (bL35m)	MRPL35	7.09	0	Yes
P62910	60S ribosomal protein L32	RPL32	7.02	0	No
P06748	Nucleophosmin	NPM1	6.9	5,00E-05	No
P62701	40S ribosomal protein S4, X isoform	RPS4X	6.88	0	No
P84098	60S ribosomal protein L19	RPL19	6.84	0	No
Q9H3J6	Probable peptide chain release factor C12orf65, mitochondrial	C12orf65	6.75	0	Yes
P49207	60S ribosomal protein L34	RPL34	6.74	1,00E-05	Yes
Q07020	60S ribosomal protein L18	RPL18	6.6	0	No
Q9NX20	39S ribosomal protein L16, mitochondrial (uL16m)	MRPL16	6.4	4,00E-05	Yes
P36578	60S ribosomal protein L4	RPL4	6.22	0	No
P62854	40S ribosomal protein S26	RPS26	5.99	2,00E-05	No
P62841	40S ribosomal protein S15	RPS15	5.95	1,00E-05	No
E9PKP7	Nucleolar transcription factor 1	UBTF	5.93	1,00E-05	No
P46776	60S ribosomal protein L27a	RPL27A	5.84	0	No
Q9BQ48	39S ribosomal protein L34, mitochondrial (bL34m)	MRPL34	5.71	3,00E-05	Yes
P15880	40S ribosomal protein S2	RPS2	5.62	1,00E-05	No
P83881	60S ribosomal protein L36a	RPL36A	5.59	0.00152	No
P26373	60S ribosomal protein L13	RPL13	5.55	0	No
P11388	DNA topoisomerase 2-alpha	TOP2A	5.47	3,00E-05	No
O95793	Double-stranded RNA-binding protein Staufen homolog 1	STAU1	5.46	2,00E-05	No
Q5SSJ5	Heterochromatin protein 1-binding protein 3	HP1BP3	5.34	4,00E-05	No
P08708	40S ribosomal protein S17	RPS17	5.31	4,00E-05	No
O76021	Ribosomal L1 domain-containing protein 1	RSL1D1	5.27	4,00E-05	No

Q9HD33	39S ribosomal protein L47, mitochondrial (uL29m)	MRPL47	5.24	9,00E-04	Yes
Q9UKD2	mRNA turnover protein 4 homolog	MRTO4	5.19	5,00E-05	No
Q13243	Serine/arginine-rich splicing factor 5	SRSF5	5.14	9.2e-4	No
Q9BZE1	39S ribosomal protein L37, mitochondrial (mL37)	MRPL37	5.13	1,00E-05	Yes
P22087	rRNA 2'-O-methyltransferase fibrillarin	FBL	5.07	4,00E-05	No
P61313	60S ribosomal protein L15	RPL15	5.06	1,00E-05	No
P62263	40S ribosomal protein S14	RPS14	5.04	1.1e-4	Yes
Q9BQC6	Ribosomal protein 63, mitochondrial	MRPL57	5.01	4,00E-05	Yes
Q08211	ATP-dependent RNA helicase A	DHX9	4.97	1,00E-05	No
Q6PML9	Zinc transporter 9	SLC30A9	4.96	4,00E-04	Yes
A0A0A0MR M9	Nucleolar and coiled-body phosphoprotein 1 (Fragment)	NOLC1	4.82	0.00602	No
P56537	Eukaryotic translation initiation factor 6	EIF6	4.79	1,00E-05	No
P07305	Histone H1.0	H1F0	4.79	3,00E-05	No
P61513	60S ribosomal protein L37a	RPL37A	4.78	0	No
A6PW57	Phosphatidylinositol 4-phosphate 5-kinase type-1 alpha	PIP5K1A	4.77	9,00E-05	No
P50914	60S ribosomal protein L14	RPL14	4.75	1,00E-05	No
P62266	40S ribosomal protein S23	RPS23	4.74	4,00E-05	No
Q6P161	39S ribosomal protein L54, mitochondrial (mL54)	MRPL54	4.68	0.0013	Yes
P62917	60S ribosomal protein L8	RPL8	4.68	0	No
Q13428	Treacle protein	TCOF1	4.68	0.00649	No
P62277	40S ribosomal protein S13	RPS13	4.67	0	No
P05388	60S acidic ribosomal protein P0	RPLP0	4.64	0	No
A4D1E9	GTP-binding protein 10	GTPBP10	4.63	1,00E-04	Yes
Q7L0Y3	tRNA methyltransferase 10 homolog C	TRMT10C	4.63	6.9e-4	Yes
Q8TCC3	39S ribosomal protein L30, mitochondrial (uL30m)	MRPL30	4.62	3.2e-4	Yes
P62753	40S ribosomal protein S6	RPS6	4.62	3,00E-05	No
P62906	60S ribosomal protein L10a	RPL10A	4.61	0	Yes
Q9Y3U8	60S ribosomal protein L36	RPL36	4.6	0	No
Q4U2R6	39S ribosomal protein L51, mitochondrial (mL51)	MRPL51	4.58	0.00179	Yes
Q9Y4F1	FERM, ARHGEF and pleckstrin domain-containing protein 1	FARP1	4.51	8,00E-05	No
Q8WXX5	DnaJ homolog subfamily C member 9	DNAJC9	4.5	4,00E-05	No
P46087	Probable 28S rRNA (cytosine(4447)-C(5))-methyltransferase	NOP2	4.5	1,00E-05	No
Q86UE4	Protein LYRIC	MTDH	4.49	2.7e-4	No
O60832	H/ACA ribonucleoprotein complex subunit DKC1	DKC1	4.48	1,00E-05	No
Q17R31	Putative deoxyribonuclease TATDN3	TATDN3	4.47	0.00237	No
Q4KMP7	TBC1 domain family member 10B	TBC1D10B	4.47	7.1e-4	No

Q8TAE8	Growth arrest and DNA damage-inducible proteins-interacting protein 1	MRPL59	4.45	1,00E-04	Yes
Q02543	60S ribosomal protein L18a	RPL18A	4.42	1,00E-05	No
Q9BQ67	Glutamate-rich WD repeat-containing protein 1	GRWD1	4.39	5,00E-05	No
Q02878	60S ribosomal protein L6	RPL6	4.37	1,00E-05	No
P62424	60S ribosomal protein L7a	RPL7A	4.36	0	No
Q9NVV4	Poly(A) RNA polymerase, mitochondrial	MTPAP	4.36	0.00733	Yes
P18124	60S ribosomal protein L7	RPL7	4.32	0	No
P46778	60S ribosomal protein L21	RPL21	4.31	0	No
P42677	40S ribosomal protein S27	RPS27	4.28	4.1e-4	No
P18621	60S ribosomal protein L17	RPL17	4.28	1,00E-05	No
Q96QR8	Transcriptional activator protein Pur-beta	PURB	4.24	3,00E-05	No
Q9BRJ6	Uncharacterized protein C7orf50	C7orf50	4.23	5,00E-05	No
Q7Z7H8	39S ribosomal protein L10, mitochondrial (uL10m)	MRPL10	4.21	5,00E-05	Yes
Q9BYD3	39S ribosomal protein L4, mitochondrial (uL4m)	MRPL4	4.21	1.2e-4	Yes
P27635	60S ribosomal protein L10	RPL10	4.18	1,00E-05	No
Q13263	Transcription intermediary factor 1-beta	TRIM28	4.18	4.2e-4	No
Q9Y4P3	Transducin beta-like protein 2	TBL2	4.18	0.00192	No
P10412	Histone H1.4	HIST1H1E	4.17	0.00125	No
O00567	Nucleolar protein 56	NOP56	4.17	4,00E-05	No
Q9Y221	60S ribosome subunit biogenesis protein NIP7 homolog	NIP7	4.12	4,00E-05	No
O95819	Mitogen-activated protein kinase kinase kinase kinase 4	MAP4K4	4.09	0.00478	No
Q13084	39S ribosomal protein L28, mitochondrial (bL28m)	MRPL28	4.08	2,00E-05	Yes
P62851	40S ribosomal protein S25	RPS25	4.07	5,00E-05	No
P16403	Histone H1.2	HIST1H1C	4.07	5.2e-4	No
P62891	60S ribosomal protein L39	RPL39	4.06	3,00E-05	No
F8W7C6	60S ribosomal protein L10	RPL10	4.05	2.4e-4	No
Q9UQ80	Proliferation-associated protein 2G4	PA2G4	4.04	4.7e-4	No
P37108	Signal recognition particle 14 kDa protein	SRP14	4.03	6,00E-05	No
Q9BVP2	Guanine nucleotide-binding protein-like 3	GNL3	3.98	0.00493	No
Q9P015	39S ribosomal protein L15, mitochondrial (uL15m)	MRPL15	3.96	2.6e-4	Yes
P62280	40S ribosomal protein S11	RPS11	3.95	5,00E-05	No
Q9Y6G3	39S ribosomal protein L42, mitochondrial (mL42)	MRPL42	3.94	0.00167	Yes
P62829	60S ribosomal protein L23	RPL23	3.94	2,00E-05	No
O75569	Interferon-inducible double-stranded RNA-dependent protein kinase activator A	PRKRA	3.93	1.1e-4	No
Q9H6S0	3'-5' RNA helicase YTHDC2	YTHDC2	3.91	3.1e-4	No
Q5T653	39S ribosomal protein L2, mitochondrial (uL2m)	MRPL2	3.91	0.0052	Yes

Q8IYB3	Serine/arginine repetitive matrix protein 1	SRRM1	3.91	0.001	No
P39023	60S ribosomal protein L3	RPL3	3.9	2,00E-05	No
Q8TDN6	Ribosome biogenesis protein BRX1 homolog	BRIX1	3.89	2.2e-4	No
Q96A35	39S ribosomal protein L24, mitochondrial (uL24m)	MRPL24	3.88	1.2e-4	Yes
P62249	40S ribosomal protein S16	RPS16	3.88	5,00E-05	No
Q6NZI2	Caveolae-associated protein 1	CAVIN1	3.88	0.00391	No
A0A0A0MQ W0	Myelin expression factor 2, isoform CRA_b	MYEF2	3.87	0.00486	No
F8WF56	Lysine-specific demethylase 5C	KDM5C	3.86	8,00E-05	No
P35659	Protein DEK	DEK	3.84	4.4e-4	No
P83731	60S ribosomal protein L24	RPL24	3.82	8,00E-05	No
Q9H7E9	UPF0488 protein C8orf33	C8orf33	3.82	2,00E-04	No
Q16540	39S ribosomal protein L23, mitochondrial (uL23m)	MRPL23	3.8	1,00E-04	Yes
Q9HCM4	Band 4.1-like protein 5	EPB41L5	3.8	4,00E-05	No
P05386	60S acidic ribosomal protein P1	RPLP1	3.79	1,00E-05	No
Q9NVP1	ATP-dependent RNA helicase DDX18	DDX18	3.79	1.5e-4	No
Q8WVM0	Dimethyladenosine transferase 1, mitochondrial	TFB1M	3.79	7.8e-4	Yes
Q7Z2W4	Zinc finger CCCH-type antiviral protein 1	ZC3HAV1	3.79	5,00E-04	No
Q9H7H0	Methyltransferase-like protein 17, mitochondrial	METTL17	3.78	4,00E-05	Yes
Q9BYC9	39S ribosomal protein L20, mitochondrial (bL20m)	MRPL20	3.77	1.7e-4	Yes
Q7Z2W9	39S ribosomal protein L21, mitochondrial (bL21m)	MRPL21	3.74	7.3e-4	Yes
Q8N983	39S ribosomal protein L43, mitochondrial (mL43)	MRPL43	3.71	1.7e-4	Yes
Q9BYD2	39S ribosomal protein L9, mitochondrial (bL9m)	MRPL9	3.71	5,00E-05	Yes
P61247	40S ribosomal protein S3a	RPS3A	3.71	6,00E-05	No
P62241	40S ribosomal protein S8	RPS8	3.68	4,00E-05	No
Q8IXM3	39S ribosomal protein L41, mitochondrial (mL41)	MRPL41	3.67	8,00E-05	Yes
Q9P0M9	39S ribosomal protein L27, mitochondrial (bL27m)	MRPL27	3.66	1.6e-4	Yes
P09001	39S ribosomal protein L3, mitochondrial (uL3m)	MRPL3	3.66	2.4e-4	Yes
Q9P0J6	39S ribosomal protein L36, mitochondrial (bL36m)	MRPL36	3.64	8,00E-05	Yes
Q9H9J2	39S ribosomal protein L44, mitochondrial (mL44)	MRPL44	3.63	3.2e-4	Yes
P35268	60S ribosomal protein L22	RPL22	3.62	3,00E-05	No
Q8N5N7	39S ribosomal protein L50, mitochondrial (mL50)	MRPL50	3.59	0.00119	Yes
P62847	40S ribosomal protein S24	RPS24	3.58	6,00E-05	No
P55769	NHP2-like protein 1	SNU13	3.57	1.7e-4	No
Q86TS9	39S ribosomal protein L52, mitochondrial (mL52)	MRPL52	3.55	0.01268	Yes
P62750	60S ribosomal protein L23a	RPL23A	3.55	4,00E-05	No
Q9NVS2	39S ribosomal protein S18a, mitochondrial (mL66)	MRPL66	3.53	5,00E-05	Yes

P62888	60S ribosomal protein L30	RPL30	3.53	1,00E-05	No
O75394	39S ribosomal protein L33, mitochondrial (bL33m)	MRPL33	3.52	2.2e-4	Yes
Q92522	Histone H1x	H1FX	3.52	0.00169	No
P32969	60S ribosomal protein L9	RPL9; RPL9P7; RPL9P8; RPL9P9	3.49	2,00E-05	No
P49458	Signal recognition particle 9 kDa protein	SRP9	3.49	3.8e-4	No
Q01081	Splicing factor U2AF 35 kDa subunit	U2AF1	3.49	0.00518	No
P18077	60S ribosomal protein L35a	RPL35A	3.42	5,00E-05	Yes
Q6IN84	rRNA methyltransferase 1, mitochondrial	MRM1	3.38	0.00209	Yes
P05455	Lupus La protein	SSB	3.37	1.7e-4	No
Q96EH3	Mitochondrial assembly of ribosomal large subunit protein 1	MALSU1	3.37	0.00152	Yes
Q00577	Transcriptional activator protein Pur-alpha	PURA	3.37	0.00227	No
P05387	60S acidic ribosomal protein P2	RPLP2	3.35	1,00E-05	No
Q8N0Z8	tRNA pseudouridine synthase-like 1	PUSL1	3.35	8.2e-4	Yes
Q9NW13	RNA-binding protein 28	RBM28	3.33	0.00143	No
P42766	60S ribosomal protein L35	RPL35	3.29	5,00E-05	No
P62913	60S ribosomal protein L11	RPL11	3.24	4,00E-05	No
P49590	Probable histidine--tRNA ligase, mitochondrial	HARS2	3.24	0.00152	Yes
Q9NWU5	39S ribosomal protein L22, mitochondrial (uL22m)	MRPL22	3.23	8,00E-05	Yes
P61964	WD repeat-containing protein 5	WDR5	3.18	2.7e-4	No
Q9HCE1	Putative helicase MOV-10	MOV10	3.17	0.00119	No
Q96SB4	SRSF protein kinase 1	SRPK1	3.13	1.3e-4	No
Q9BUJ2	Heterogeneous nuclear ribonucleoprotein U-like protein 1	HNRNPUL1	3.1	4.2e-4	No
P61353	60S ribosomal protein L27	RPL27	3.09	6,00E-05	No
P30050	60S ribosomal protein L12	RPL12	3.06	3,00E-05	No
Q16777	Histone H2A type 2-C	HIST2H2AC	3.05	0.02602	No
P51116	Fragile X mental retardation syndrome-related protein 2	FXR2	3.03	4.1e-4	No
Q99551	Transcription termination factor 1, mitochondrial	MTERF1	3.01	1,00E-04	Yes
Q9NUL7	Probable ATP-dependent RNA helicase DDX28	DDX28	2.97	0.00605	Yes
P17844	Probable ATP-dependent RNA helicase DDX5	DDX5	2.96	0.01003	No
O00411	DNA-directed RNA polymerase, mitochondrial	POLRMT	2.95	0.00117	Yes
P06899	Histone H2B type 1-J	HIST1H2BJ	2.95	0.00712	No
P04406	Glyceraldehyde-3-phosphate dehydrogenase	GAPDH	2.91	0.04232	Yes
P43243	Matrin-3	MATR3	2.91	0.00412	No
P62899	60S ribosomal protein L31	RPL31	2.9	2,00E-04	No

Q969S3	Zinc finger protein 622	ZNF622	2.89	0.00204	No
P61254	60S ribosomal protein L26	RPL26	2.88	8,00E-05	No
Q9Y3B7	39S ribosomal protein L11, mitochondrial (uL11m)	MRPL11	2.85	2,00E-04	Yes
P46781	40S ribosomal protein S9	RPS9	2.84	6.3e-4	No
Q9NX24	H/ACA ribonucleoprotein complex subunit 2	NHP2	2.84	0.01966	No
P27448	MAP/microtubule affinity-regulating kinase 3	MARK3	2.84	0.09247	No
P82914	28S ribosomal protein S15, mitochondrial (uS15m)	MRPS15	2.83	0.00201	Yes
P62873	39S ribosomal protein L28, mitochondrial (bL28m)	GNB1	2.8	0.01661	No
P09874	39S ribosomal protein L3, mitochondrial (uL3m)	PARP1	2.8	0.01867	No
Q9BZE4	Nucleolar GTP-binding protein 1	GTPBP4	2.78	0.00505	No
Q9NRX2	39S ribosomal protein L17, mitochondrial (bL17m)	MRPL17	2.77	8.4e-4	Yes
Q92841	Probable ATP-dependent RNA helicase DDX17	DDX17	2.77	0.00587	No
Q96CM3	Mitochondrial RNA pseudouridine synthase RPUSD4	RPUSD4	2.76	0.00732	Yes
Q9Y2X3	Nucleolar protein 58	NOP58	2.76	0.00524	No
P30153	Serine/threonine-protein phosphatase 2A 65 kDa regulatory subunit A alpha isoform	PPP2R1A	2.74	0.01583	No
P09429	High mobility group protein B1	HMGB1	2.69	0.22933	No
O60524	Nuclear export mediator factor NEMF	NEMF	2.68	0.02602	No
Q15050	Ribosome biogenesis regulatory protein homolog	RRS1	2.68	0.0014	No
Q9GZR7	ATP-dependent RNA helicase DDX24	DDX24	2.66	0.0246	No
O14654	Insulin receptor substrate 4	IRS4	2.63	0.06329	No
Q9BU76	Multiple myeloma tumor-associated protein 2	MMTAG2	2.63	0.0246	No
P16402	Histone H1.3	HIST1H1D	2.62	0.00152	No
Q9Y3I0	tRNA-splicing ligase RtcB homolog	RTCB	2.62	0.05269	No
Q99848	Probable rRNA-processing protein EBP2	EBNA1BP2	2.61	0.00888	No
Q01130	Serine/arginine-rich splicing factor 2	SRSF2	2.61	0.00201	No
P25398	40S ribosomal protein S12	RPS12	2.6	4.6e-4	No
Q96EL3	39S ribosomal protein L53, mitochondrial (mL53)	MRPL53	2.59	0.00328	Yes
Q14137	Ribosome biogenesis protein BOP1	BOP1	2.58	0.00954	No
Q9NYK5	39S ribosomal protein L39, mitochondrial (mL39)	MRPL39	2.55	9,00E-04	Yes
Q7Z2T5	TRMT1-like protein	TRMT1L	2.55	0.04578	No
P46782	40S ribosomal protein S5	RPS5	2.54	0.00199	No
Q71DI3	Histone H3.2	HIST2H3A; HIST2H3C; HIST2H3D	2.54	0.0094	No
Q9UMS4	Pre-mRNA-processing factor 19	PRPF19	2.52	0.15635	No
Q92900	Regulator of nonsense transcripts 1	UPF1	2.52	0.0016	No

Q99459	Cell division cycle 5-like protein	CDC5L	2.5	0.07574	No
P62879	Guanine nucleotide-binding protein G(I)/G(S)/G(T) subunit beta-2	GNB2	2.5	0.00984	No
Q96C36	Pyrroline-5-carboxylate reductase 2	PYCR2	2.5	0.01867	Yes
P62244	40S ribosomal protein S15a	RPS15A	2.48	0.00176	Yes
Q96PK6	RNA-binding protein 14	RBM14	2.48	0.06289	No
P68431	Histone H3.1	HIST1H3A; HIST1H3B; HIST1H3C; HIST1H3D; HIST1H3E; HIST1H3F; HIST1H3G; HIST1H3H; HIST1H3I; HIST1H3J	2.46	0.01886	No
Q6P1L8	39S ribosomal protein L14, mitochondrial (uL14m)	MRPL14	2.44	0.00204	Yes
Q96DV4	39S ribosomal protein L38, mitochondrial (mL38)	MRPL38	2.44	9.1e-4	Yes
Q13523	Serine/threonine-protein kinase PRP4 homolog	PRPF4B	2.44	0.0094	No
Q9BRJ2	39S ribosomal protein L45, mitochondrial (mL45)	MRPL45	2.43	0.00233	Yes
Q01780	Exosome component 10	EXOSC10	2.42	0.10294	No
Q7L2E3	Putative ATP-dependent RNA helicase DHX30	DHX30	2.42	7.4e-4	Yes
O00746	Nucleoside diphosphate kinase, mitochondrial	NME4	2.41	0.00529	Yes
Q15070	Mitochondrial inner membrane protein OXA1L	OXA1L	2.4	0.09668	Yes
Q86Y79	Probable peptidyl-tRNA hydrolase	PTRH1	2.4	0.11124	Yes
P62979	Ubiquitin-40S ribosomal protein S27a	RPS27A	2.4	0.0094	No
P62081	40S ribosomal protein S7	RPS7	2.39	0.00422	No
Q9P2E9	Ribosome-binding protein 1	RRBP1	2.38	0.03112	No
P46777	60S ribosomal protein L5	RPL5	2.37	5.2e-4	No
Q12797	Aspartyl/asparaginyl beta-hydroxylase	ASPH	2.37	0.02182	No
Q9UNX3	60S ribosomal protein L26-like 1	RPL26L1	2.36	0.00447	No
Q9BYD1	39S ribosomal protein L13, mitochondrial (uL13m)	MRPL13	2.35	0.00159	Yes
P62861	40S ribosomal protein S30	FAU	2.35	0.017	No
P46013	Proliferation marker protein Ki-67	MKI67	2.35	0.09668	No
Q9BYC8	39S ribosomal protein L32, mitochondrial (bL32m)	MRPL32	2.33	0.00636	Yes
Q14444	Caprin-1	CAPRIN1	2.28	0.01671	No
Q9H0U6	39S ribosomal protein L18, mitochondrial (uL18m)	MRPL18	2.27	8.8e-4	Yes
Q96CB9	5-methylcytosine rRNA methyltransferase NSUN4	NSUN4	2.27	0.00426	Yes
Q13405	39S ribosomal protein L49, mitochondrial (mL49)	MRPL49	2.24	9.2e-4	Yes

P13804	Electron transfer flavoprotein subunit alpha, mitochondrial	ETFA	2.23	0.06956	Yes
Q9NX58	Cell growth-regulating nucleolar protein	LYAR	2.21	0.017	No
Q9Y305	Acyl-coenzyme A thioesterase 9, mitochondrial	ACOT9	2.19	0.07475	Yes
P63173	60S ribosomal protein L38	RPL38	2.18	0.00296	No
Q9NWT8	Aurora kinase A-interacting protein	AURKAIP1	2.18	0.02823	Yes
O75616	GTPase Era, mitochondrial	ERAL1	2.18	0.03637	Yes
Q9NQ55	Suppressor of SWI4 1 homolog	PPAN	2.18	0.00996	No
Q9Y5B9	FACT complex subunit SPT16	SUPT16H	2.16	0.03291	No
Q5TEC6	Histone H3	HIST2H3PS2	2.16	0.02863	No
Q96GC5	39S ribosomal protein L48, mitochondrial (mL48)	MRPL48	2.13	0.00226	Yes
P12235	ADP/ATP translocase 1	SLC25A4	2.13	0.00564	Yes
Q9NP92	39S ribosomal protein S30, mitochondrial (mL65)	MRPS30	2.12	0.00156	Yes
Q86V81	THO complex subunit 4	ALYREF	2.12	0.03794	No
Q13283	Ras GTPase-activating protein-binding protein 1	G3BP1	2.11	0.02299	No
Q9BQG0	Myb-binding protein 1A	MYBBP1A	2.09	0.06329	No
P19338	Nucleolin	NCL	2.05	0.00117	No
P55209	Nucleosome assembly protein 1-like 1	NAP1L1	2.05	0.0094	No
Q96EY4	Translation machinery-associated protein 16	TMA16	2.05	0.01436	No
G5E9E7	Tight junction protein 1 (Zona occludens 1), isoform CRA_e	TJP1	2.03	0.15732	No
Q96B26	Exosome complex component RRP43	EXOSC8	2.01	0.06036	No
O00571	ATP-dependent RNA helicase DDX3X	DDX3X	1.99	0.14836	No
Q9NPE3	H/ACA ribonucleoprotein complex subunit 3	NOP10	1.99	0.04777	No
P53999	Activated RNA polymerase II transcriptional coactivator p15	SUB1	1.98	0.2105	No
Q7Z7F7	39S ribosomal protein L55, mitochondrial (bL31m)	MRPL55	1.97	0.00352	Yes
P36542	ATP synthase subunit gamma, mitochondrial	ATP5F1C	1.97	0.04302	Yes
Q14684	Ribosomal RNA processing protein 1 homolog B	RRP1B	1.97	0.1874	No
Q9BPX6	Calcium uptake protein 1, mitochondrial	MICU1	1.96	0.11183	Yes
Q6PKG0	La-related protein 1	LARP1	1.96	0.02628	No
P08621	U1 small nuclear ribonucleoprotein 70 kDa	SNRNP70	1.96	0.01524	No
P62273	40S ribosomal protein S29	RPS29	1.95	0.01456	No
P52597	Heterogeneous nuclear ribonucleoprotein F	HNRNPF	1.95	0.01044	No
P23396	40S ribosomal protein S3	RPS3	1.93	0.01202	No
P49406	39S ribosomal protein L19, mitochondrial (bL19m)	MRPL19	1.91	0.00746	Yes
Q14197	Peptidyl-tRNA hydrolase ICT1, mitochondrial	MRPL58	1.91	0.00306	Yes
L0R8F8	MIEF1 upstream open reading frame protein	MIEF1	1.89	0.03754	Yes
Q2NL82	Pre-rRNA-processing protein TSR1 homolog	TSR1	1.88	0.03613	No
P57053	Histone H2B type F-S	H2BFS	1.87	0.00856	No

Q9NY12	H/ACA ribonucleoprotein complex subunit 1	GAR1	1.86	0.01993	No
Q8IVS2	Malonyl-CoA-acyl carrier protein transacylase, mitochondrial	MCAT	1.86	0.11573	Yes
P82675	28S ribosomal protein S5, mitochondrial (uS5m)	MRPS5	1.85	0.02983	Yes
Q9NUD5	Zinc finger CCHC domain-containing protein 3	ZCCHC3	1.85	0.01912	No
Q92499	ATP-dependent RNA helicase DDX1	DDX1	1.82	0.14316	No
Q12906	Interleukin enhancer-binding factor 3	ILF3	1.79	0.0068	No
P13995	Bifunctional methylenetetrahydrofolate dehydrogenase/cyclohydrolase, mitochondrial	MTHFD2	1.78	0.02806	Yes
P17987	T-complex protein 1 subunit alpha	TCP1	1.78	0.0829	No
P09661	U2 small nuclear ribonucleoprotein A'	SNRPA1	1.77	0.02387	No
P0C0S5	Histone H2A.Z	H2AFZ	1.76	0.07151	No
O94813	Slit homolog 2 protein	SLIT2	1.75	0.08389	No
Q12905	Interleukin enhancer-binding factor 2	ILF2	1.73	0.01993	No
P84243	Histone H3.3	H3F3A; H3F3B	1.72	0.17056	No
Q96AG4	Leucine-rich repeat-containing protein 59	LRRC59	1.72	0.09479	No
Q96ME7	Zinc finger protein 512	ZNF512	1.72	0.1744	No
P82663	28S ribosomal protein S25, mitochondrial (mS25)	MRPS25	1.71	0.03263	Yes
O00541	Pescadillo homolog	PES1	1.71	0.11125	No
Q969P6	DNA topoisomerase I, mitochondrial	TOP1MT	1.7	0.06237	Yes
P32189	Glycerol kinase	GK	1.69	0.08993	Yes
Q9HC36	rRNA methyltransferase 3, mitochondrial	MRM3	1.69	0.02771	Yes
Q9NQT4	Exosome complex component RRP46	EXOSC5	1.68	0.07463	No
Q3MHD2	Protein LSM12 homolog	LSM12	1.68	0.02657	No
Q92974	Rho guanine nucleotide exchange factor 2	ARHGEF2	1.66	0.28274	No
P48444	Coatomer subunit delta	ARCN1	1.64	0.06459	No
Q12849	G-rich sequence factor 1	GRSF1	1.64	0.01203	Yes
Q8IY81	pre-rRNA processing protein FTSJ3	FTSJ3	1.64	0.23346	No
Q9BYD6	39S ribosomal protein L1, mitochondrial (uL1m)	MRPL1	1.63	0.01055	Yes
P62805	Histone H4	HIST1H4A; HIST1H4B; HIST1H4C; HIST1H4D; HIST1H4E; HIST1H4F; HIST1H4H; HIST1H4I; HIST1H4J;	1.59	0.03904	No

		HIST1H4K; HIST1H4L; HIST2H4A; HIST2H4B; HIST4H4			
Q9H2W6	39S ribosomal protein L46, mitochondrial (mL46)	MRPL46	1.58	0.00857	Yes
B4DLN1	cDNA FLJ60124, highly similar to Mitochondrial dicarboxylate carrier		1.58	0.01309	No
Q9GZR2	RNA exonuclease 4	REXO4	1.58	0.11646	No
P46459	Vesicle-fusing ATPase	NSF	1.56	0.08086	No
Q8NHQ9	ATP-dependent RNA helicase DDX55	DDX55	1.54	0.16441	No
Q9NZ01	Very-long-chain enoyl-CoA reductase	TECR	1.53	0.12242	No
P04844	Dolichyl-diphosphooligosaccharide--protein glycosyltransferase subunit 2	RPN2	1.52	0.07333	No
P0C0S8	Histone H2A type 1	HIST1H2AG; HIST1H2AI; HIST1H2AK; HIST1H2AL; HIST1H2AM	1.52	0.04878	No
P78362	SRSF protein kinase 2	SRPK2	1.52	0.19324	No
H0Y2W2	ATPase family AAA domain-containing protein 3A (Fragment)	ATAD3A	1.51	0.06821	Yes
O75494	Serine/arginine-rich splicing factor 10	SRSF10	1.51	0.02111	No
Q9NQ50	39S ribosomal protein L40, mitochondrial (mL40)	MRPL40	1.5	0.01524	Yes
Q14974	Importin subunit beta-1	KPNB1	1.5	0.08562	No
P60866	40S ribosomal protein S20	RPS20	1.49	0.03291	No
P46783	40S ribosomal protein S10	RPS10	1.47	0.03219	No
Q8N183	NADH dehydrogenase [ubiquinone] 1 alpha subcomplex assembly factor 2	NDUFAF2	1.47	0.07463	Yes
Q96QE5	Transcription elongation factor, mitochondrial	TEFM	1.47	0.16695	Yes
P39019	40S ribosomal protein S19	RPS19	1.46	0.03295	No
P36956	Sterol regulatory element-binding protein 1	SREBF1	1.46	0.10285	No
Q00059	Transcription factor A, mitochondrial	TFAM	1.46	0.06397	Yes
Q9H3N1	Thioredoxin-related transmembrane protein 1	TMX1	1.45	0.19431	No
Q7LGC8	Carbohydrate sulfotransferase 3	CHST3	1.44	0.10424	No
Q9UN86	Ras GTPase-activating protein-binding protein 2	G3BP2	1.44	0.09192	No
P20290	Transcription factor BTF3	BTF3	1.44	0.32642	No
Q9NVI7	ATPase family AAA domain-containing protein 3A	ATAD3A	1.43	0.09668	Yes
Q9BW72	HIG1 domain family member 2A, mitochondrial	HIGD2A	1.43	0.20199	Yes
Q9Y6M1	Insulin-like growth factor 2 mRNA-binding protein 2	IGF2BP2	1.43	0.01088	No
Q07955	Serine/arginine-rich splicing factor 1	SRSF1	1.43	0.0176	No

Q86UQ4	ATP-binding cassette sub-family A member 13	ABCA13	1.42	0.2781	Yes
Q9UKS6	Protein kinase C and casein kinase substrate in neurons protein 3	PACSIN3	1.42	0.24966	No
P40939	Trifunctional enzyme subunit alpha, mitochondrial	HADHA	1.42	0.02969	Yes
Q15717	ELAV-like protein 1	ELAVL1	1.41	0.10036	No
Q9H6F5	Coiled-coil domain-containing protein 86	CCDC86	1.38	0.21347	No
O95363	Phenylalanine--tRNA ligase, mitochondrial	FARS2	1.38	0.1744	Yes
Q14692	Ribosome biogenesis protein BMS1 homolog	BMS1	1.37	0.18828	No
P21741	Midkine	MDK	1.36	0.07631	No
O14617	AP-3 complex subunit delta-1	AP3D1	1.35	0.19754	No
P05783	Keratin, type I cytoskeletal 18	KRT18	1.35	0.06118	No
Q13247	Serine/arginine-rich splicing factor 6	SRSF6	1.35	0.11408	No
Q96A33	Coiled-coil domain-containing protein 47	CCDC47	1.32	0.31752	No
Q00839	Heterogeneous nuclear ribonucleoprotein U	HNRNPU	1.32	0.02387	No
P47985	Cytochrome b-c1 complex subunit Rieske, mitochondrial	UQCRFS1	1.29	0.09301	Yes
Q15287	RNA-binding protein with serine-rich domain 1	RNPS1	1.29	0.14756	No
Q96CW1	AP-2 complex subunit mu	AP2M1	1.28	0.17426	No
Q5T280	Putative methyltransferase C9orf114	SPOUT1	1.27	0.10638	No
O60783	28S ribosomal protein S14, mitochondrial (uS14m)	MRPS14	1.26	0.33705	Yes
P56270	Myc-associated zinc finger protein	MAZ	1.26	0.22933	No
Q71UM5	40S ribosomal protein S27-like	RPS27L	1.25	0.21943	No
P53701	Cytochrome c-type heme lyase	HCCS	1.25	0.34755	Yes
Q9NQT5	Exosome complex component RRP40	EXOSC3	1.25	0.27634	No
P16615	Sarcoplasmic/endoplasmic reticulum calcium ATPase 2	ATP2A2	1.25	0.41645	No
P05198	Eukaryotic translation initiation factor 2 subunit 1	EIF2S1	1.23	0.16163	No
P09651	Heterogeneous nuclear ribonucleoprotein A1	HNRNPA1	1.23	0.08251	No
Q99714	3-hydroxyacyl-CoA dehydrogenase type-2	HSD17B10	1.2	0.06564	Yes
P62834	Ras-related protein Rap-1A	RAP1A	1.2	0.16526	No
Q13151	Heterogeneous nuclear ribonucleoprotein A0	HNRNPA0	1.19	0.27729	No
Q6P087	Mitochondrial mRNA pseudouridine synthase RPUSD3	RPUSD3	1.19	0.32731	Yes
P60468	Protein transport protein Sec61 subunit beta	SEC61B	1.19	0.19251	No
P63244	Receptor of activated protein C kinase 1	RACK1	1.19	0.15794	No
Q9Y2R5	28S ribosomal protein S17, mitochondrial (uS17m)	MRPS17	1.16	0.09184	Yes
Q08J23	tRNA (cytosine(34)-C(5))-methyltransferase	NSUN2	1.16	0.30528	No
Q7L576	Cytoplasmic FMR1-interacting protein 1	CYFIP1	1.15	0.32126	No
O00422	Histone deacetylase complex subunit SAP18	SAP18	1.12	0.11629	No
Q13765	Nascent polypeptide-associated complex subunit alpha	NACA	1.12	0.37144	No
O00425	Insulin-like growth factor 2 mRNA-binding protein 3	IGF2BP3	1.1	0.14782	No

P15954	Cytochrome c oxidase subunit 7C, mitochondrial	COX7C	1.09	0.26179	Yes
P78406	mRNA export factor	RAE1	1.09	0.25396	No
O00139	Kinesin-like protein KIF2A	KIF2A	1.08	0.2928	No
Q96GQ7	Probable ATP-dependent RNA helicase DDX27	DDX27	1.08	0.28233	No
Q9Y3B2	Exosome complex component CSL4	EXOSC1	1.07	0.41606	No
Q5RKV6	Exosome complex component MTR3	EXOSC6	1.06	0.49276	No
Q14318	Peptidyl-prolyl cis-trans isomerase FKBP8	FKBP8	1.05	0.27548	Yes
O76094	Signal recognition particle subunit SRP72	SRP72	1.05	0.39705	No
Q9BYG3	MKI67 FHA domain-interacting nucleolar phosphoprotein	NIFK	1.04	0.30897	No
P00387	NADH-cytochrome b5 reductase 3	CYB5R3	1.04	0.32108	Yes
P82650	28S ribosomal protein S22, mitochondrial (mS22)	MRPS22	1.03	0.13682	Yes
Q71RC2	La-related protein 4	LARP4	1.03	0.28122	No
P08865	40S ribosomal protein SA	RPSA	1.02	0.17605	No
Q13435	Splicing factor 3B subunit 2	SF3B2	1.02	0.3132	No
P35613	Basigin	BSG	1.01	0.46228	No
Q9BTU6	Phosphatidylinositol 4-kinase type 2-alpha	PI4K2A	0.99	0.2806	No
Q13310	Polyadenylate-binding protein 4	PABPC4	0.98	0.09434	No
P24752	Acetyl-CoA acetyltransferase, mitochondrial	ACAT1	0.97	0.48369	Yes
Q15024	Exosome complex component RRP42	EXOSC7	0.96	0.37636	No
P25705	ATP synthase subunit alpha, mitochondrial	ATP5F1A	0.95	0.17901	Yes
P49756	RNA-binding protein 25	RBM25	0.95	0.42069	No
Q9Y3D3	28S ribosomal protein S16, mitochondrial (bS16m)	MRPS16	0.94	0.18601	Yes
P82930	28S ribosomal protein S34, mitochondrial (mS34)	MRPS34	0.94	0.21142	Yes
Q8WXI9	Transcriptional repressor p66-beta	GATAD2B	0.93	0.57506	No
Q6P2E9	Enhancer of mRNA-decapping protein 4	EDC4	0.92	0.22933	No
P10809	60 kDa heat shock protein, mitochondrial	HSPD1	0.91	0.14025	Yes
P12074	Cytochrome c oxidase subunit 6A1, mitochondrial	COX6A1	0.9	0.33259	Yes
Q9Y3C1	Nucleolar protein 16	NOP16	0.9	0.24193	No
A8MQB8	Synaptic functional regulator FMR1	FMR1	0.89	0.23155	No
Q14498	RNA-binding protein 39	RBM39	0.88	0.48992	No
O75683	Surfeit locus protein 6	SURF6	0.88	0.42602	No
Q13868	Exosome complex component RRP4	EXOSC2	0.87	0.60492	No
Q09666	Neuroblast differentiation-associated protein AHNAK	AHNAK	0.87	0.52417	No
Q9NWT1	p21-activated protein kinase-interacting protein 1	PAK1IP1	0.86	0.41628	No
O75607	Nucleoplasmin-3	NPM3	0.85	0.40038	No
Q9Y3E5	Peptidyl-tRNA hydrolase 2, mitochondrial	PTRH2	0.84	0.53931	Yes
Q8N726	Tumor suppressor ARF	CDKN2A	0.84	0.39988	No

Q9NSD9	Phenylalanine--tRNA ligase beta subunit	FARSB	0.83	0.47927	No
Q9BYN8	28S ribosomal protein S26, mitochondrial (mS26)	MRPS26	0.82	0.25959	Yes
Q9Y5J1	U3 small nucleolar RNA-associated protein 18 homolog	UTP18	0.82	0.39689	No
O43660	Pleiotropic regulator 1	PLRG1	0.81	0.43747	No
P61927	60S ribosomal protein L37	RPL37	0.8	0.2943	No
O60716	Catenin delta-1	CTNND1	0.8	0.49298	No
Q9Y2P8	RNA 3'-terminal phosphate cyclase-like protein	RCL1	0.8	0.36212	No
P52815	39S ribosomal protein L12, mitochondrial (bL12m)	MRPL12	0.79	0.15098	Yes
Q13895	Bystin	BYSL	0.79	0.42069	No
P13861	cAMP-dependent protein kinase type II-alpha regulatory subunit	PRKAR2A	0.79	0.39495	No
P62995	Transformer-2 protein homolog beta	TRA2B	0.78	0.30408	No
Q9H5Q4	Dimethyladenosine transferase 2, mitochondrial	TFB2M	0.76	0.27667	Yes
Q8NC51	Plasminogen activator inhibitor 1 RNA-binding protein	SERBP1	0.75	0.44248	No
A0A096LPI6	Uncharacterized protein (Fragment)		0.75	0.41943	No
Q16836	Hydroxyacyl-coenzyme A dehydrogenase, mitochondrial	HADH	0.74	0.52981	Yes
P61586	Transforming protein RhoA	RHOA	0.74	0.2694	No
Q9NQC3	Reticulon-4	RTN4	0.73	0.6097	No
O43491	Band 4.1-like protein 2	EPB41L2	0.72	0.39554	No
Q6IAA8	Ragulator complex protein LAMTOR1	LAMTOR1	0.72	0.3949	No
P35250	Replication factor C subunit 2	RFC2	0.72	0.36982	No
P35249	Replication factor C subunit 4	RFC4	0.71	0.36954	No
O15126	Secretory carrier-associated membrane protein 1	SCAMP1	0.71	0.36705	No
Q13838	Spliceosome RNA helicase DDX39B	DDX39B	0.71	0.39063	No
O00116	Alkyldihydroxyacetonephosphate synthase, peroxisomal	AGPS	0.7	0.4951	No
P62857	40S ribosomal protein S28	RPS28	0.69	0.30238	No
Q9NRW3	DNA dC->dU-editing enzyme APOBEC-3C	APOBEC3C	0.69	0.64227	No
Q9H0A0	RNA cytidine acetyltransferase	NAT10	0.69	0.44806	No
O95292	Vesicle-associated membrane protein-associated protein B/C	VAPB	0.69	0.54808	No
Q92667	A-kinase anchor protein 1, mitochondrial	AKAP1	0.68	0.41009	Yes
Q9H0U3	Magnesium transporter protein 1	MAGT1	0.68	0.50602	No
Q9UNQ2	Probable dimethyladenosine transferase	DIMT1	0.67	0.55269	No
Q96JJ7	Protein disulfide-isomerase TMX3	TMX3	0.67	0.51166	No
P62491	Ras-related protein Rab-11A	RAB11A	0.67	0.68016	No
Q15233	Non-POU domain-containing octamer-binding protein	NONO	0.66	0.55724	No
Q9Y606	tRNA pseudouridine synthase A	PUS1	0.66	0.42774	Yes
S4R3H4	Apoptotic chromatin condensation inducer in the nucleus	ACIN1	0.65	0.49298	No
Q9NPD3	Exosome complex component RRP41	EXOSC4	0.65	0.46649	No

P36776	Lon protease homolog, mitochondrial	LONP1	0.65	0.52325	Yes
P49959	Double-strand break repair protein MRE11	MRE11	0.64	0.42331	No
Q9H0U4	Ras-related protein Rab-1B	RAB1B	0.64	0.43047	No
A0A024R4E5	High density lipoprotein binding protein (Vigilin), isoform CRA_a	HDLBP	0.63	0.75207	No
Q9H3G5	Probable serine carboxypeptidase CPVL	CPVL	0.63	0.4776	No
P11171	Protein 4.1	EPB41	0.63	0.43365	No
P12532	Creatine kinase U-type, mitochondrial	CKMT1A; CKMT1B	0.62	0.53866	Yes
Q99547	M-phase phosphoprotein 6	MPHOSPH6	0.62	0.59072	No
O75934	Pre-mRNA-splicing factor SPF27	BCAS2	0.62	0.67873000000000006	No
P52292	Importin subunit alpha-1	KPNA2	0.61	0.53238	No
P83111	Serine beta-lactamase-like protein LACTB, mitochondrial	LACTB	0.61	0.4956	Yes
O15031	Plexin-B2	PLXNB2	0.6	0.58436	No
Q5VTL8	Pre-mRNA-splicing factor 38B	PRPF38B	0.6	0.5565	No
Q99653	Calcineurin B homologous protein 1	CHP1	0.59	0.44396	No
Q08170	Serine/arginine-rich splicing factor 4	SRSF4	0.59	0.48244	No
P53985	Monocarboxylate transporter 1	SLC16A1	0.58	0.59111	Yes
Q14160	Protein scribble homolog	SCRIB	0.58	0.56329	No
Q8NBX0	Saccharopine dehydrogenase-like oxidoreductase	SCCPDH	0.58	0.62595	Yes
P48047	ATP synthase subunit O, mitochondrial	ATP5O	0.55	0.43365	Yes
Q8IYU8	Calcium uptake protein 2, mitochondrial	MICU2	0.53	0.63562	Yes
Q8WVX9	Fatty acyl-CoA reductase 1	FAR1	0.53	0.62676	No
O94887	FERM, ARHGEF and pleckstrin domain-containing protein 2	FARP2	0.53	0.5525	No
A0A0A6YYJ8	Putative RNA-binding protein Luc7-like 2	LUC7L2	0.53	0.54345	No
Q9UI09	NADH dehydrogenase [ubiquinone] 1 alpha subcomplex subunit 12	NDUFA12	0.52	0.45973	Yes
Q13610	Periodic tryptophan protein 1 homolog	PWP1	0.52	0.60285	No
P26599	Polypyrimidine tract-binding protein 1	PTBP1	0.51	0.62573	No
P11940	Polyadenylate-binding protein 1	PABPC1	0.5	0.34433	No
Q6PK04	Coiled-coil domain-containing protein 137	CCDC137	0.49	0.59006	No
P19367	Hexokinase-1	HK1	0.49	0.65335	Yes
P15151	Poliovirus receptor	PVR	0.48	0.66209	No
Q9Y285	Phenylalanine--tRNA ligase alpha subunit	FARSA	0.47	0.67595	No
P61026	Ras-related protein Rab-10	RAB10	0.47	0.7083	No
Q01813	ATP-dependent 6-phosphofructokinase, platelet type	PFKP	0.45	0.68554	No

P33527	Multidrug resistance-associated protein 1	ABCC1	0.45	0.71579	No
Q9BTD8	RNA-binding protein 42	RBM42	0.45	0.53866	No
Q6DKI1	60S ribosomal protein L7-like 1	RPL7L1	0.44	0.66258	No
P51991	Heterogeneous nuclear ribonucleoprotein A3	HNRNPA3	0.44	0.54345	No
Q16629	Serine/arginine-rich splicing factor 7	SRSF7	0.44	0.65368	No
P02786	Transferrin receptor protein 1	TFRC	0.44	0.73098	No
Q8WWM7	Ataxin-2-like protein	ATXN2L	0.43	0.75001	No
P05023	Sodium/potassium-transporting ATPase subunit alpha-1	ATP1A1	0.43	0.63793	No
P26368	Splicing factor U2AF 65 kDa subunit	U2AF2	0.43	0.62595	No
O95782	AP-2 complex subunit alpha-1	AP2A1	0.42	0.71380999999999994	No
P50402	Emerin	EMD	0.41	0.75105	No
O95470	Sphingosine-1-phosphate lyase 1	SGPL1	0.41	0.75105	No
Q9NPL8	Complex I assembly factor TIMMDC1, mitochondrial	TIMMDC1	0.4	0.53866	Yes
Q96EY1	DnaJ homolog subfamily A member 3, mitochondrial	DNAJA3	0.4	0.55381	Yes
P31942	Heterogeneous nuclear ribonucleoprotein H3	HNRNPH3	0.4	0.55724	No
Q92692	Nectin-2	NECTIN2	0.4	0.70166	No
Q9HD45	Transmembrane 9 superfamily member 3	TM9SF3	0.4	0.66496	No
P82921	28S ribosomal protein S21, mitochondrial (bS21m)	MRPS21	0.39	0.60626	Yes
P63220	40S ribosomal protein S21	RPS21	0.39	0.60747	No
Q9HDC5	Junctophilin-1	JPH1	0.39	0.72417	No
Q9BVV7	Mitochondrial import inner membrane translocase subunit Tim21	TIMM21	0.39	0.60606	Yes
O43809	Cleavage and polyadenylation specificity factor subunit 5	NUDT21	0.38	0.54546	No
P50897	Palmitoyl-protein thioesterase 1	PPT1	0.38	0.76458	No
Q13724	Mannosyl-oligosaccharide glucosidase	MOGS	0.37	0.73603	No
P04843	Dolichyl-diphosphooligosaccharide--protein glycosyltransferase subunit 1	RPN1	0.36	0.65872	No
Q9Y224	RNA transcription, translation and transport factor protein	RTRAF	0.36	0.71705	No
Q9C0E8	Endoplasmic reticulum junction formation protein lunapark	LNPK	0.35	0.70793	No
Q9BSJ8	Extended synaptotagmin-1	ESYT1	0.35	0.81264	No
P46940	Ras GTPase-activating-like protein IQGAP1	IQGAP1	0.35	0.80944	No
Q7Z478	ATP-dependent RNA helicase DHX29	DHX29	0.34	0.85986	No
Q13501	Sequestosome-1	SQSTM1	0.34	0.77128	No
P05067	Amyloid-beta A4 protein	APP	0.33	0.62573	No
P56134	ATP synthase subunit f, mitochondrial	ATP5J2	0.33	0.78326	Yes
Q9Y251	Heparanase	HPSE	0.33	0.80082	No
Q96B49	Mitochondrial import receptor subunit TOM6 homolog	TOMM6	0.33	0.79304	Yes
Q7Z5H3	Rho GTPase-activating protein 22	ARHGAP22	0.32	0.85146	No

Q3ZCM7	Tubulin beta-8 chain	TUBB8	0.32	0.80041	No
P27348	14-3-3 protein theta	YWHAQ	0.29	0.8246	No
Q9BSD7	Cancer-related nucleoside-triphosphatase	NTPCR	0.29	0.79374	No
Q9P258	Protein RCC2	RCC2	0.29	0.87296	No
P82933	28S ribosomal protein S9, mitochondrial (uS9m)	MRPS9	0.28	0.66209	Yes
O60841	Eukaryotic translation initiation factor 5B	EIF5B	0.28	0.85146	No
H7BXI1	Extended synaptotagmin-2 (Fragment)	ESYT2	0.28	0.77541	No
O14929	Histone acetyltransferase type B catalytic subunit	HAT1	0.28	0.76646	No
Q7Z434	Mitochondrial antiviral-signaling protein	MAVS	0.27	0.80082	Yes
Q13242	Serine/arginine-rich splicing factor 9	SRSF9	0.27	0.68993	No
Q96NB2	Sideroflexin-2	SFXN2	0.27	0.78326	Yes
P08670	Vimentin	VIM	0.27	0.70119	No
Q96RR1	Twinkle protein, mitochondrial	TWNK	0.26	0.87954	Yes
O00165	HCLS1-associated protein X-1	HAX1	0.25	0.78718	No
Q9UHD8	Septin-9	09-Sep	0.25	0.8317	No
O75976	Carboxypeptidase D	CPD	0.24	0.83991	No
Q96GA3	Protein LTV1 homolog	LTV1	0.24	0.81817	No
P20700	Lamin-B1	LMNB1	0.23	0.89249	No
Q9Y5J9	Mitochondrial import inner membrane translocase subunit Tim8 B	TIMM8B	0.23	0.75499	Yes
P43307	Translocon-associated protein subunit alpha	SSR1	0.23	0.77809	No
P13639	Elongation factor 2	EEF2	0.22	0.75693	No
P40227	T-complex protein 1 subunit zeta	CCT6A	0.22	0.87097	No
Q8IZP0	Abl interactor 1	ABI1	0.21	0.86638	No
Q0VGL1	Ragulator complex protein LAMTOR4	LAMTOR4	0.21	0.76702	No
Q9UKE5	TRAF2 and NCK-interacting protein kinase	TNIK	0.2	0.85986	No
Q10713	Mitochondrial-processing peptidase subunit alpha	PMPCA	0.19	0.91055	Yes
Q9BVI4	Nucleolar complex protein 4 homolog	NOC4L	0.19	0.85422	No
Q8WTT2	Nucleolar complex protein 3 homolog	NOC3L	0.18	0.87879	No
Q9UBS4	DnaJ homolog subfamily B member 11	DNAJB11	0.17	0.87174	No
P63000	Ras-related C3 botulinum toxin substrate 1	RAC1	0.17	0.87227	No
P06280	Alpha-galactosidase A	GLA	0.15	0.8591	No
Q6IAN0	Dehydrogenase/reductase SDR family member 7B	DHRS7B	0.15	0.90029	Yes
Q9BUR5	MICOS complex subunit MIC26	APOO	0.15	0.90779	Yes
Q56VL3	OCIA domain-containing protein 2	OCIAD2	0.15	0.90005	Yes
Q9H4G0	Band 4.1-like protein 1	EPB41L1	0.14	0.89171	No
P41214	Eukaryotic translation initiation factor 2D	EIF2D	0.14	0.92673	No
Q9BXW7	Haloacid dehalogenase-like hydrolase domain-containing 5	HDHD5	0.14	0.91055	Yes

O14646	Chromodomain-helicase-DNA-binding protein 1	CHD1	0.13	0.8708	No
P55084	Trifunctional enzyme subunit beta, mitochondrial	HADHB	0.13	0.82874	Yes
Q9UBU9	Nuclear RNA export factor 1	NXF1	0.12	0.87662	No
Q9Y3T9	Nucleolar complex protein 2 homolog	NOC2L	0.12	0.9267	No
P04181	Ornithine aminotransferase, mitochondrial	OAT	0.12	0.93073	Yes
Q14694	Ubiquitin carboxyl-terminal hydrolase 10	USP10	0.12	0.92202	No
Q9NYF8	Bcl-2-associated transcription factor 1	BCLAF1	0.11	0.86443	No
P04908	Histone H2A type 1-B/E	HIST1H2AB; HIST1H2AE	0.11	0.91475	No
Q9NTJ5	Phosphatidylinositide phosphatase SAC1	SACM1L	0.11	0.93121	No
Q99832	T-complex protein 1 subunit eta	CCT7	0.11	0.93073	Yes
P18031	Tyrosine-protein phosphatase non-receptor type 1	PTPN1	0.11	0.9277	No
P38919	Eukaryotic initiation factor 4A-III	EIF4A3	0.1	0.90005	No
O94925	Glutaminase kidney isoform, mitochondrial	GLS	0.1	0.90446	Yes
P08754	Guanine nucleotide-binding protein G(k) subunit alpha	GNAI3	0.09	0.94834	No
P98173	Protein FAM3A	FAM3A	0.09	0.91968	No
Q9UNX4	WD repeat-containing protein 3	WDR3	0.09	0.93373	No
Q9Y2R9	28S ribosomal protein S7, mitochondrial (uS7m)	MRPS7	0.08	0.91055	Yes
P51398	28S ribosomal protein S29, mitochondrial (mS29)	DAP3	0.07	0.91055	Yes
P53597	Succinate--CoA ligase [ADP/GDP-forming] subunit alpha, mitochondrial	SUCLG1	0.07	0.95407	Yes
P67809	Nuclease-sensitive element-binding protein 1	YBX1	0.06	0.9277	No
P48735	Isocitrate dehydrogenase [NADP], mitochondrial	IDH2	0.05	0.97153	Yes
Q8WUH6	Transmembrane protein 263	TMEM263	0.05	0.96911	No
O00217	NADH dehydrogenase [ubiquinone] iron-sulfur protein 8, mitochondrial	NDUFS8	0.04	0.97336	Yes
Q96HS1	Serine/threonine-protein phosphatase PGAM5, mitochondrial	PGAM5	0.04	0.94364	Yes
Q15526	Surfeit locus protein 1	SURF1	0.04	0.96751	Yes
P38117	Electron transfer flavoprotein subunit beta	ETFB	0.03	0.98102	Yes
Q14108	Lysosome membrane protein 2	SCARB2	0.03	0.98259	No
O95900	Mitochondrial mRNA pseudouridine synthase TRUB2	TRUB2	0.03	0.97439	Yes
P23634	Plasma membrane calcium-transporting ATPase 4	ATP2B4	0.03	0.97336	No
P84103	Serine/arginine-rich splicing factor 3	SRSF3	0.03	0.97028	No
P43686	26S proteasome regulatory subunit 6B	PSMC4	0.02	0.98242	No
O60762	Dolichol-phosphate mannosyltransferase subunit 1	DPM1	0.02	0.98319	No
P38646	Stress-70 protein, mitochondrial	HSPA9	0.02	0.97911	Yes
Q9NVH1	DnaJ homolog subfamily C member 11	DNAJC11	0.01	0.98764	Yes
Q9NZI8	Insulin-like growth factor 2 mRNA-binding protein 1	IGF2BP1	0.01	0.9836	No
P23588	Eukaryotic translation initiation factor 4B	EIF4B	-8.5	3.6e-4	No

O75390	Citrate synthase, mitochondrial	CS	-7.73	0.00357	Yes
Q9NR28	Diablo homolog, mitochondrial	DIABLO	-7.32	4,00E-05	Yes
Q13464	Rho-associated protein kinase 1	ROCK1	-6.91	2,00E-04	No
Q08378	Golgin subfamily A member 3	GOLGA3	-6.88	9,00E-05	No
P52732	Kinesin-like protein KIF11	KIF11	-6.79	0.05348	No
P46821	Microtubule-associated protein 1B	MAP1B	-6.6	4,00E-05	No
Q9P2R3	Rabankyrin-5	ANKFY1	-6.57	1.9e-4	No
P17066	Heat shock 70 kDa protein 6	HSPA6	-6.44	0	No
Q9NQ92	Coordinator of PRMT5 and differentiation stimulator	COPRS	-6.03	2,00E-05	No
O14639	Actin-binding LIM protein 1	ABLIM1	-5.92	6.2e-4	No
Q9P1Y5	Calmodulin-regulated spectrin-associated protein 3	CAMSAP3	-5.69	1,00E-05	No
O75369	Filamin-B	FLNB	-5.69	0	No
Q53FZ2	Acyl-coenzyme A synthetase ACSM3, mitochondrial	ACSM3	-5.65	1.4e-4	Yes
O43852	Calumenin	CALU	-5.65	1,00E-05	No
P21333	Filamin-A	FLNA	-5.59	1.7e-4	No
Q86YM7	Homer protein homolog 1	HOMER1	-5.57	2,00E-05	No
Q14766	Latent-transforming growth factor beta-binding protein 1	LTBP1	-5.54	4.9e-4	No
Q60FE5	Filamin-A	FLNA	-5.52	1.1e-4	No
O60568	Procollagen-lysine,2-oxoglutarate 5-dioxygenase 3	PLOD3	-5.49	2,00E-05	No
O43488	Aflatoxin B1 aldehyde reductase member 2	AKR7A2	-5.35	2.7e-4	Yes
Q9BQA1	Methylosome protein 50	WDR77	-5.21	0.01914	No
Q3V6T2	Girdin	CCDC88A	-5.16	0.00233	No
O14744	Protein arginine N-methyltransferase 5	PRMT5	-5.13	0.02229	No
Q15750	TGF-beta-activated kinase 1 and MAP3K7-binding protein 1	TAB1	-5.1	6.1e-4	No
Q15057	Arf-GAP with coiled-coil, ANK repeat and PH domain-containing protein 2	ACAP2	-5.09	1.7e-4	No
Q16740	ATP-dependent Clp protease proteolytic subunit, mitochondrial	CLPP	-5.08	1.2e-4	Yes
Q9P219	Protein Daple	CCDC88C	-5.07	0.00805	No
Q9Y262	Eukaryotic translation initiation factor 3 subunit L	EIF3L	-5.02	1.7e-4	No
Q99417	c-Myc-binding protein	MYCBP	-4.9	0.00826	No
Q9NSB8	Homer protein homolog 2	HOMER2	-4.9	1.3e-4	No
P42704	Leucine-rich PPR motif-containing protein, mitochondrial	LRPPRC	-4.9	3.5e-4	Yes
Q13813	Spectrin alpha chain, non-erythrocytic 1	SPTAN1	-4.9	1.2e-4	No
P09622	Dihydrolipoyl dehydrogenase, mitochondrial	DLD	-4.88	1.7e-4	Yes
Q01082	Spectrin beta chain, non-erythrocytic 1	SPTBN1	-4.84	1.7e-4	No
P55884	Eukaryotic translation initiation factor 3 subunit B	EIF3B	-4.83	8.2e-4	No
Q8NHP8	Putative phospholipase B-like 2	PLBD2	-4.82	1.8e-4	No
Q9Y6Y0	Influenza virus NS1A-binding protein	IVNS1ABP	-4.8	3.1e-4	No

O75821	Eukaryotic translation initiation factor 3 subunit G	EIF3G	-4.73	0	No
P30041	Peroxiredoxin-6	PRDX6	-4.73	4,00E-05	Yes
O43318	Mitogen-activated protein kinase kinase kinase 7	MAP3K7	-4.69	6.4e-4	No
P07237	Protein disulfide-isomerase	P4HB	-4.67	3.8e-4	Yes
P51649	Succinate-semialdehyde dehydrogenase, mitochondrial	ALDH5A1	-4.67	7.8e-4	Yes
A0A0D9SF54	Spectrin alpha chain, non-erythrocytic 1	SPTAN1	-4.6	6.2e-4	No
Q9GZT3	SRA stem-loop-interacting RNA-binding protein, mitochondrial	SLIRP	-4.59	5,00E-04	Yes
P82909	28S ribosomal protein S36, mitochondrial	MRPS36	-4.57	2,00E-05	Yes
Q13561	Dynactin subunit 2	DCTN2	-4.55	5,00E-05	No
P17931	Galectin-3	LGALS3	-4.52	2,00E-05	No
P29144	Tripeptidyl-peptidase 2	TPP2	-4.49	1.7e-4	No
Q86TV6	Tetratricopeptide repeat protein 7B	TTC7B	-4.44	5.7e-4	No
P22061	Protein-L-isoaspartate(D-aspartate) O-methyltransferase	PCMT1	-4.43	4.9e-4	No
P54105	Methylosome subunit pICln	CLNS1A	-4.4	9.4e-4	No
Q9H2U2	Inorganic pyrophosphatase 2, mitochondrial	PPA2	-4.39	3.7e-4	Yes
P16152	Carbonyl reductase [NADPH] 1	CBR1	-4.36	5.1e-4	No
Q9NYJ8	TGF-beta-activated kinase 1 and MAP3K7-binding protein 2	TAB2	-4.35	0.00499	No
O00303	Eukaryotic translation initiation factor 3 subunit F	EIF3F	-4.33	0.00176	No
P19404	NADH dehydrogenase [ubiquinone] flavoprotein 2, mitochondrial	NDUFV2	-4.33	2,00E-05	Yes
P09496	Clathrin light chain A	CLTA	-4.28	4.1e-4	No
Q08380	Galectin-3-binding protein	LGALS3BP	-4.28	0.00155	No
Q02218	2-oxoglutarate dehydrogenase, mitochondrial	OGDH	-4.23	2,00E-05	Yes
Q16875	6-phosphofructo-2-kinase/fructose-2,6-bisphosphatase 3	PFKFB3	-4.18	4.1e-4	No
O75380	NADH dehydrogenase [ubiquinone] iron-sulfur protein 6, mitochondrial	NDUFS6	-4.18	6,00E-05	Yes
Q9BRS2	Serine/threonine-protein kinase RIO1	RIOK1	-4.16	0.00497	No
Q9P270	SLAIN motif-containing protein 2	SLAIN2	-4.13	3.1e-4	No
Q99613	Eukaryotic translation initiation factor 3 subunit C	EIF3C	-4.12	0.00326	No
Q8NBJ5	Procollagen galactosyltransferase 1	COLGALT1	-4.12	9.2e-4	No
P10599	Thioredoxin	TXN	-4.09	1.6e-4	No
P16278	Beta-galactosidase	GLB1	-4.05	6,00E-04	No
Q8NBS9	Thioredoxin domain-containing protein 5	TXNDC5	-4.05	0.00352	No
P10515	Dihydrolipoyllysine-residue acetyltransferase component of pyruvate dehydrogenase complex, mitochondrial	DLAT	-4.04	1.1e-4	Yes
O15550	Lysine-specific demethylase 6A	KDM6A	-4.04	9.8e-4	No
P11177	Pyruvate dehydrogenase E1 component subunit beta, mitochondrial	PDHB	-4.02	4,00E-05	Yes

P36957	Dihydrolipoyllysine-residue succinyltransferase component of 2-oxoglutarate dehydrogenase complex, mitochondrial	DLST	-3.97	3.1e-4	Yes
Q14247	Src substrate cortactin	CTTN	-3.97	0.00159	No
Q15208	Serine/threonine-protein kinase 38	STK38	-3.95	2.7e-4	No
Q15075	Early endosome antigen 1	EEA1	-3.94	8.7e-4	No
Q13347	Eukaryotic translation initiation factor 3 subunit I	EIF3I	-3.93	0.00487	No
Q9NYJ1	Cytochrome c oxidase assembly factor 4 homolog, mitochondrial	COA4	-3.92	0.00129	Yes
Q15046	Lysine--tRNA ligase	KARS	-3.92	5.6e-4	Yes
P17612	cAMP-dependent protein kinase catalytic subunit alpha	PRKACA	-3.88	0.00195	No
P08559	Pyruvate dehydrogenase E1 component subunit alpha, somatic form, mitochondrial	PDHA1	-3.88	9,00E-05	Yes
A0A087WWU8	Tropomyosin alpha-3 chain	TPM3	-3.85	0.00271	No
Q9Y2S7	Polymerase delta-interacting protein 2	POLDIP2	-3.8	1,00E-05	Yes
Q14152	Eukaryotic translation initiation factor 3 subunit A	EIF3A	-3.77	0.01378	No
P27708	CAD protein	CAD	-3.74	0.00732	No
Q7Z4H8	KDEL motif-containing protein 2	KDELC2	-3.74	0.00231	No
O15371	Eukaryotic translation initiation factor 3 subunit D	EIF3D	-3.72	0.00856	No
O43181	NADH dehydrogenase [ubiquinone] iron-sulfur protein 4, mitochondrial	NDUFS4	-3.72	1.2e-4	Yes
P63104	14-3-3 protein zeta/delta	YWHAZ	-3.69	0.0022	No
P61978	Heterogeneous nuclear ribonucleoprotein K	HNRNPK	-3.67	3,00E-05	No
Q9ULV4	Coronin-1C	CORO1C	-3.64	7.7e-4	No
Q9Y3D2	Methionine-R-sulfoxide reductase B2, mitochondrial	MSRB2	-3.64	0.00201	Yes
P36551	Oxygen-dependent coproporphyrinogen-III oxidase, mitochondrial	CPOX	-3.63	2.1e-4	Yes
Q15293	Reticulocalbin-1	RCN1	-3.63	0.00447	No
Q9BUA3	SPIN1-docking protein	C11orf84	-3.63	0.00395	No
P15311	Ezrin	EZR	-3.62	0.00347	No
Q92896	Golgi apparatus protein 1	GLG1	-3.61	5,00E-05	No
A3KMH1	von Willebrand factor A domain-containing protein 8	VWA8	-3.61	8.2e-4	Yes
P07814	Bifunctional glutamate/proline--tRNA ligase	EPRS	-3.6	0.01803	No
P35580	Myosin-10	MYH10	-3.57	0.02659	No
P98175	RNA-binding protein 10	RBM10	-3.55	0.00422	No
Q8ND56	Protein LSM14 homolog A	LSM14A	-3.54	0.00204	No
P31751	RAC-beta serine/threonine-protein kinase	AKT2	-3.54	0.00888	No
Q9HCN8	Stromal cell-derived factor 2-like protein 1	SDF2L1	-3.53	0.01147	No
Q9H857	5'-nucleotidase domain-containing protein 2	NT5DC2	-3.52	0.00148	Yes
P52907	F-actin-capping protein subunit alpha-1	CAPZA1	-3.52	0.00264	No

P60891	Ribose-phosphate pyrophosphokinase 1	PRPS1	-3.48	0.00445	No
Q9Y657	Spindlin-1	SPIN1	-3.48	0.00865	No
O00330	Pyruvate dehydrogenase protein X component, mitochondrial	PDHX	-3.46	3.3e-4	Yes
O15372	Eukaryotic translation initiation factor 3 subunit H	EIF3H	-3.45	0.04775	No
Q13423	NAD(P) transhydrogenase, mitochondrial	NNT	-3.45	7.7e-4	Yes
H7BYW6	Platelet-derived growth factor subunit A (Fragment)	PDGFA	-3.45	0.00237	No
Q8IXB1	DnaJ homolog subfamily C member 10	DNAJC10	-3.44	0.00486	No
Q5JTJ3	Cytochrome c oxidase assembly factor 6 homolog	COA6	-3.43	6.1e-4	Yes
P50395	Rab GDP dissociation inhibitor beta	GDI2	-3.43	1.7e-4	No
O94915	Protein furry homolog-like	FRYL	-3.41	0.00729	No
P07203	Glutathione peroxidase 1	GPX1	-3.39	0.0029	Yes
P30101	Protein disulfide-isomerase A3	PDIA3	-3.36	0.00196	No
Q16775	Hydroxyacylglutathione hydrolase, mitochondrial	HAGH	-3.35	0.0351	Yes
Q9NVA2	Septin-11	11-Sep	-3.35	0.01982	No
Q9HCC0	Methylcrotonoyl-CoA carboxylase beta chain, mitochondrial	MCCC2	-3.33	2.2e-4	Yes
P42356	Phosphatidylinositol 4-kinase alpha	PI4KA	-3.33	0.03667	Yes
P12694	2-oxoisovalerate dehydrogenase subunit alpha, mitochondrial	BCKDHA	-3.32	6.4e-4	Yes
Q13162	Peroxiredoxin-4	PRDX4	-3.32	0.02531	Yes
P19105	Myosin regulatory light chain 12A	MYL12A	-3.29	0.0068	No
P35579	Myosin-9	MYH9	-3.29	0.01758	No
Q8N5W9	Refilin-B	RFLNB	-3.29	0.00856	No
Q9UBQ5	Eukaryotic translation initiation factor 3 subunit K	EIF3K	-3.28	0.01697	No
Q96BP2	Coiled-coil-helix-coiled-coil-helix domain-containing protein 1	CHCHD1	-3.26	1.3e-4	Yes
P14866	Heterogeneous nuclear ribonucleoprotein L	HNRNPL	-3.26	0.00207	No
Q04837	Single-stranded DNA-binding protein, mitochondrial	SSBP1	-3.25	9,00E-05	Yes
O43707	Alpha-actinin-4	ACTN4	-3.23	0.00729	No
P31946	14-3-3 protein beta/alpha	YWHAB	-3.22	5.7e-4	No
Q16134	Electron transfer flavoprotein-ubiquinone oxidoreductase, mitochondrial	ETFDH	-3.22	0.02387	Yes
P47756	F-actin-capping protein subunit beta	CAPZB	-3.21	0.01216	No
O14979	Heterogeneous nuclear ribonucleoprotein D-like	HNRNPDL	-3.17	7.2e-4	No
P13010	X-ray repair cross-complementing protein 5	XRCC5	-3.13	0.02226	No
Q96IX5	Up-regulated during skeletal muscle growth protein 5	USMG5	-3.11	0.01142	Yes
P60228	Eukaryotic translation initiation factor 3 subunit E	EIF3E	-3.1	0.01625	No
P84090	Enhancer of rudimentary homolog	ERH	-3.06	4.1e-4	No
P14314	Glucosidase 2 subunit beta	PRKCSH	-3.06	1.5e-4	No
P53007	Tricarboxylate transport protein, mitochondrial	SLC25A1	-3.06	0.03642	Yes
P09497	Clathrin light chain B	CLTB	-3.05	0.00239	No

Q9BW92	Threonine--tRNA ligase, mitochondrial	TARS2	-3.04	0.00157	Yes
P23381	Tryptophan--tRNA ligase, cytoplasmic	WARS	-3.04	0.00208	No
P11182	Lipoamide acyltransferase component of branched-chain alpha-keto acid dehydrogenase complex, mitochondrial	DBT	-3.02	1,00E-04	Yes
Q5T5U3	Rho GTPase-activating protein 21	ARHGAP21	-3.02	0.00403	No
Q05707	Collagen alpha-1(XIV) chain	COL14A1	-2.99	0.02628	No
Q04917	14-3-3 protein eta	YWHAH	-2.98	0.00237	No
Q14011	Cold-inducible RNA-binding protein	CIRBP	-2.98	0.08014	No
P11310	Medium-chain specific acyl-CoA dehydrogenase, mitochondrial	ACADM	-2.98	0.00655	Yes
P67775	Serine/threonine-protein phosphatase 2A catalytic subunit alpha isoform	PPP2CA	-2.98	0.01888	No
Q99497	Protein/nucleic acid deglycase DJ-1	PARK7	-2.95	0.00305	Yes
Q9Y241	HIG1 domain family member 1A, mitochondrial	HIGD1A	-2.94	0.00564	Yes
Q9NYL9	Tropomodulin-3	TMOD3	-2.94	9.4e-4	No
Q9NX40	OCIA domain-containing protein 1	OCIAD1	-2.92	0.01528	Yes
O60506	Heterogeneous nuclear ribonucleoprotein Q	SYNCRIP	-2.91	3.4e-4	No
Q16795	NADH dehydrogenase [ubiquinone] 1 alpha subcomplex subunit 9, mitochondrial	NDUFA9	-2.91	0.00616	Yes
P61163	Alpha-centractin	ACTR1A	-2.9	0.04702	No
Q8N4Q1	Mitochondrial intermembrane space import and assembly protein 40	CHCHD4	-2.9	0.00259	Yes
Q15393	Splicing factor 3B subunit 3	SF3B3	-2.9	0.01161	No
O96000	NADH dehydrogenase [ubiquinone] 1 beta subcomplex subunit 10	NDUFB10	-2.88	0.00119	Yes
P29966	Myristoylated alanine-rich C-kinase substrate	MARCKS	-2.87	0.03226	No
Q8IV08	Phospholipase D3	PLD3	-2.87	0.0097	No
O43678	NADH dehydrogenase [ubiquinone] 1 alpha subcomplex subunit 2	NDUFA2	-2.85	0.01088	Yes
Q02809	Procollagen-lysine,2-oxoglutarate 5-dioxygenase 1	PLOD1	-2.85	0.00247	No
Q96C01	Protein FAM136A	FAM136A	-2.85	0.0186	Yes
A8K968	Band 4.1-like protein 3	EPB41L3	-2.84	0.13304	No
P27797	Calreticulin	CALR	-2.84	0.02614	No
Q86U42	Polyadenylate-binding protein 2	PABPN1	-2.82	0.00578	No
Q49A26	Putative oxidoreductase GLYR1	GLYR1	-2.82	0.00152	No
Q09028	Histone-binding protein RBBP4	RBBP4	-2.81	0.01122	No
Q14257	Reticulocalbin-2	RCN2	-2.78	0.06329	Yes
P62306	Small nuclear ribonucleoprotein F	SNRPF	-2.77	0.02224	No
H7C0W7	Hyccin (Fragment)	FAM126A	-2.74	0.01868	No
Q8TEM1	Nuclear pore membrane glycoprotein 210	NUP210	-2.74	0.02015	No
P00403	Cytochrome c oxidase subunit 2	MT-CO2	-2.72	0.00594	Yes
Q9Y6M9	NADH dehydrogenase [ubiquinone] 1 beta subcomplex subunit 9	NDUFB9	-2.71	3.4e-4	Yes

Q8TCS8	Polyribonucleotide nucleotidyltransferase 1, mitochondrial	PNPT1	-2.71	0.02922	Yes
Q9BU61	NADH dehydrogenase [ubiquinone] 1 alpha subcomplex assembly factor 3	NDUFAF3	-2.7	0.00888	Yes
Q5W0B1	RING finger protein 219	RNF219	-2.69	0.0391	No
Q70IA6	MOB kinase activator 2	MOB2	-2.68	0.01459	No
Q5HYK3	2-methoxy-6-polyprenyl-1,4-benzoquinol methylase, mitochondrial	COQ5	-2.66	0.01912	Yes
Q9Y4E8	Ubiquitin carboxyl-terminal hydrolase 15	USP15	-2.66	4.6e-4	No
P61604	10 kDa heat shock protein, mitochondrial	HSPE1	-2.65	0.00806	Yes
P05091	Aldehyde dehydrogenase, mitochondrial	ALDH2	-2.65	0.00981	Yes
O75083	WD repeat-containing protein 1	WDR1	-2.65	0.00175	No
Q9Y5J7	Mitochondrial import inner membrane translocase subunit Tim9	TIMM9	-2.64	0.00868	Yes
P22392	Nucleoside diphosphate kinase B	NME2	-2.64	0.00732	No
P54819	Adenylate kinase 2, mitochondrial	AK2	-2.61	0.02087	Yes
P06865	Beta-hexosaminidase subunit alpha	HEXA	-2.61	0.00497	No
P10606	Cytochrome c oxidase subunit 5B, mitochondrial	COX5B	-2.61	4.7e-4	Yes
P12956	X-ray repair cross-complementing protein 6	XRCC6	-2.6	0.01267	No
Q69YU5	Uncharacterized protein C12orf73	C12orf73	-2.57	0.00466	No
Q14126	Desmoglein-2	DSG2	-2.56	0.00182	No
P23458	Tyrosine-protein kinase JAK1	JAK1	-2.53	0.14581	No
Q53S33	BolA-like protein 3	BOLA3	-2.52	0.00759	Yes
Q9NYU2	UDP-glucose:glycoprotein glucosyltransferase 1	UGGT1	-2.52	0.00649	No
P62258	14-3-3 protein epsilon	YWHAE	-2.51	3.6e-4	No
P00505	Aspartate aminotransferase, mitochondrial	GOT2	-2.51	0.02099	Yes
P49748	Very long-chain specific acyl-CoA dehydrogenase, mitochondrial	ACADVL	-2.5	0.04578	Yes
P56381	ATP synthase subunit epsilon, mitochondrial	ATP5F1E	-2.49	0.23939	Yes
O76031	ATP-dependent Clp protease ATP-binding subunit clpX-like, mitochondrial	CLPX	-2.48	0.02484	Yes
P07942	Laminin subunit beta-1	LAMB1	-2.48	0.00206	No
O43920	NADH dehydrogenase [ubiquinone] iron-sulfur protein 5	NDUFS5	-2.48	0.00681	Yes
P21796	Voltage-dependent anion-selective channel protein 1	VDAC1	-2.48	0.00948	Yes
Q8N3Z3	GTP-binding protein 8	GTPBP8	-2.46	0.02839	No
Q16625	Occludin	OCLN	-2.46	0.04831	No
Q9UJW0	Dynactin subunit 4	DCTN4	-2.45	0.00888	No
Q15120	[Pyruvate dehydrogenase (acetyl-transferring)] kinase isozyme 3, mitochondrial	PDK3	-2.44	0.06047	Yes
A0A075B6S2	Immunoglobulin kappa variable 2D-29	IGKV2D-29	-2.44	0.20472	No
Q9H2K0	Translation initiation factor IF-3, mitochondrial	MTIF3	-2.44	0.48086	Yes

Q99757	Thioredoxin, mitochondrial	TXN2	-2.43	0.01029	Yes
P40926	Malate dehydrogenase, mitochondrial	MDH2	-2.42	0.02657	Yes
Q9Y265	RuvB-like 1	RUVBL1	-2.42	0.00271	No
O15020	Spectrin beta chain, non-erythrocytic 2	SPTBN2	-2.41	0.06329	No
P31040	Succinate dehydrogenase [ubiquinone] flavoprotein subunit, mitochondrial	SDHA	-2.4	0.00412	Yes
P07686	Beta-hexosaminidase subunit beta	HEXB	-2.39	0.0791	No
Q53H12	Acylglycerol kinase, mitochondrial	AGK	-2.38	0.02922	Yes
P60059	Protein transport protein Sec61 subunit gamma	SEC61G	-2.36	0.00746	No
P30837	Aldehyde dehydrogenase X, mitochondrial	ALDH1B1	-2.35	0.00459	Yes
P06493	Cyclin-dependent kinase 1	CDK1	-2.35	0.08426	No
P32119	Peroxiredoxin-2	PRDX2	-2.35	0.00681	Yes
Q9UN37	Vacuolar protein sorting-associated protein 4A	VPS4A	-2.34	0.01817	No
P18859	ATP synthase-coupling factor 6, mitochondrial	ATP5J	-2.33	6.3e-4	Yes
P0DP23	Calmodulin-1	CALM1	-2.32	0.00898	No
Q9Y375	Complex I intermediate-associated protein 30, mitochondrial	NDUFAF1	-2.31	0.01803	Yes
Q9UKN8	General transcription factor 3C polypeptide 4	GTF3C4	-2.31	0.00406	No
P62136	Serine/threonine-protein phosphatase PP1-alpha catalytic subunit	PPP1CA	-2.3	0.14705	No
Q15942	Zyxin	ZYX	-2.28	0.05233	No
Q9NQZ2	Something about silencing protein 10	UTP3	-2.26	0.00681	No
Q13433	Zinc transporter ZIP6	SLC39A6	-2.25	0.0304	No
P08574	Cytochrome c1, heme protein, mitochondrial	CYC1	-2.24	0.01619	Yes
P42126	Enoyl-CoA delta isomerase 1, mitochondrial	ECI1	-2.24	0.02987	Yes
Q9P0J0	NADH dehydrogenase [ubiquinone] 1 alpha subcomplex subunit 13	NDUFA13	-2.23	0.02025	Yes
P80723	Brain acid soluble protein 1	BASP1	-2.22	0.01229	No
Q96H55	Unconventional myosin-XIX	MYO19	-2.22	0.01202	No
P60842	Eukaryotic initiation factor 4A-I	EIF4A1	-2.21	0.09192	No
Q09161	Nuclear cap-binding protein subunit 1	NCBP1	-2.21	0.01407	No
Q9Y3F4	Serine-threonine kinase receptor-associated protein	STRAP	-2.21	0.02268	No
Q5JRA6	Transport and Golgi organization protein 1 homolog	MIA3	-2.21	0.02595	No
P30533	Alpha-2-macroglobulin receptor-associated protein	LRPAP1	-2.2	0.00932	No
Q7Z3D6	D-glutamate cyclase, mitochondrial	DGLUCY	-2.19	0.08137	Yes
Q5VV42	Threonylcarbamoyladenosine tRNA methylthiotransferase	CDKAL1	-2.19	0.0246	No
O00232	26S proteasome non-ATPase regulatory subunit 12	PSMD12	-2.18	0.01342	No
O75880	Protein SCO1 homolog, mitochondrial	SCO1	-2.18	0.05699	Yes
E3W994	CLIP-associating protein 2	CLASP2	-2.17	0.11562	No
O00231	26S proteasome non-ATPase regulatory subunit 11	PSMD11	-2.16	0.00602	No
P14854	Cytochrome c oxidase subunit 6B1	COX6B1	-2.16	0.01152	Yes

Q5UIP0	Telomere-associated protein RIF1	RIF1	-2.16	0.05266	No
P00367	Glutamate dehydrogenase 1, mitochondrial	GLUD1	-2.15	0.00117	Yes
P17480	Nucleolar transcription factor 1	UBTF	-2.15	0.00764	No
O75323	Protein NipSnap homolog 2	NIPSNAP2	-2.14	0.10509	Yes
Q7Z7C8	Transcription initiation factor TFIID subunit 8	TAF8	-2.14	0.02782	No
P09012	U1 small nuclear ribonucleoprotein A	SNRPA	-2.13	0.02395	No
Q96BR5	Cytochrome c oxidase assembly factor 7	COA7	-2.1	0.00166	Yes
Q14203	Dynactin subunit 1	DCTN1	-2.08	0.00406	No
Q9NTJ3	Structural maintenance of chromosomes protein 4	SMC4	-2.08	0.0322	No
Q99798	Aconitate hydratase, mitochondrial	ACO2	-2.07	0.04775	Yes
P30405	Peptidyl-prolyl cis-trans isomerase F, mitochondrial	PPIF	-2.07	0.14056	Yes
Q13576	Ras GTPase-activating-like protein IQGAP2	IQGAP2	-2.07	0.05369	No
P23258	Tubulin gamma-1 chain	TUBG1	-2.07	0.03121	No
Q96NE9	FERM domain-containing protein 6	FRMD6	-2.06	0.01531	No
P04062	Glucosylceramidase	GBA	-2.06	0.02225	No
Q9UQE7	Structural maintenance of chromosomes protein 3	SMC3	-2.06	0.01227	No
P26641	Elongation factor 1-gamma	EEF1G	-2.05	0.01427	No
P08590	Myosin light chain 3	MYL3	-2.04	0.21325	No
P21912	Succinate dehydrogenase [ubiquinone] iron-sulfur subunit, mitochondrial	SDHB	-2.04	0.00807	Yes
P14868	Aspartate--tRNA ligase, cytoplasmic	DARS	-2.03	0.09376	No
B7ZM99	MTHFD1L protein	MTHFD1L	-2.03	0.02596	Yes
P56556	NADH dehydrogenase [ubiquinone] 1 alpha subcomplex subunit 6	NDUFA6	-2.03	0.03288	Yes
P62333	26S proteasome regulatory subunit 10B	PSMC6	-2.02	0.02025	No
P54136	Arginine--tRNA ligase, cytoplasmic	RARS	-2.02	0.13067	Yes
Q12904	Aminoacyl tRNA synthase complex-interacting multifunctional protein 1	AIMP1	-2.01	0.18136	No
P23528	Cofilin-1	CFL1	-2.01	0.02276	No
Q8NB49	Phospholipid-transporting ATPase IG	ATP11C	-1.99	0.04205	No
P30048	Thioredoxin-dependent peroxide reductase, mitochondrial	PRDX3	-1.99	0.00856	Yes
P42167	Lamina-associated polypeptide 2, isoforms beta/gamma	TMPO	-1.98	0.06563	No
P17568	NADH dehydrogenase [ubiquinone] 1 beta subcomplex subunit 7	NDUFB7	-1.98	0.01671	Yes
Q8WWV3	Reticulon-4-interacting protein 1, mitochondrial	RTN4IP1	-1.98	0.02395	Yes
Q14103	Heterogeneous nuclear ribonucleoprotein D0	HNRNPD	-1.97	0.01476	No
P62310	U6 snRNA-associated Sm-like protein LSm3	LSM3	-1.97	0.03881	No
P07919	Cytochrome b-c1 complex subunit 6, mitochondrial	UQCRH	-1.96	0.17602	Yes
Q06830	Peroxiredoxin-1	PRDX1	-1.96	0.00711	No
Q5T9A4	ATPase family AAA domain-containing protein 3B	ATAD3B	-1.95	0.04012	Yes
P31943	Heterogeneous nuclear ribonucleoprotein H	HNRNPH1	-1.95	0.00445	No

O15127	Secretory carrier-associated membrane protein 2	SCAMP2	-1.94	0.00711	No
P62072	Mitochondrial import inner membrane translocase subunit Tim10	TIMM10	-1.93	0.01281	Yes
P50213	Isocitrate dehydrogenase [NAD] subunit alpha, mitochondrial	IDH3A	-1.9	0.08105	Yes
Q9Y5J6	Mitochondrial import inner membrane translocase subunit Tim10 B	TIMM10B	-1.9	0.13882	Yes
Q8IXI1	Mitochondrial Rho GTPase 2	RHOT2	-1.9	0.02388	Yes
O95167	NADH dehydrogenase [ubiquinone] 1 alpha subcomplex subunit 3	NDUFA3	-1.9	0.09192	Yes
Q9BRK0	Receptor expression-enhancing protein 2	REEP2	-1.9	0.02922	No
P35998	26S proteasome regulatory subunit 7	PSMC2	-1.89	0.02193	No
Q6NUK1	Calcium-binding mitochondrial carrier protein SCaMC-1	SLC25A24	-1.89	0.01508	Yes
Q8TAA5	GrpE protein homolog 2, mitochondrial	GRPEL2	-1.88	0.03491	Yes
Q9Y6C9	Mitochondrial carrier homolog 2	MTCH2	-1.88	0.04561	Yes
P60660	Myosin light polypeptide 6	MYL6	-1.88	0.01494	No
D6R9P3	Heterogeneous nuclear ribonucleoprotein A/B	HNRNPAB	-1.87	0.01033	No
O43674	NADH dehydrogenase [ubiquinone] 1 beta subcomplex subunit 5, mitochondrial	NDUFB5	-1.87	0.0516	Yes
P24534	Elongation factor 1-beta	EEF1B2	-1.86	0.01618	No
Q8WUM4	Programmed cell death 6-interacting protein	PDCD6IP	-1.86	0.02034	No
P40938	Replication factor C subunit 3	RFC3	-1.86	0.02628	No
Q15008	26S proteasome non-ATPase regulatory subunit 6	PSMD6	-1.84	0.04261	No
P23368	NAD-dependent malic enzyme, mitochondrial	ME2	-1.83	0.08415	Yes
Q13794	Phorbol-12-myristate-13-acetate-induced protein 1	PMAIP1	-1.83	0.05403	Yes
Q96A26	Protein FAM162A	FAM162A	-1.83	0.11747	Yes
Q8N5C8	TGF-beta-activated kinase 1 and MAP3K7-binding protein 3	TAB3	-1.83	0.18663	No
Q5JTZ9	Alanine--tRNA ligase, mitochondrial	AARS2	-1.82	0.10036	Yes
Q9UBT7	Alpha-catulin	CTNNAL1	-1.81	0.00746	No
Q9NZB2	Constitutive coactivator of PPAR-gamma-like protein 1	FAM120A	-1.8	0.09628	No
Q8IWI9	MAX gene-associated protein	MGA	-1.8	0.11124	No
P63261	Actin, cytoplasmic 2	ACTG1	-1.78	0.0474	No
Q9H3K6	BolA-like protein 2	BOLA2; BOLA2B	-1.78	0.0246	No
O75746	Calcium-binding mitochondrial carrier protein Aralar1	SLC25A12	-1.78	0.07491	Yes
Q86SF2	N-acetylgalactosaminyltransferase 7	GALNT7	-1.78	0.04758	No
Q8N0V3	Putative ribosome-binding factor A, mitochondrial	RBFA	-1.78	0.00888	Yes
P46060	Ran GTPase-activating protein 1	RANGAP1	-1.78	0.08993	No
Q92575	UBX domain-containing protein 4	UBXN4	-1.78	0.0298	No
P19022	Cadherin-2	CDH2	-1.77	0.07408	No
O14519	Cyclin-dependent kinase 2-associated protein 1	CDK2AP1	-1.77	0.14946	No

P0DMV8	Heat shock 70 kDa protein 1A	HSPA1A	-1.77	0.00214	No
Q9Y5U8	Mitochondrial pyruvate carrier 1	MPC1	-1.76	0.00857	Yes
P22234	Multifunctional protein ADE2	PAICS	-1.76	0.07151	Yes
O95625	Zinc finger and BTB domain-containing protein 11	ZBTB11	-1.76	0.02159	No
Q6PI48	Aspartate--tRNA ligase, mitochondrial	DARS2	-1.75	0.10285	Yes
Q9NVH0	Exonuclease 3'-5' domain-containing protein 2	EXD2	-1.75	0.02358	No
Q96CP6	GRAM domain-containing protein 1A	GRAMD1A	-1.75	0.02387	No
O60220	Mitochondrial import inner membrane translocase subunit Tim8 A	TIMM8A	-1.75	0.00746	Yes
Q15084	Protein disulfide-isomerase A6	PDIA6	-1.75	0.01828	No
Q9NUP9	Protein lin-7 homolog C	LIN7C	-1.74	0.0302	No
P07602	Prosaposin	PSAP	-1.73	0.13082	No
Q9Y2W1	Thyroid hormone receptor-associated protein 3	THRAP3	-1.73	0.13882	No
Q9H845	Acyl-CoA dehydrogenase family member 9, mitochondrial	ACAD9	-1.72	0.22575	Yes
P01111	GTPase NRas	NRAS	-1.72	0.01932	No
P41252	Isoleucine--tRNA ligase, cytoplasmic	IARS	-1.72	0.12995	No
Q9ULF5	Zinc transporter ZIP10	SLC39A10	-1.72	0.02619	No
O75718	Cartilage-associated protein	CRTAP	-1.71	0.02877	No
Q9UHN6	Cell surface hyaluronidase	TMEM2	-1.71	0.04378	No
O95831	Apoptosis-inducing factor 1, mitochondrial	AIFM1	-1.7	0.00587	Yes
Q9UJS0	Calcium-binding mitochondrial carrier protein Aralar2	SLC25A13	-1.7	0.16526	Yes
P55072	Transitional endoplasmic reticulum ATPase	VCP	-1.7	0.01163	No
P35221	Catenin alpha-1	CTNNA1	-1.69	0.13799	No
P46977	Dolichyl-diphosphooligosaccharide--protein glycosyltransferase subunit STT3A	STT3A	-1.69	0.10514	No
P49257	Protein ERGIC-53	LMAN1	-1.68	0.15782	No
Q9UII2	ATPase inhibitor, mitochondrial	ATP5IF1	-1.67	0.1053	Yes
Q86YQ8	Copine-8	CPNE8	-1.67	0.09668	No
Q5XKP0	MICOS complex subunit MIC13	MIC13	-1.67	0.33795	Yes
P11021	Endoplasmic reticulum chaperone BiP	HSPA5	-1.66	0.0057	No
Q9H078	Caseinolytic peptidase B protein homolog	CLPB	-1.65	0.02034	Yes
Q96CT7	Coiled-coil domain-containing protein 124	CCDC124	-1.65	0.01796	No
Q08379	Golgin subfamily A member 2	GOLGA2	-1.65	0.07518	No
Q9BQP7	Mitochondrial genome maintenance exonuclease 1	MGME1	-1.65	0.07666	Yes
P13667	Protein disulfide-isomerase A4	PDIA4	-1.65	0.14025	No
Q99536	Synaptic vesicle membrane protein VAT-1 homolog	VAT1	-1.65	0.11136	No
Q9BRQ6	MICOS complex subunit MIC25	CHCHD6	-1.64	0.0519	Yes
O75915	PRA1 family protein 3	ARL6IP5	-1.64	0.09421	No

Q9BQ52	Zinc phosphodiesterase ELAC protein 2	ELAC2	-1.64	0.02387	Yes
P62195	26S proteasome regulatory subunit 8	PSMC5	-1.63	0.01982	No
P15924	Desmoplakin	DSP	-1.63	0.07425	No
Q9NVH6	Trimethyllysine dioxygenase, mitochondrial	TMLHE	-1.63	0.0801	Yes
Q8WWY3	U4/U6 small nuclear ribonucleoprotein Prp31	PRPF31	-1.63	0.25959	No
P45880	Voltage-dependent anion-selective channel protein 2	VDAC2	-1.62	0.03904	Yes
Q02127	Dihydroorotate dehydrogenase (quinone), mitochondrial	DHODH	-1.61	0.12576	Yes
P06576	ATP synthase subunit beta, mitochondrial	ATP5F1B	-1.6	0.03295	Yes
Q9UI43	rRNA methyltransferase 2, mitochondrial	MRM2	-1.6	0.04044	Yes
Q15111	Inactive phospholipase C-like protein 1	PLCL1	-1.59	0.04683	No
P12004	Proliferating cell nuclear antigen	PCNA	-1.59	0.10703	No
O15116	U6 snRNA-associated Sm-like protein LSm1	LSM1	-1.59	0.14458	No
O15173	Membrane-associated progesterone receptor component 2	PGRMC2	-1.57	0.02657	No
P54098	DNA polymerase subunit gamma-1	POLG	-1.56	0.13054	Yes
Q9Y6H1	Coiled-coil-helix-coiled-coil-helix domain-containing protein 2	CHCHD2	-1.55	0.02849	Yes
Q9Y5Y0	Feline leukemia virus subgroup C receptor-related protein 1	FLVCR1	-1.55	0.20645	No
P49821	NADH dehydrogenase [ubiquinone] flavoprotein 1, mitochondrial	NDUFV1	-1.55	0.25413	Yes
Q9UDW1	Cytochrome b-c1 complex subunit 9	UQCR10	-1.54	0.05444	Yes
O75306	NADH dehydrogenase [ubiquinone] iron-sulfur protein 2, mitochondrial	NDUFS2	-1.54	0.03757	Yes
Q9NP72	Ras-related protein Rab-18	RAB18	-1.54	0.0488	No
O14925	Mitochondrial import inner membrane translocase subunit Tim23	TIMM23	-1.53	0.11488	Yes
P26038	Moesin	MSN	-1.53	0.06959	No
Q6UW78	Ubiquinol-cytochrome-c reductase complex assembly factor 3	UQCC3	-1.53	0.11759	No
Q8NI60	Atypical kinase COQ8A, mitochondrial	COQ8A	-1.52	0.16415	Yes
Q9Y394	Dehydrogenase/reductase SDR family member 7	DHRS7	-1.52	0.05842	No
P14625	Endoplasmin	HSP90B1	-1.52	0.01543	No
P51970	NADH dehydrogenase [ubiquinone] 1 alpha subcomplex subunit 8	NDUFA8	-1.52	0.0095	Yes
O14949	Cytochrome b-c1 complex subunit 8	UQCRQ	-1.51	0.05369	Yes
O00483	Cytochrome c oxidase subunit NDUFA4	NDUFA4	-1.51	0.10408	Yes
P42765	3-ketoacyl-CoA thiolase, mitochondrial	ACAA2	-1.49	0.33667	Yes
P10155	60 kDa SS-A/Ro ribonucleoprotein	TROVE2	-1.49	0.03541	No
P56181	NADH dehydrogenase [ubiquinone] flavoprotein 3, mitochondrial	NDUFV3	-1.49	0.04831	Yes
Q86UT6	NLR family member X1	NLRX1	-1.49	0.06329	Yes
Q9ULX3	RNA-binding protein NOB1	NOB1	-1.49	0.23939	No
P21964	Catechol O-methyltransferase	COMT	-1.48	0.20861	Yes
P49368	T-complex protein 1 subunit gamma	CCT3	-1.48	0.26056	No
P10644	cAMP-dependent protein kinase type I-alpha regulatory subunit	PRKAR1A	-1.47	0.07456	No

P49902	Cytosolic purine 5'-nucleotidase	NT5C2	-1.47	0.13882	No
P11142	Heat shock cognate 71 kDa protein	HSPA8	-1.47	0.00681	No
Q5VT66	Mitochondrial amidoxime-reducing component 1	01-Mar	-1.47	0.25323	Yes
O75438	NADH dehydrogenase [ubiquinone] 1 beta subcomplex subunit 1	NDUFB1	-1.47	0.01342	Yes
Q9Y5S9	RNA-binding protein 8A	RBM8A	-1.47	0.17855	No
Q9NXH9	tRNA (guanine(26)-N(2))-dimethyltransferase	TRMT1	-1.47	0.0181	Yes
E7EX44	Caldesmon	CALD1	-1.46	0.19754	No
Q9BRT2	Ubiquinol-cytochrome-c reductase complex assembly factor 2	UQCC2	-1.46	0.23761	Yes
Q9Y5K6	CD2-associated protein	CD2AP	-1.45	0.07313	No
Q14204	Cytoplasmic dynein 1 heavy chain 1	DYNC1H1	-1.45	0.3932	No
O75955	Flotillin-1	FLOT1	-1.45	0.03098	No
O95881	Thioredoxin domain-containing protein 12	TXNDC12	-1.45	0.22926	Yes
Q01518	Adenylyl cyclase-associated protein 1	CAP1	-1.44	0.19408	No
P31930	Cytochrome b-c1 complex subunit 1, mitochondrial	UQCRC1	-1.44	0.01949	Yes
P08238	Heat shock protein HSP 90-beta	HSP90AB1	-1.44	0.0474	No
Q9H7Z7	Prostaglandin E synthase 2	PTGES2	-1.44	0.33683	Yes
O43464	Serine protease HTRA2, mitochondrial	HTRA2	-1.44	0.05356	Yes
A0A087WU M0	SYNJ2BP-COX16 readthrough (Fragment)	SYNJ2BP-COX16	-1.44	0.20169	No
Q12846	Syntaxin-4	STX4	-1.44	0.07154	No
P14927	Cytochrome b-c1 complex subunit 7	UQCRB	-1.43	0.02735	Yes
P51153	Ras-related protein Rab-13	RAB13	-1.43	0.18206	No
Q13200	26S proteasome non-ATPase regulatory subunit 2	PSMD2	-1.42	0.08562	No
O95139	NADH dehydrogenase [ubiquinone] 1 beta subcomplex subunit 6	NDUFB6	-1.42	0.0519	Yes
P20020	Plasma membrane calcium-transporting ATPase 1	ATP2B1	-1.42	0.35056	No
Q96HJ9	Protein FMC1 homolog	FMC1	-1.42	0.05869	Yes
Q9Y6N5	Sulfide:quinone oxidoreductase, mitochondrial	SQOR	-1.42	0.14115	Yes
O75439	Mitochondrial-processing peptidase subunit beta	PMPCB	-1.41	0.3132	Yes
Q9UHQ9	NADH-cytochrome b5 reductase 1	CYB5R1	-1.41	0.09239	No
Q5JPH6	Probable glutamate--tRNA ligase, mitochondrial	EARS2	-1.41	0.08486	Yes
Q9NYY8	FAST kinase domain-containing protein 2, mitochondrial	FASTKD2	-1.4	0.17209	Yes
Q8NE01	Metal transporter CNNM3	CNNM3	-1.4	0.11124	No
Q96JX3	Protein SERAC1	SERAC1	-1.4	0.03222	Yes
Q13011	Delta(3,5)-Delta(2,4)-dienoyl-CoA isomerase, mitochondrial	ECH1	-1.39	0.33875	Yes
Q04721	Neurogenic locus notch homolog protein 2	NOTCH2	-1.39	0.19705	No
P51148	Ras-related protein Rab-5C	RAB5C	-1.39	0.14836	No
P30049	ATP synthase subunit delta, mitochondrial	ATP5F1D	-1.38	0.04164	Yes

A6NJ78	Probable methyltransferase-like protein 15	METTL15	-1.38	0.06298	Yes
O15235	28S ribosomal protein S12, mitochondrial (uS12m)	MRPS12	-1.37	0.02683	Yes
Q5ZPR3	CD276 antigen	CD276	-1.37	0.08486	No
Q86X29	Lipolysis-stimulated lipoprotein receptor	LSR	-1.37	0.10408	No
P62937	Peptidyl-prolyl cis-trans isomerase A	PPIA	-1.37	0.21762	No
Q9Y277	Voltage-dependent anion-selective channel protein 3	VDAC3	-1.37	0.21383	Yes
Q96GK7	Fumarylacetoacetate hydrolase domain-containing protein 2A	FAHD2A	-1.35	0.02151	Yes
P48651	Phosphatidylserine synthase 1	PTDSS1	-1.35	0.1017	No
P20339	Ras-related protein Rab-5A	RAB5A	-1.35	0.10036	No
Q96DZ1	Endoplasmic reticulum lectin 1	ERLEC1	-1.34	0.14315	No
Q9UPT5	Exocyst complex component 7	EXOC7	-1.34	0.05778	No
Q9UBI6	Guanine nucleotide-binding protein G(I)/G(S)/G(O) subunit gamma-12	GNG12	-1.34	0.15903	No
O43837	Isocitrate dehydrogenase [NAD] subunit beta, mitochondrial	IDH3B	-1.34	0.05358	Yes
Q9BZF1	Oxysterol-binding protein-related protein 8	OSBPL8	-1.34	0.0298	No
Q2TAY7	WD40 repeat-containing protein SMU1	SMU1	-1.34	0.07043	No
Q14839	Chromodomain-helicase-DNA-binding protein 4	CHD4	-1.33	0.55421	No
Q13308	Inactive tyrosine-protein kinase 7	PTK7	-1.33	0.20169	No
Q9H061	Transmembrane protein 126A	TMEM126A	-1.33	0.1672	Yes
Q9Y399	28S ribosomal protein S2, mitochondrial (uS2m)	MRPS2	-1.32	0.04029	Yes
Q9NRK6	ATP-binding cassette sub-family B member 10, mitochondrial	ABCB10	-1.32	0.42396	Yes
Q7L2H7	Eukaryotic translation initiation factor 3 subunit M	EIF3M	-1.32	0.19098	No
P49327	Fatty acid synthase	FASN	-1.32	0.17056	Yes
Q8N2K0	Monoacylglycerol lipase ABHD12	ABHD12	-1.32	0.12042	No
Q16181	Septin-7	07-Sep	-1.32	0.2781	No
Q15005	Signal peptidase complex subunit 2	SPCS2	-1.32	0.10646	No
P62318	Small nuclear ribonucleoprotein Sm D3	SNRPD3	-1.32	0.02287	No
Q9NW81	Distal membrane-arm assembly complex protein 2	DMAC2	-1.31	0.08806	Yes
Q02750	Dual specificity mitogen-activated protein kinase kinase 1	MAP2K1	-1.31	0.17916	No
P08069	Insulin-like growth factor 1 receptor	IGF1R	-1.31	0.04775	No
Q9BSF4	Mitochondrial import inner membrane translocase subunit Tim29	TIMM29	-1.31	0.09778	Yes
Q9Y512	Sorting and assembly machinery component 50 homolog	SAMM50	-1.31	0.15732	Yes
Q9UNL2	Translocon-associated protein subunit gamma	SSR3	-1.31	0.14137	No
P55196	Afadin	AFDN	-1.3	0.15532	No
Q9UM00	Calcium load-activated calcium channel	TMCO1	-1.3	0.22457	No
Q92616	eIF-2-alpha kinase activator GCN1	GCN1	-1.3	0.20231	No
Q6P4A7	Sideroflexin-4	SFXN4	-1.3	0.27397	Yes
Q15904	V-type proton ATPase subunit S1	ATP6AP1	-1.3	0.10408	No

Q9Y230	RuvB-like 2	RUVBL2	-1.29	0.19433	No
O95248	Myotubularin-related protein 5	SBF1	-1.28	0.16438	No
P51149	Ras-related protein Rab-7a	RAB7A	-1.28	0.38645	No
P38159	RNA-binding motif protein, X chromosome	RBMX	-1.28	0.22575	No
Q14739	Lamin-B receptor	LBR	-1.27	0.09954	No
P17980	26S proteasome regulatory subunit 6A	PSMC3	-1.26	0.08844	No
Q969Z0	FAST kinase domain-containing protein 4	TBRG4	-1.26	0.04606	Yes
Q14697	Neutral alpha-glucosidase AB	GANAB	-1.26	0.1224	No
Q9BWJ5	Splicing factor 3B subunit 5	SF3B5	-1.26	0.096560000000000007	No
Q9H2U1	ATP-dependent RNA helicase DHX36	DHX36	-1.25	0.33875	No
Q9BT22	Chitobiosyldiphosphodolichol beta-mannosyltransferase	ALG1	-1.25	0.08182	No
Q14008	Cytoskeleton-associated protein 5	CKAP5	-1.25	0.28267	No
P84095	Rho-related GTP-binding protein RhoG	RHOG	-1.25	0.11357	No
E5KLJ9	Dynamin-like 120 kDa protein, mitochondrial	OPA1	-1.24	0.32623	Yes
P30519	Heme oxygenase 2	HMOX2	-1.24	0.39689	No
P50336	Protoporphyrinogen oxidase	PPOX	-1.24	0.11279	Yes
Q8N5G0	Small integral membrane protein 20	SMIM20	-1.24	0.11723	No
Q9HC07	Transmembrane protein 165	TMEM165	-1.24	0.09301	No
O14773	Tripeptidyl-peptidase 1	TPP1	-1.24	0.36705	No
O95983	Methyl-CpG-binding domain protein 3	MBD3	-1.22	0.27465	No
Q8IYB8	ATP-dependent RNA helicase SUPV3L1, mitochondrial	SUPV3L1	-1.21	0.22312	Yes
O43324	Eukaryotic translation elongation factor 1 epsilon-1	EEF1E1	-1.21	0.14137	No
Q9HBH5	Retinol dehydrogenase 14	RDH14	-1.21	0.30085	Yes
Q9Y6D0	Selenoprotein K	SELENOK	-1.21	0.21359	No
Q9NRP4	Succinate dehydrogenase assembly factor 3, mitochondrial	SDHAF3	-1.21	0.1648	Yes
P49755	Transmembrane emp24 domain-containing protein 10	TMED10	-1.21	0.25512	No
Q9NRP2	COX assembly mitochondrial protein 2 homolog	CMC2	-1.2	0.29705	Yes
O60488	Long-chain-fatty-acid--CoA ligase 4	ACSL4	-1.2	0.19336	Yes
Q13901	Nuclear nucleic acid-binding protein C1D	C1D	-1.2	0.20017	No
P63208	S-phase kinase-associated protein 1	SKP1	-1.2	0.06007	No
Q9UPQ9	Trinucleotide repeat-containing gene 6B protein	TNRC6B	-1.2	0.34851	No
Q9BQE3	Tubulin alpha-1C chain	TUBA1C	-1.2	0.07843	No
Q9Y5A9	YTH domain-containing family protein 2	YTHDF2	-1.2	0.16857	No
Q8WWI1	LIM domain only protein 7	LMO7	-1.19	0.18173	No
Q16822	Phosphoenolpyruvate carboxykinase [GTP], mitochondrial	PCK2	-1.19	0.03816	Yes
Q9UHA4	Ragulator complex protein LAMTOR3	LAMTOR3	-1.19	0.15351	No

P35637	RNA-binding protein FUS	FUS	-1.19	0.27557	No
O96019	Actin-like protein 6A	ACTL6A	-1.17	0.09344	No
Q9NP97	Dynein light chain roadblock-type 1	DYNLRB1	-1.17	0.30108	No
Q9BS26	Endoplasmic reticulum resident protein 44	ERP44	-1.17	0.11136	No
Q9Y2Z9	Ubiquinone biosynthesis monooxygenase COQ6, mitochondrial	COQ6	-1.17	0.32909	Yes
P26196	Probable ATP-dependent RNA helicase DDX6	DDX6	-1.16	0.19772	No
Q9UBV2	Protein sel-1 homolog 1	SEL1L	-1.16	0.0929	No
Q6ZRP7	Sulfhydryl oxidase 2	QSOX2	-1.16	0.33425	No
Q9H0D6	5'-3' exoribonuclease 2	XRN2	-1.15	0.30516	No
Q9H936	Mitochondrial glutamate carrier 1	SLC25A22	-1.15	0.23092	Yes
Q9Y3D9	28S ribosomal protein S23, mitochondrial (mS23)	MRPS23	-1.13	0.04775	Yes
O75027	ATP-binding cassette sub-family B member 7, mitochondrial	ABCB7	-1.13	0.36097	Yes
Q86VU5	Catechol O-methyltransferase domain-containing protein 1	COMTD1	-1.13	0.19754	Yes
Q10570	Cleavage and polyadenylation specificity factor subunit 1	CPSF1	-1.13	0.1648	No
Q96JJ3	Engulfment and cell motility protein 2	ELMO2	-1.13	0.13406	No
Q14165	Malectin	MLEC	-1.13	0.25544	No
Q9Y5L4	Mitochondrial import inner membrane translocase subunit Tim13	TIMM13	-1.13	0.04827	Yes
Q13015	Protein AF1q	MLLT11	-1.13	0.24559	No
Q9UHB9	Signal recognition particle subunit SRP68	SRP68	-1.13	0.11629	No
Q9NXW2	DnaJ homolog subfamily B member 12	DNAJB12	-1.12	0.11769	No
Q9HC21	Mitochondrial thiamine pyrophosphate carrier	SLC25A19	-1.12	0.14298	Yes
P30044	Peroxiredoxin-5, mitochondrial	PRDX5	-1.12	0.2781	Yes
O14980	Exportin-1	XPO1	-1.11	0.26766	No
A8MXV4	Nucleoside diphosphate-linked moiety X motif 19	NUDT19	-1.11	0.33912	Yes
P35232	Prohibitin	PHB	-1.11	0.0801	Yes
P50454	Serpin H1	SERPINH1	-1.11	0.34591	No
P62191	26S proteasome regulatory subunit 4	PSMC1	-1.1	0.10408	No
O14967	Calmegin	CLGN	-1.1	0.16812	No
P42285	Exosome RNA helicase MTR4	MTREX	-1.1	0.39402	No
O94766	Galactosylgalactosylxylosylprotein 3-beta-glucuronosyltransferase 3	B3GAT3	-1.1	0.12957	No
P84157	Matrix-remodeling-associated protein 7	MXRA7	-1.1	0.10034	No
Q9H9B4	Sideroflexin-1	SFXN1	-1.1	0.05878	Yes
Q9BVS5	tRNA (adenine(58)-N(1))-methyltransferase, mitochondrial	TRMT61B	-1.1	0.21142	Yes
P56378	6.8 kDa mitochondrial proteolipid	MP68	-1.09	0.1648	Yes
Q9UDR5	Alpha-aminoadipic semialdehyde synthase, mitochondrial	AASS	-1.09	0.2806	Yes
P05026	Sodium/potassium-transporting ATPase subunit beta-1	ATP1B1	-1.09	0.39689	No
P26440	Isovaleryl-CoA dehydrogenase, mitochondrial	IVD	-1.08	0.20632	Yes

P51665	26S proteasome non-ATPase regulatory subunit 7	PSMD7	-1.07	0.21793	No
P82912	28S ribosomal protein S11, mitochondrial (uS11m)	MRPS11	-1.07	0.05369	Yes
Q96HY6	DDRGK domain-containing protein 1	DDRGK1	-1.07	0.11103	No
Q969V3	Nicalin	NCLN	-1.07	0.3132	No
Q9BPW8	Protein NipSnap homolog 1	NIPSNAP1	-1.07	0.19324	Yes
Q9NUQ6	SPATS2-like protein	SPATS2L	-1.07	0.15469	No
Q9P0L0	Vesicle-associated membrane protein-associated protein A	VAPA	-1.07	0.24385	No
Q8TAG9	Exocyst complex component 6	EXOC6	-1.06	0.1351	No
Q15599	Na(+)/H(+) exchange regulatory cofactor NHE-RF2	SLC9A3R2	-1.06	0.2928	No
Q58FF8	Putative heat shock protein HSP 90-beta 2	HSP90AB2P	-1.06	0.14025	No
Q96I51	RCC1-like G exchanging factor-like protein	RCC1L	-1.06	0.29741	Yes
O43175	D-3-phosphoglycerate dehydrogenase	PHGDH	-1.05	0.19772	No
Q15019	Septin-2	02-Sep	-1.05	0.36097	No
P09669	Cytochrome c oxidase subunit 6C	COX6C	-1.04	0.1017	Yes
P29692	Elongation factor 1-delta	EEF1D	-1.04	0.19324	No
Q8NBJ4	Golgi membrane protein 1	GOLM1	-1.04	0.2866	No
P14618	Pyruvate kinase PKM	PKM	-1.04	0.50118	No
P04626	Receptor tyrosine-protein kinase erbB-2	ERBB2	-1.04	0.21142	No
Q99943	1-acyl-sn-glycerol-3-phosphate acyltransferase alpha	AGPAT1	-1.03	0.25768	No
P56385	ATP synthase subunit e, mitochondrial	ATP5ME	-1.03	0.11136	Yes
Q9P032	NADH dehydrogenase [ubiquinone] 1 alpha subcomplex assembly factor 4	NDUFAF4	-1.03	0.47485	Yes
O95490	Adhesion G protein-coupled receptor L2	ADGRL2	-1.02	0.17995	No
O95721	Synaptosomal-associated protein 29	SNAP29	-1.02	0.33875	Yes
O75531	Barrier-to-autointegration factor	BANF1	-1.01	0.42396	No
O75367	Core histone macro-H2A.1	H2AFY	-1.01	0.25323	No
Q9NRG7	Epimerase family protein SDR39U1	SDR39U1	-1.01	0.16886	Yes
Q9NV70	Exocyst complex component 1	EXOC1	-1.01	0.28233	No
Q8N1F7	Nuclear pore complex protein Nup93	NUP93	-1.01	0.32562	No
P20340	Ras-related protein Rab-6A	RAB6A	-1.01	0.43136	No
P08240	Signal recognition particle receptor subunit alpha	SRPRA	-1.01	0.25789	No
Q15370	Elongin-B	ELOB	-0.99	0.11644	No
O96008	Mitochondrial import receptor subunit TOM40 homolog	TOMM40	-0.99	0.27377	Yes
Q9Y289	Sodium-dependent multivitamin transporter	SLC5A6	-0.99	0.19251	No
P68032	Actin, alpha cardiac muscle 1	ACTC1	-0.98	0.33038	No
Q02978	Mitochondrial 2-oxoglutarate/malate carrier protein	SLC25A11	-0.98	0.13882	Yes
Q9UIW2	Plexin-A1	PLXNA1	-0.98	0.25319	No

P60604	Ubiquitin-conjugating enzyme E2 G2	UBE2G2	-0.98	0.22575	No
P09543	2',3'-cyclic-nucleotide 3'-phosphodiesterase	CNP	-0.97	0.50957	No
Q9Y2Q9	28S ribosomal protein S28, mitochondrial (bS1m)	MRPS28	-0.97	0.09292	Yes
A0A0A0MRA8	Band 4.1-like protein 3	EPB41L3	-0.97	0.15386	No
P62826	GTP-binding nuclear protein Ran	RAN	-0.97	0.44529	No
P13473	Lysosome-associated membrane glycoprotein 2	LAMP2	-0.97	0.32629	No
Q16718	NADH dehydrogenase [ubiquinone] 1 alpha subcomplex subunit 5	NDUFA5	-0.97	0.11769	Yes
Q15031	Probable leucine--tRNA ligase, mitochondrial	LARS2	-0.97	0.15385	Yes
Q6DKK2	Tetratricopeptide repeat protein 19, mitochondrial	TTC19	-0.97	0.30248	Yes
P82664	28S ribosomal protein S10, mitochondrial (uS10m)	MRPS10	-0.96	0.09123	Yes
P07900	Heat shock protein HSP 90-alpha	HSP90AA1	-0.96	0.18686	No
Q86UP2	Kinectin	KTN1	-0.96	0.58035	No
O94776	Metastasis-associated protein MTA2	MTA2	-0.96	0.51465	No
Q9H6H4	Receptor expression-enhancing protein 4	REEP4	-0.96	0.35518	No
Q9NX95	Syntabulin	SYBU	-0.96	0.13314	No
Q07065	Cytoskeleton-associated protein 4	CKAP4	-0.95	0.34755	No
P16422	Epithelial cell adhesion molecule	EPCAM	-0.95	0.3949	No
Q1KMD3	Heterogeneous nuclear ribonucleoprotein U-like protein 2	HNRNPUL2	-0.95	0.39039	No
Q5T440	Putative transferase CAF17, mitochondrial	IBA57	-0.95	0.07491	Yes
P50990	T-complex protein 1 subunit theta	CCT8	-0.95	0.30224	No
Q15543	Transcription initiation factor TFIID subunit 13	TAF13	-0.95	0.20861	No
Q96ER9	Coiled-coil domain-containing protein 51	CCDC51	-0.94	0.46228	Yes
P55789	FAD-linked sulfhydryl oxidase ALR	GFER	-0.94	0.38294	Yes
Q8TBA6	Golgin subfamily A member 5	GOLGA5	-0.94	0.1345	No
Q8WUK0	Phosphatidylglycerophosphatase and protein-tyrosine phosphatase 1	PTPMT1	-0.94	0.20632	Yes
Q9HA92	Radical S-adenosyl methionine domain-containing protein 1, mitochondrial	RSAD1	-0.94	0.18474	Yes
O15269	Serine palmitoyltransferase 1	SPTLC1	-0.94	0.256	No
Q03518	Antigen peptide transporter 1	TAP1	-0.93	0.3132	No
O60884	DnaJ homolog subfamily A member 2	DNAJA2	-0.93	0.42069	No
Q9H9P8	L-2-hydroxyglutarate dehydrogenase, mitochondrial	L2HGDH	-0.93	0.35633	Yes
P62316	Small nuclear ribonucleoprotein Sm D2	SNRPD2	-0.93	0.14058	No
Q5ST30	Valine--tRNA ligase, mitochondrial	VARS2	-0.93	0.32788	Yes
O75947	ATP synthase subunit d, mitochondrial	ATP5H	-0.92	0.12181	Yes
Q92538	Golgi-specific brefeldin A-resistance guanine nucleotide exchange factor 1	GBF1	-0.92	0.33484	No
Q6P4Q7	Metal transporter CNNM4	CNNM4	-0.92	0.22547	No
Q9NX63	MICOS complex subunit MIC19	CHCHD3	-0.92	0.19374	Yes

O95202	Mitochondrial proton/calcium exchanger protein	LETM1	-0.92	0.2636	Yes
O15321	Transmembrane 9 superfamily member 1	TM9SF1	-0.92	0.23155	No
Q9Y673	Dolichyl-phosphate beta-glucosyltransferase	ALG5	-0.91	0.34755	No
Q8NCW6	Polypeptide N-acetylgalactosaminyltransferase 11	GALNT11	-0.91	0.23417	No
Q96KR1	Zinc finger RNA-binding protein	ZFR	-0.91	0.40065	No
P39656	Dolichyl-diphosphooligosaccharide--protein glycosyltransferase 48 kDa subunit	DDOST	-0.9	0.36026	No
P78371	T-complex protein 1 subunit beta	CCT2	-0.9	0.30267	No
P55795	Heterogeneous nuclear ribonucleoprotein H2	HNRNPH2	-0.89	0.35155	No
Q8N8S7	Protein enabled homolog	ENAH	-0.89	0.26122	No
P17858	ATP-dependent 6-phosphofructokinase, liver type	PFKL	-0.88	0.20472	No
P54886	Delta-1-pyrroline-5-carboxylate synthase	ALDH18A1	-0.88	0.14058	Yes
O15118	NPC intracellular cholesterol transporter 1	NPC1	-0.88	0.51098	No
Q9BUP3	Oxidoreductase HTATIP2	HTATIP2	-0.88	0.2781	Yes
P37802	Transgelin-2	TAGLN2	-0.88	0.49276	No
Q12767	Transmembrane protein 94	TMEM94	-0.88	0.57885	No
Q9Y3D7	Mitochondrial import inner membrane translocase subunit TIM16	PAM16	-0.87	0.50602	Yes
Q15758	Neutral amino acid transporter B(0)	SLC1A5	-0.87	0.61899	No
P48643	T-complex protein 1 subunit epsilon	CCT5	-0.87	0.3132	No
O00461	Golgi integral membrane protein 4	GOLIM4	-0.86	0.32108	No
Q13547	Histone deacetylase 1	HDAC1	-0.86	0.48426	No
Q9Y3L5	Ras-related protein Rap-2c	RAP2C	-0.86	0.27596	No
Q92665	28S ribosomal protein S31, mitochondrial (mS31)	MRPS31	-0.85	0.16199	Yes
P24539	ATP synthase F(0) complex subunit B1, mitochondrial	ATP5F1	-0.85	0.2274	Yes
Q96RP9	Elongation factor G, mitochondrial	GFM1	-0.85	0.30444	Yes
Q96EE3	Nucleoporin SEH1	SEH1L	-0.85	0.23092	No
Q9UL25	Ras-related protein Rab-21	RAB21	-0.85	0.22933	No
P60602	Reactive oxygen species modulator 1	ROMO1	-0.85	0.28648	Yes
O75964	ATP synthase subunit g, mitochondrial	ATP5L	-0.84	0.17771	Yes
O15320	Endoplasmic reticulum export factor CTAGE5	CTAGE5	-0.84	0.33795	No
P49006	MARCKS-related protein	MARCKSL1	-0.84	0.41321	No
Q96RQ3	Methylcrotonoyl-CoA carboxylase subunit alpha, mitochondrial	MCCC1	-0.84	0.33467	Yes
O00268	Transcription initiation factor TFIID subunit 4	TAF4	-0.84	0.3132	No
Q9Y2Z4	Tyrosine--tRNA ligase, mitochondrial	YARS2	-0.84	0.289	Yes
O60264	SWI/SNF-related matrix-associated actin-dependent regulator of chromatin subfamily A member 5	SMARCA5	-0.83	0.32161	No
Q96E29	Transcription termination factor 3, mitochondrial	MTERF3	-0.83	0.33832	Yes

Q9GZP9	Derlin-2	DERL2	-0.82	0.36212	No
P22830	Ferrochelatase, mitochondrial	FECH	-0.82	0.47972	Yes
O95573	Long-chain-fatty-acid--CoA ligase 3	ACSL3	-0.82	0.53801	No
Q16891	MICOS complex subunit MIC60	IMMT	-0.82	0.44863	Yes
O43615	Mitochondrial import inner membrane translocase subunit TIM44	TIMM44	-0.82	0.26179	Yes
Q9NS69	Mitochondrial import receptor subunit TOM22 homolog	TOMM22	-0.82	0.55269	Yes
Q99959	Plakophilin-2	PKP2	-0.82	0.22575	No
F8VZX2	Poly(rC)-binding protein 2	PCBP2	-0.82	0.35455	No
O95347	Structural maintenance of chromosomes protein 2	SMC2	-0.82	0.19324	No
Q86YP4	Transcriptional repressor p66-alpha	GATAD2A	-0.82	0.38957	No
P51571	Translocon-associated protein subunit delta	SSR4	-0.82	0.52123	No
Q9HDC9	Adipocyte plasma membrane-associated protein	APMAP	-0.81	0.56681	No
Q9Y4W6	AFG3-like protein 2	AFG3L2	-0.81	0.40763	Yes
G3V325	ATP5MF-PTCD1 readthrough	ATP5MF-PTCD1	-0.81	0.28194	No
Q96SZ6	CDK5 regulatory subunit-associated protein 1	CDK5RAP1	-0.81	0.41061	No
O75251	NADH dehydrogenase [ubiquinone] iron-sulfur protein 7, mitochondrial	NDUFS7	-0.8	0.50645	Yes
Q9BQ75	Protein CMSS1	CMSS1	-0.8	0.32629	No
Q9Y2G0	Protein EFR3 homolog B	EFR3B	-0.8	0.35166	No
Q15061	WD repeat-containing protein 43	WDR43	-0.8	0.41009	No
Q6ULP2	Aftiphilin	AFTPH	-0.79	0.33795	No
P04792	Heat shock protein beta-1	HSPB1	-0.79	0.23939	No
P02545	Prelamin-A/C	LMNA	-0.79	0.36982	No
P07437	Tubulin beta chain	TUBB	-0.79	0.17984	No
O14734	Acyl-coenzyme A thioesterase 8	ACOT8	-0.78	0.23242	No
Q9BRR6	ADP-dependent glucokinase	ADPGK	-0.78	0.53451	No
A0A087WTF6	Neural cell adhesion molecule 1	NCAM1	-0.78	0.37491	No
P05166	Propionyl-CoA carboxylase beta chain, mitochondrial	PCCB	-0.78	0.43047	Yes
Q6P158	Putative ATP-dependent RNA helicase DHX57	DHX57	-0.78	0.40065	No
Q9UGP8	Translocation protein SEC63 homolog	SEC63	-0.78	0.50318	No
Q9H019	Mitochondrial fission regulator 1-like	MTFR1L	-0.77	0.17901	Yes
Q8IXI2	Mitochondrial Rho GTPase 1	RHOT1	-0.77	0.34248	Yes
P28331	NADH-ubiquinone oxidoreductase 75 kDa subunit, mitochondrial	NDUFS1	-0.77	0.27666	Yes
O75688	Protein phosphatase 1B	PPM1B	-0.77	0.43453	No
Q9UJZ1	Stomatin-like protein 2, mitochondrial	STOML2	-0.77	0.27377	Yes
P61769	Beta-2-microglobulin	B2M	-0.76	0.56681	No

P49792	E3 SUMO-protein ligase RanBP2	RANBP2	-0.76	0.40914	No
Q08945	FACT complex subunit SSRP1	SSRP1	-0.76	0.41009	No
Q8NBN7	Retinol dehydrogenase 13	RDH13	-0.76	0.25862	Yes
Q7L0J3	Synaptic vesicle glycoprotein 2A	SV2A	-0.76	0.54174	No
Q9Y5Z9	UbiA prenyltransferase domain-containing protein 1	UBIAD1	-0.76	0.33281	No
Q13155	Aminoacyl tRNA synthase complex-interacting multifunctional protein 2	AIMP2	-0.75	0.55673	No
Q8NE71	ATP-binding cassette sub-family F member 1	ABCF1	-0.75	0.56975	No
Q8N766	ER membrane protein complex subunit 1	EMC1	-0.75	0.39988	No
Q5JPE7	Nodal modulator 2	NOMO2	-0.75	0.55245	No
P46199	Translation initiation factor IF-2, mitochondrial	MTIF2	-0.75	0.62734	Yes
Q9Y3D5	28S ribosomal protein S18c, mitochondrial (bS18m)	MRPS18C	-0.74	0.24517	Yes
Q96D53	Atypical kinase COQ8B, mitochondrial	COQ8B	-0.74	0.39689	Yes
P43897	Elongation factor Ts, mitochondrial	TSFM	-0.74	0.33484	Yes
Q96I24	Far upstream element-binding protein 3	FUBP3	-0.73	0.58809	No
Q8N4H5	Mitochondrial import receptor subunit TOM5 homolog	TOMM5	-0.73	0.45412	Yes
Q8NC60	Nitric oxide-associated protein 1	NOA1	-0.73	0.3132	Yes
Q14683	Structural maintenance of chromosomes protein 1A	SMC1A	-0.73	0.42602	No
Q9UEG4	Zinc finger protein 629	ZNF629	-0.73	0.45061	No
P55265	Double-stranded RNA-specific adenosine deaminase	ADAR	-0.72	0.35863	No
Q13505	Metaxin-1	MTX1	-0.72	0.66942	Yes
Q9H4K7	Mitochondrial ribosome-associated GTPase 2	MTG2	-0.72	0.33875	Yes
Q13613	Myotubularin-related protein 1	MTMR1	-0.72	0.25512	No
Q8WXF7	Atlastin-1	ATL1	-0.71	0.42341	No
A0A2R8Y60 2	Caseinolytic peptidase B protein homolog (Fragment)	CLPB	-0.71	0.51225	Yes
O75521	Enoyl-CoA delta isomerase 2, mitochondrial	ECI2	-0.71	0.48333	Yes
Q53GS7	Nucleoporin GLE1	GLE1	-0.71	0.3949	No
Q99623	Prohibitin-2	PHB2	-0.71	0.25133	Yes
Q9NRX1	RNA-binding protein PNO1	PNO1	-0.71	0.38908	No
O75533	Splicing factor 3B subunit 1	SF3B1	-0.71	0.51417	No
Q6NUQ4	Transmembrane protein 214	TMEM214	-0.71	0.53515	No
Q8TDB4	Protein MGARP	MGARP	-0.7	0.33795	Yes
Q9BVC6	Transmembrane protein 109	TMEM109	-0.7	0.40212	No
P12236	ADP/ATP translocase 3	SLC25A6	-0.69	0.25319	Yes
P07339	Cathepsin D	CTSD	-0.69	0.49276	No
Q9C005	Protein dpy-30 homolog	DPY30	-0.69	0.31931	No
Q9UKM9	RNA-binding protein Raly	RALY	-0.69	0.5521	No

Q8N1F8	Serine/threonine-protein kinase 11-interacting protein	STK11IP	-0.69	0.2781	No
Q96G23	Ceramide synthase 2	CERS2	-0.68	0.42738	No
P01889	HLA class I histocompatibility antigen, B-7 alpha chain	HLA-B	-0.68	0.52417	No
O75781	Paralemmin-1	PALM	-0.68	0.3932	No
P62314	Small nuclear ribonucleoprotein Sm D1	SNRPD1	-0.68	0.31078	No
Q12907	Vesicular integral-membrane protein VIP36	LMAN2	-0.68	0.5461	No
Q3SXY8	ADP-ribosylation factor-like protein 13B	ARL13B	-0.67	0.47083	No
P00167	Cytochrome b5	CYB5A	-0.67	0.49276	Yes
Q6ZNB6	NF-X1-type zinc finger protein NFXL1	NFXL1	-0.67	0.53801	No
P61619	Protein transport protein Sec61 subunit alpha isoform 1	SEC61A1	-0.67	0.46703	No
P61019	Ras-related protein Rab-2A	RAB2A	-0.67	0.55476	No
Q9BWM7	Sideroflexin-3	SFXN3	-0.67	0.60463	Yes
O75844	CAAX prenyl protease 1 homolog	ZMPSTE24	-0.66	0.35532	No
P51648	Fatty aldehyde dehydrogenase	ALDH3A2	-0.66	0.65335	Yes
Q9Y3D0	Mitotic spindle-associated MMXD complex subunit MIP18	FAM96B	-0.66	0.40213	No
Q9Y2W6	Tudor and KH domain-containing protein	TDRKH	-0.66	0.51445	Yes
P06733	Alpha-enolase	ENO1	-0.65	0.67149	No
O75489	NADH dehydrogenase [ubiquinone] iron-sulfur protein 3, mitochondrial	NDUFS3	-0.65	0.30824	Yes
Q8NBU5	ATPase family AAA domain-containing protein 1	ATAD1	-0.64	0.55245	Yes
O00186	Syntaxin-binding protein 3	STXBP3	-0.64	0.46519	No
Q9Y6K0	Choline/ethanolaminephosphotransferase 1	CEPT1	-0.63	0.48988	No
Q9HAV7	GrpE protein homolog 1, mitochondrial	GRPEL1	-0.63	0.33979	Yes
Q5T2T1	MAGUK p55 subfamily member 7	MPP7	-0.63	0.49158	No
Q9Y639	Neuroplastin	NPTN	-0.63	0.49934	No
Q9NTK5	Obg-like ATPase 1	OLA1	-0.63	0.53238	No
Q9P2R7	Succinate--CoA ligase [ADP-forming] subunit beta, mitochondrial	SUCLA2	-0.63	0.44248	Yes
Q00587	Cdc42 effector protein 1	CDC42EP1	-0.62	0.39421	No
P06730	Eukaryotic translation initiation factor 4E	EIF4E	-0.62	0.60789	No
Q8NHZ8	Anaphase-promoting complex subunit CDC26	CDC26	-0.61	0.4962	No
Q99942	E3 ubiquitin-protein ligase RNF5	RNF5	-0.61	0.51219	No
Q9NPE2	Neugrin	NGRN	-0.61	0.48333	Yes
Q5SWX8	Protein odr-4 homolog	ODR4	-0.61	0.44764	No
O43504	Ragulator complex protein LAMTOR5	LAMTOR5	-0.61	0.37927	No
Q969Y2	tRNA modification GTPase GTPBP3, mitochondrial	GTPBP3	-0.61	0.45357	Yes
P26640	Valine--tRNA ligase	VARS	-0.61	0.54243	No
P33121	Long-chain-fatty-acid--CoA ligase 1	ACSL1	-0.6	0.59204	Yes

Q16637	Survival motor neuron protein	SMN1; SMN2	-0.6	0.42268	No
Q13425	Beta-2-syntrophin	SNTB2	-0.59	0.36212	No
O15400	Syntaxin-7	STX7	-0.59	0.57717	No
Q9Y282	Endoplasmic reticulum-Golgi intermediate compartment protein 3	ERGIC3	-0.58	0.57709	No
Q16576	Histone-binding protein RBBP7	RBBP7	-0.58	0.52725	No
P22033	Methylmalonyl-CoA mutase, mitochondrial	MUT	-0.58	0.5461	Yes
Q8TDY2	RB1-inducible coiled-coil protein 1	RB1CC1	-0.57	0.45733	No
Q96BD8	Spindle and kinetochore-associated protein 1	SKA1	-0.57	0.35353	No
P56962	Syntaxin-17	STX17	-0.57	0.53801	Yes
P20674	Cytochrome c oxidase subunit 5A, mitochondrial	COX5A	-0.56	0.39988	Yes
Q49AM1	Transcription termination factor 2, mitochondrial	MTERF2	-0.56	0.63533	Yes
P82673	28S ribosomal protein S35, mitochondrial (mS35)	MRPS35	-0.55	0.37098	Yes
Q5U5X0	Complex III assembly factor LYRM7	LYRM7	-0.55	0.53864	Yes
P49366	Deoxyhypusine synthase	DHPS	-0.55	0.38679	No
Q9UQ35	Serine/arginine repetitive matrix protein 2	SRRM2	-0.55	0.64269	No
G3V4C1	Heterogeneous nuclear ribonucleoproteins C1/C2	HNRNPC	-0.54	0.39988	No
P56192	Methionine--tRNA ligase, cytoplasmic	MARS	-0.54	0.6906	No
Q86XZ4	Spermatogenesis-associated serine-rich protein 2	SPATS2	-0.54	0.60104	No
Q03923	Zinc finger protein 85	ZNF85	-0.54	0.50318	No
Q92520	Protein FAM3C	FAM3C	-0.53	0.62595	No
Q8N357	Solute carrier family 35 member F6	SLC35F6	-0.53	0.49511	No
Q9Y291	28S ribosomal protein S33, mitochondrial (mS33)	MRPS33	-0.52	0.41532	Yes
P35222	Catenin beta-1	CTNNB1	-0.52	0.69814	No
Q04637	Eukaryotic translation initiation factor 4 gamma 1	EIF4G1	-0.52	0.65293	No
O43390	Heterogeneous nuclear ribonucleoprotein R	HNRNPR	-0.52	0.43317	No
P54709	Sodium/potassium-transporting ATPase subunit beta-3	ATP1B3	-0.52	0.72701	No
Q6PI78	Transmembrane protein 65	TMEM65	-0.52	0.53777	Yes
P05141	ADP/ATP translocase 2	SLC25A5	-0.5	0.42069	Yes
Q6YN16	Hydroxysteroid dehydrogenase-like protein 2	HSDL2	-0.5	0.51021	Yes
O14974	Protein phosphatase 1 regulatory subunit 12A	PPP1R12A	-0.5	0.50645	No
P35241	Radixin	RDX	-0.5	0.65245	No
O14874	[3-methyl-2-oxobutanoate dehydrogenase [lipoamide]] kinase, mitochondrial	BCKDK	-0.49	0.60105	Yes
P22695	Cytochrome b-c1 complex subunit 2, mitochondrial	UQCRC2	-0.49	0.38679	Yes
Q12931	Heat shock protein 75 kDa, mitochondrial	TRAP1	-0.49	0.70783	Yes
Q8N5C6	S1 RNA-binding domain-containing protein 1	SRBD1	-0.49	0.53469	No

P45954	Short/branched chain specific acyl-CoA dehydrogenase, mitochondrial	ACADSB	-0.49	0.67773	Yes
Q9NSE4	Isoleucine--tRNA ligase, mitochondrial	IARS2	-0.48	0.51417	Yes
P50991	T-complex protein 1 subunit delta	CCT4	-0.48	0.54115	No
P63010	AP-2 complex subunit beta	AP2B1	-0.47	0.53935	No
Q29963	HLA class I histocompatibility antigen, Cw-6 alpha chain	HLA-C	-0.47	0.55861	No
Q8TB37	Iron-sulfur protein NUBPL	NUBPL	-0.47	0.60394	Yes
P61006	Ras-related protein Rab-8A	RAB8A	-0.47	0.58679	No
P43304	Glycerol-3-phosphate dehydrogenase, mitochondrial	GPD2	-0.45	0.66596	Yes
Q5T160	Probable arginine--tRNA ligase, mitochondrial	RARS2	-0.45	0.53451	Yes
O43819	Protein SCO2 homolog, mitochondrial	SCO2	-0.45	0.65157	Yes
P11233	Ras-related protein Ral-A	RALA	-0.45	0.53238	No
P68363	Tubulin alpha-1B chain	TUBA1B	-0.45	0.48928	No
P31689	DnaJ homolog subfamily A member 1	DNAJA1	-0.44	0.60886	No
F8VVM2	Phosphate carrier protein, mitochondrial	SLC25A3	-0.44	0.74006	Yes
P55011	Solute carrier family 12 member 2	SLC12A2	-0.44	0.65636	No
O43169	Cytochrome b5 type B	CYB5B	-0.43	0.66043	Yes
Q96EY7	Pentatricopeptide repeat domain-containing protein 3, mitochondrial	PTCD3	-0.43	0.50957	Yes
Q9P289	Serine/threonine-protein kinase 26	STK26	-0.43	0.67867	No
O94826	Mitochondrial import receptor subunit TOM70	TOMM70	-0.42	0.62734	Yes
Q9HCU5	Prolactin regulatory element-binding protein	PREB	-0.42	0.73911	No
P68371	Tubulin beta-4B chain	TUBB4B	-0.42	0.53887	No
P23246	Splicing factor, proline- and glutamine-rich	SFPQ	-0.41	0.68993	No
P82932	28S ribosomal protein S6, mitochondrial (bS6m)	MRPS6	-0.4	0.43987	Yes
Q7Z4W1	L-xylulose reductase	DCXR	-0.4	0.54786	Yes
Q00325	Phosphate carrier protein, mitochondrial	SLC25A3	-0.4	0.57612	Yes
Q96ND0	Protein FAM210A	FAM210A	-0.4	0.60494	Yes
Q8WUY1	Protein THEM6	THEM6	-0.4	0.50001	No
J3QSX6	Actin-binding LIM protein 1	ABLIM1	-0.39	0.7519	No
O15382	Branched-chain-amino-acid aminotransferase, mitochondrial	BCAT2	-0.39	0.60285	Yes
Q96CS3	FAS-associated factor 2	FAF2	-0.39	0.58888	No
O75431	Metaxin-2	MTX2	-0.39	0.68028	Yes
Q9BQT8	Mitochondrial 2-oxodicarboxylate carrier	SLC25A21	-0.39	0.6803	Yes
P13489	Ribonuclease inhibitor	RNH1	-0.39	0.74884	No
Q9H0U9	Testis-specific Y-encoded-like protein 1	TSPYL1	-0.39	0.60789	No
Q9BQ95	Evolutionarily conserved signaling intermediate in Toll pathway, mitochondrial	ECSIT	-0.38	0.79068	Yes
Q92552	28S ribosomal protein S27, mitochondrial (mS27)	MRPS27	-0.37	0.57612	Yes

O95816	BAG family molecular chaperone regulator 2	BAG2	-0.37	0.6419	No
P58557	Endoribonuclease YbeY	YBEY	-0.37	0.74251	Yes
P05556	Integrin beta-1	ITGB1	-0.37	0.80278	No
Q9P2B2	Prostaglandin F2 receptor negative regulator	PTGFRN	-0.37	0.80709	No
O43824	Putative GTP-binding protein 6	GTPBP6	-0.37	0.71206	Yes
P62820	Ras-related protein Rab-1A	RAB1A	-0.37	0.76646	No
Q9BTC8	Metastasis-associated protein MTA3	MTA3	-0.36	0.75105	No
Q15836	Vesicle-associated membrane protein 3	VAMP3	-0.36	0.74814	No
A1L0T0	Acetolactate synthase-like protein	ILVBL	-0.35	0.63965	No
Q96DA6	Mitochondrial import inner membrane translocase subunit TIM14	DNAJC19	-0.35	0.79738	Yes
Q9UQ90	Paraplegin	SPG7	-0.35	0.67881	Yes
P13073	Cytochrome c oxidase subunit 4 isoform 1, mitochondrial	COX4I1	-0.34	0.60035	Yes
Q7L592	Protein arginine methyltransferase NDUFAF7, mitochondrial	NDUFAF7	-0.34	0.74804	Yes
P21926	CD9 antigen	CD9	-0.33	0.77342	No
P04439	HLA class I histocompatibility antigen, A-3 alpha chain	HLA-A	-0.33	0.8294	No
O95168	NADH dehydrogenase [ubiquinone] 1 beta subcomplex subunit 4	NDUFB4	-0.33	0.75984	Yes
P51659	Peroxisomal multifunctional enzyme type 2	HSD17B4	-0.33	0.60002	Yes
Q9P2W9	Syntaxin-18	STX18	-0.33	0.65526	No
Q13472	DNA topoisomerase 3-alpha	TOP3A	-0.32	0.65636	Yes
O43676	NADH dehydrogenase [ubiquinone] 1 beta subcomplex subunit 3	NDUFB3	-0.32	0.77809	Yes
Q8NF37	Lysophosphatidylcholine acyltransferase 1	LPCAT1	-0.31	0.70442	No
Q99519	Sialidase-1	NEU1	-0.31	0.68554	No
P51572	B-cell receptor-associated protein 31	BCAP31	-0.3	0.72627	No
P34897	Serine hydroxymethyltransferase, mitochondrial	SHMT2	-0.3	0.64158	Yes
Q9NP81	Serine--tRNA ligase, mitochondrial	SARS2	-0.3	0.63922	Yes
P62304	Small nuclear ribonucleoprotein E	SNRPE	-0.3	0.81264	No
Q96GE9	Distal membrane-arm assembly complex protein 1	DMAC1	-0.28	0.81817	No
Q7L8L6	FAST kinase domain-containing protein 5, mitochondrial	FASTKD5	-0.28	0.73644	Yes
Q9Y266	Nuclear migration protein nudC	NUDC	-0.28	0.77758	No
Q9BZI7	Regulator of nonsense transcripts 3B	UPF3B	-0.28	0.74006	No
Q9BWF3	RNA-binding protein 4	RBM4	-0.28	0.81817	No
Q9P035	Very-long-chain (3R)-3-hydroxyacyl-CoA dehydratase 3	HACD3	-0.28	0.73362	No
Q96TA2	ATP-dependent zinc metalloprotease YME1L1	YME1L1	-0.27	0.83989	Yes
P41091	Eukaryotic translation initiation factor 2 subunit 3	EIF2S3	-0.27	0.82874	No
Q06265	Exosome complex component RRP45	EXOSC9	-0.27	0.81817	No
P04075	Fructose-bisphosphate aldolase A	ALDOA	-0.27	0.87649	No
Q15738	Sterol-4-alpha-carboxylate 3-dehydrogenase, decarboxylating	NSDHL	-0.27	0.80856	No

Q9BV40	Vesicle-associated membrane protein 8	VAMP8	-0.27	0.7738	No
Q9Y5M8	Signal recognition particle receptor subunit beta	SRPRB	-0.26	0.77949	No
Q9BY67	Cell adhesion molecule 1	CADM1	-0.25	0.89081	No
P63241	Eukaryotic translation initiation factor 5A-1	EIF5A	-0.25	0.81264	No
Q01804	OTU domain-containing protein 4	OTUD4	-0.25	0.88792	No
Q96TC7	Regulator of microtubule dynamics protein 3	RMDN3	-0.25	0.79209	Yes
P03928	ATP synthase protein 8	MT-ATP8	-0.24	0.72014	Yes
Q5T3I0	G patch domain-containing protein 4	GPATCH4	-0.24	0.74811	No
Q5JRX3	Presequence protease, mitochondrial	PITRM1	-0.23	0.78227	Yes
P14406	Cytochrome c oxidase subunit 7A2, mitochondrial	COX7A2	-0.22	0.80944	Yes
Q9NRG9	Aladin	AAAS	-0.21	0.82874	No
Q16352	Alpha-internexin	INA	-0.21	0.82874	No
P27824	Calnexin	CANX	-0.21	0.78738	No
P98172	Ephrin-B1	EFNB1	-0.21	0.8332	No
P35240	Merlin	NF2	-0.21	0.8644	No
P49757	Protein numb homolog	NUMB	-0.21	0.80944	No
Q9H4I3	TraB domain-containing protein	TRABD	-0.21	0.86443	No
Q8NI36	WD repeat-containing protein 36	WDR36	-0.21	0.80602	No
P68104	Elongation factor 1-alpha 1	EEF1A1	-0.2	0.82067	No
O95249	Golgi SNAP receptor complex member 1	GOSR1	-0.2	0.8247	No
Q1ED39	Lysine-rich nucleolar protein 1	KNOP1	-0.2	0.81817	No
Q10471	Polypeptide N-acetylgalactosaminyltransferase 2	GALNT2	-0.2	0.86638	No
P53621	Coatomer subunit alpha	COPA	-0.18	0.85332	No
O95232	Luc7-like protein 3	LUC7L3	-0.18	0.86195	No
O00264	Membrane-associated progesterone receptor component 1	PGRMC1	-0.18	0.85036	No
Q13330	Metastasis-associated protein MTA1	MTA1	-0.18	0.88909	No
O95299	NADH dehydrogenase [ubiquinone] 1 alpha subcomplex subunit 10, mitochondrial	NDUFA10	-0.18	0.8688	Yes
P22626	Heterogeneous nuclear ribonucleoproteins A2/B1	HNRNPA2B1	-0.17	0.81866	No
Q92769	Histone deacetylase 2	HDAC2	-0.17	0.8988	No
P35813	Protein phosphatase 1A	PPM1A	-0.17	0.85347	No
P14678	Small nuclear ribonucleoprotein-associated proteins B and B'	SNRPB	-0.17	0.7519	No
P16989	Y-box-binding protein 3	YBX3	-0.17	0.82067	No
Q8N4T8	Carbonyl reductase family member 4	CBR4	-0.15	0.8918	Yes
Q53GQ0	Very-long-chain 3-oxoacyl-CoA reductase	HSD17B12	-0.15	0.92282	No
Q9GZY8	Mitochondrial fission factor	MFF	-0.14	0.87174	Yes
Q3ZCQ8	Mitochondrial import inner membrane translocase subunit TIM50	TIMM50	-0.13	0.87137	Yes

Q9NP58	ATP-binding cassette sub-family B member 6, mitochondrial	ABCB6	-0.12	0.93121	Yes
Q9NVJ2	ADP-ribosylation factor-like protein 8B	ARL8B	-0.11	0.91268	No
Q13740	CD166 antigen	ALCAM	-0.11	0.8978	No
P0C7P0	CDGSH iron-sulfur domain-containing protein 3, mitochondrial	CISD3	-0.11	0.93073	Yes
O15091	Mitochondrial ribonuclease P catalytic subunit	KIAA0391	-0.11	0.90168	Yes
Q9Y676	28S ribosomal protein S18b, mitochondrial (mS40)	MRPS18B	-0.1	0.87949	Yes
Q9BRK5	45 kDa calcium-binding protein	SDF4	-0.1	0.94242	No
Q9HCM7	Fibrosin-1-like protein	FBRSL1	-0.1	0.93305	No
O14828	Secretory carrier-associated membrane protein 3	SCAMP3	-0.1	0.93373	No
Q9Y679	Ancient ubiquitous protein 1	AUP1	-0.09	0.93073	No
P07355	Annexin A2	ANXA2	-0.09	0.94364	No
Q7L5N7	Lysophosphatidylcholine acyltransferase 2	LPCAT2	-0.09	0.93073	No
Q9BVA1	Tubulin beta-2B chain	TUBB2B	-0.09	0.93073	No
Q9BRZ2	E3 ubiquitin-protein ligase TRIM56	TRIM56	-0.08	0.93936	No
Q92979	Ribosomal RNA small subunit methyltransferase NEP1	EMG1	-0.08	0.93073	No
Q00610	Clathrin heavy chain 1	CLTC	-0.07	0.95257	No
Q13409	Cytoplasmic dynein 1 intermediate chain 2	DYNC1I2	-0.07	0.96657	No
Q7KZF4	Staphylococcal nuclease domain-containing protein 1	SND1	-0.07	0.96911	Yes
Q9NUL3	Double-stranded RNA-binding protein Staufen homolog 2	STAU2	-0.06	0.9383	No
Q7Z4V5	Hepatoma-derived growth factor-related protein 2	HDGFL2	-0.06	0.97336	No
Q9P0U1	Mitochondrial import receptor subunit TOM7 homolog	TOMM7	-0.06	0.94458	Yes
O43143	Pre-mRNA-splicing factor ATP-dependent RNA helicase DHX15	DHX15	-0.06	0.97028	No
P23786	Carnitine O-palmitoyltransferase 2, mitochondrial	CPT2	-0.05	0.97028	Yes
P30084	Enoyl-CoA hydratase, mitochondrial	ECHS1	-0.05	0.97347	Yes
Q6UXV4	MICOS complex subunit MIC27	APOOL	-0.05	0.95027	Yes
Q5VT25	Serine/threonine-protein kinase MRCK alpha	CDC42BPA	-0.05	0.97336	No
Q9Y2R0	Cytochrome c oxidase assembly factor 3 homolog, mitochondrial	COA3	-0.04	0.97336	Yes
P49411	Elongation factor Tu, mitochondrial	TUFM	-0.04	0.95367	Yes
Q9BVK6	Transmembrane emp24 domain-containing protein 9	TMED9	-0.04	0.97336	No
O75396	Vesicle-trafficking protein SEC22b	SEC22B	-0.04	0.97336	No
O60831	PRA1 family protein 2	PRAF2	-0.03	0.97028	No
P60174	Triosephosphate isomerase	TPI1	-0.03	0.96911	Yes
Q8N5I2	Arrestin domain-containing protein 1	ARRDC1	-0.02	0.98312	No
Q9GZL7	Ribosome biogenesis protein WDR12	WDR12	-0.02	0.97439	No
Q8WVM8	Sec1 family domain-containing protein 1	SCFD1	-0.02	0.98242	No
Q92504	Zinc transporter SLC39A7	SLC39A7	-0.02	0.97995	No
Q5JTV8	Torsin-1A-interacting protein 1	TOR1AIP1	-0.01	0.9947	No

P42696	RNA-binding protein 34	RBM34	3	0.01055	No
Q9Y3B9	RRP15-like protein	RRP15	2	0.03433	No
P09234	U1 small nuclear ribonucleoprotein C	SNRPC	2	0.002	No
Q6ZN17	Protein lin-28 homolog B	LIN28B	1	0.14435	No
P08195	4F2 cell-surface antigen heavy chain	SLC3A2	0	0.99797	No
P32322	Pyrroline-5-carboxylate reductase 1, mitochondrial	PYCR1	0,00	0.99742	Yes
Q96EL2	28S ribosomal protein S24, mitochondrial (uS3m)	MRPS24	-1,00	0.15216	Yes
Q15365	Poly(rC)-binding protein 1	PCBP1	-1	0.27324	No
Q13948	Protein CASP	CUX1	-1	0.28444	No
P40937	Replication factor C subunit 5	RFC5	-1	0.21142	No
Q969X6	U3 small nucleolar RNA-associated protein 4 homolog	UTP4	-1	0.14705	No
O00487	26S proteasome non-ATPase regulatory subunit 14	PSMD14	-2	0.04302	No
Q9Y2H1	Serine/threonine-protein kinase 38-like	STK38L	-3	0.00421	No
Q99470	Stromal cell-derived factor 2	SDF2	-3	0.00698	No
Q4G0N4	NAD kinase 2, mitochondrial	NADK2	-5,00	0	Yes

Names of the mitochondrial ribosomal proteins according to the new nomenclature (as per reference 8) are mentioned in parentheses.
