# Supplementary material for: C6orf203 is an RNA-binding protein involved in mitochondrial protein synthesis
Source: Nucleic Acids Res. 2019 Aug 9;47(17):9386–99. doi: 10.1093/nar/gkz684 (PMC6755124; doi:10.1093/nar/gkz684)
Supplement: gkz684_Supplemental_Files [file nar_47_17_9386_s1.zip › Supplementary material -revision2207.pdf]

Supplementary information for

C6orf203 is an RNA-binding protein involved in mitochondrial protein synthesis

Shreekara Gopalakrishna, Sarah F. Pearce, Adam M. Dinan, Florian A. Schober, Miriam Cipullo, Henrik Spåhr, Anas Khawaja, Camilla Maffezzini, Christoph Freyer, Anna Wredenberg, Ilian Atanassov, Andrew E. Firth, Joanna Rorbach

SUPPLEMENTARY FIGURES

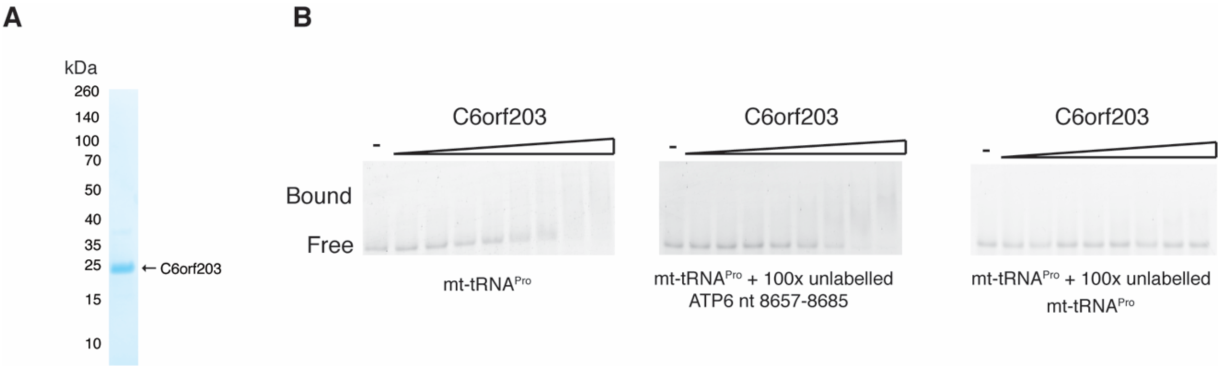

Supplementary Figure S1. RNA-binding activity of C6orf203. (A) SDS-PAGE indicating purity of recombinant human C6orf203 protein used for EMSA assays. **(B)** Competitive RNA electrophoretic mobility shift assay (EMSA). Recombinant human C6orf203 was incubated with fluorescein-labelled dsRNA from mt-$tRNA^{Pro}$ (mtDNA 15956-16023), and either no unlabeled RNA (left), unlabelled ssRNA (centre) or unlabelled dsRNA (right) at a concentration 100x greater (10 μM) than the fluorescein-labelled mt-$tRNA^{Pro}$. RNA template sequences used were as indicated (nt. position in mtDNA). Protein concentrations used were 0, 0.02, 0.04, 0.08, 0.16, 0.36, 0.64, 1.28, 2.56 μM, respectively.

A

B

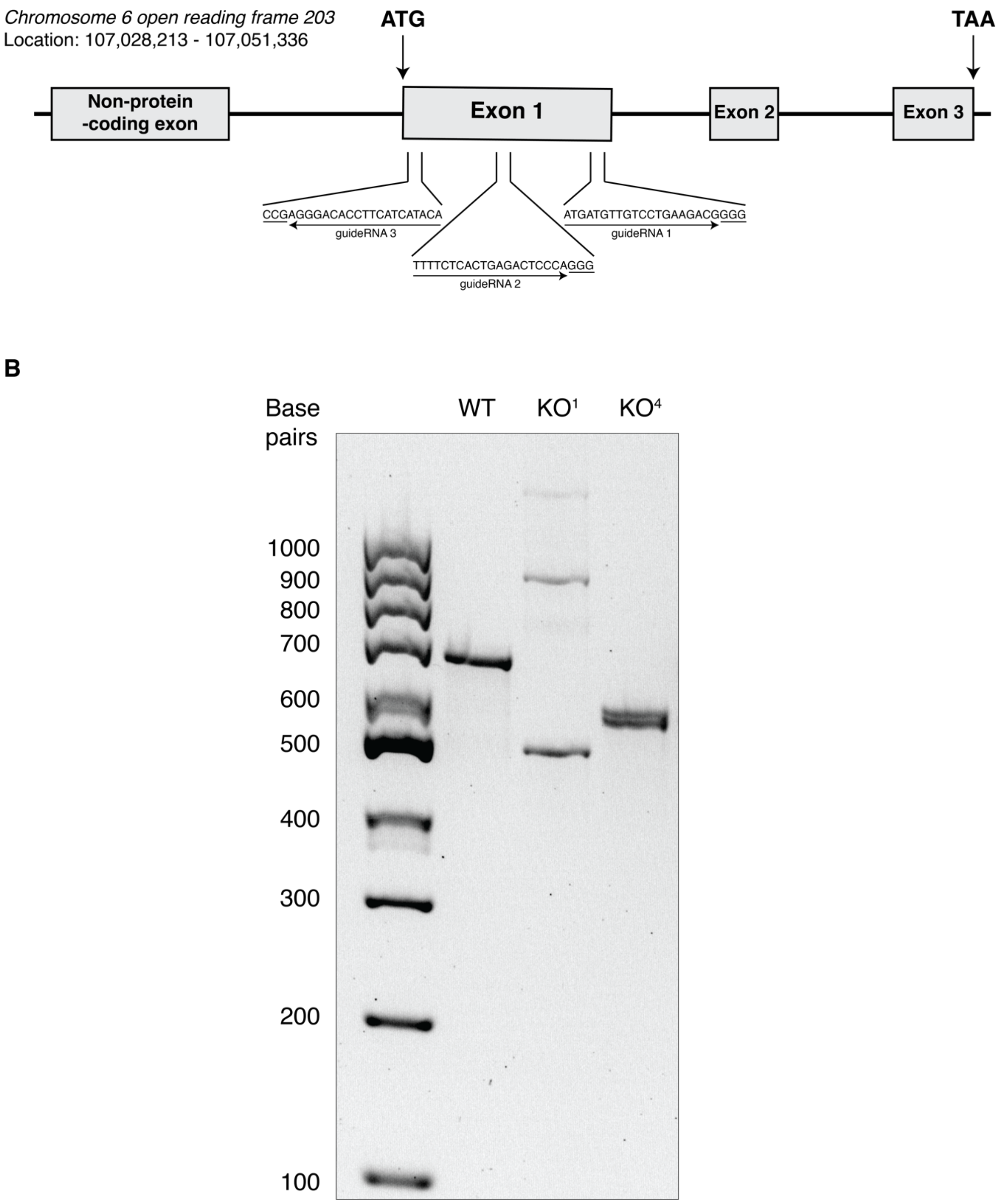

Supplementary Figure S2. Generation of C6orf203 knock-out clones. (A) Schematic representation of C6orf203 gene locus indicating target sites of guide RNAs within exon 1. Underlined sequences represent the PAM sites. **(B)** PCR of C6orf203 in WT and knockouts. C6orf203 KO[1] is a result of transfection of HEK293 with gRNA set 1 and 3, and C6orf203 KO[4] is a result of the gRNAs 2 and 3.

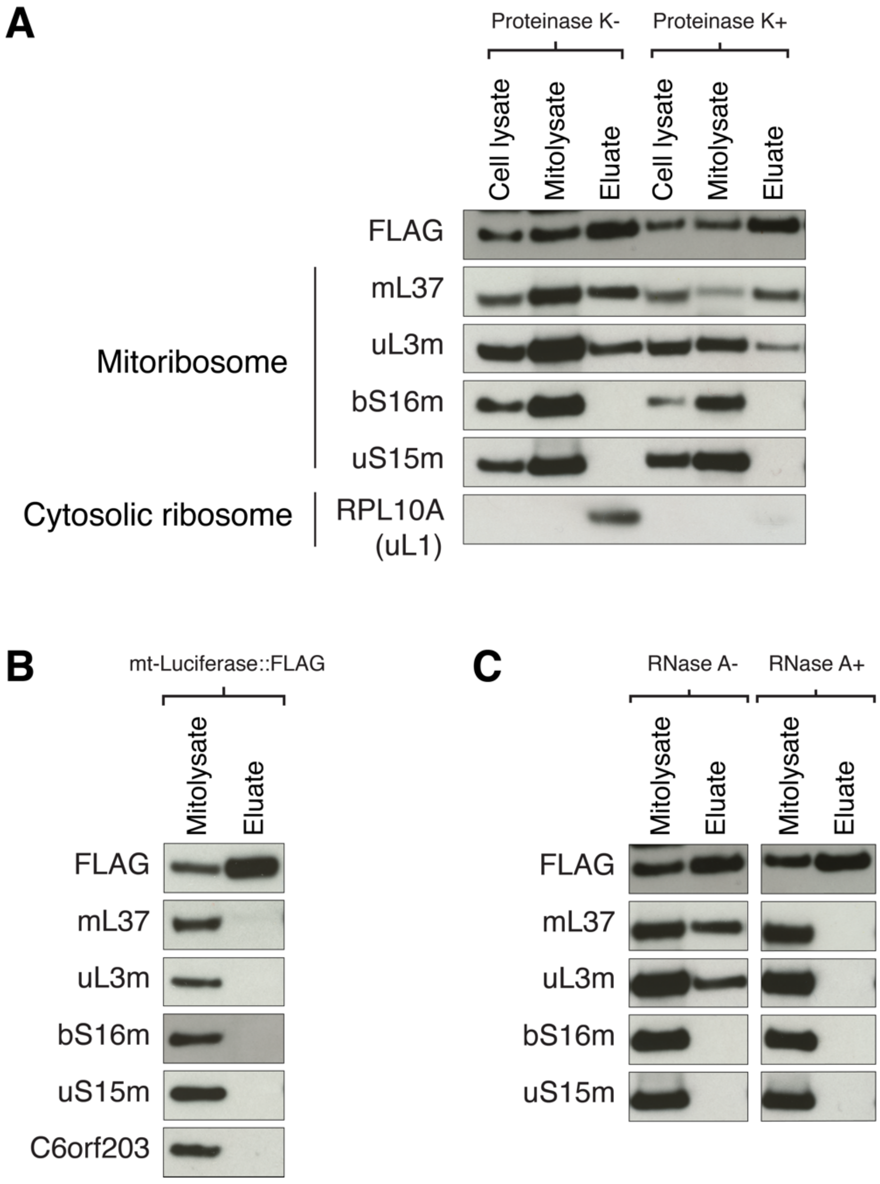

Supplementary Figure S3. Immunoprecipitation experiments to investigate nature of binding of C6orf203 to mitoribosomes. (A) FLAG-immunoprecipitation of C6orf203 was performed as described in Figure 5 of the main text, where mitochondria were treated with or without 20ug/ml Proteinase K prior to lysis (described in supplementary materials below). **(B)** FLAG-immunoprecipitation of mitochondrially targeted luciferase as a negative control for binding of mitoribosomal proteins. **(C)** FLAG-immunoprecipitation of C6orf203 was performed, where mitochondrial lysates were treated with or without RNase A (20ug/ml) following lysis prior to IP protocol. For all panels, resulting lysates and eluates were used for western blotting and the membranes were probed with antibodies specific to either proteins of the mitochondrial or cytosolic ribosome.

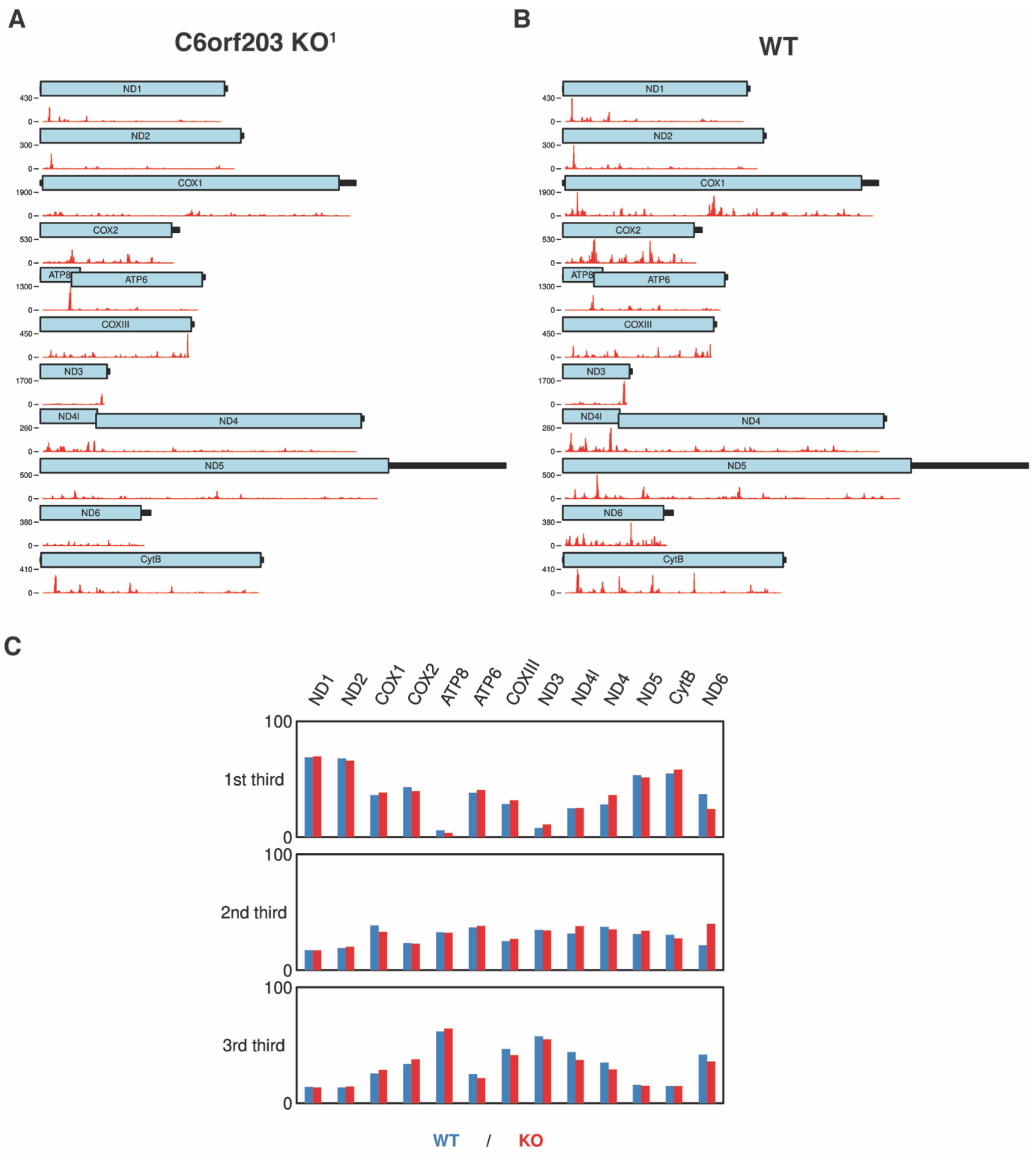

Supplementary Figure S4. Mitoribosome profiling. (A) Histograms show the positions of the 5' ends of mitochondrial ribosome-protected fragments (mtRPFs) in the C6orf203 KO[1] library. Coverage is smoothed with a 3-nt running-mean filter, and read counts are expressed as reads per million (RPM) mapped to nuclear-encoded mRNA for each sample individually. **(B)** As in Figure S3 (A), but for the WT HEK293 library. **(C)** Distribution of RiboSeq density along coding regions of transcripts for WT HEK293 (blue bars) and C6orf203 KO[1] (red bars). The "initiation" segment of each ORF, first 15 nt at the 5′ end, was excluded from the analysis and the remainder of each ORF was divided into three evenly sized segments in the 5′ to 3′ direction. The percentage of the total RPM per gene mapping within each of these segments is shown.

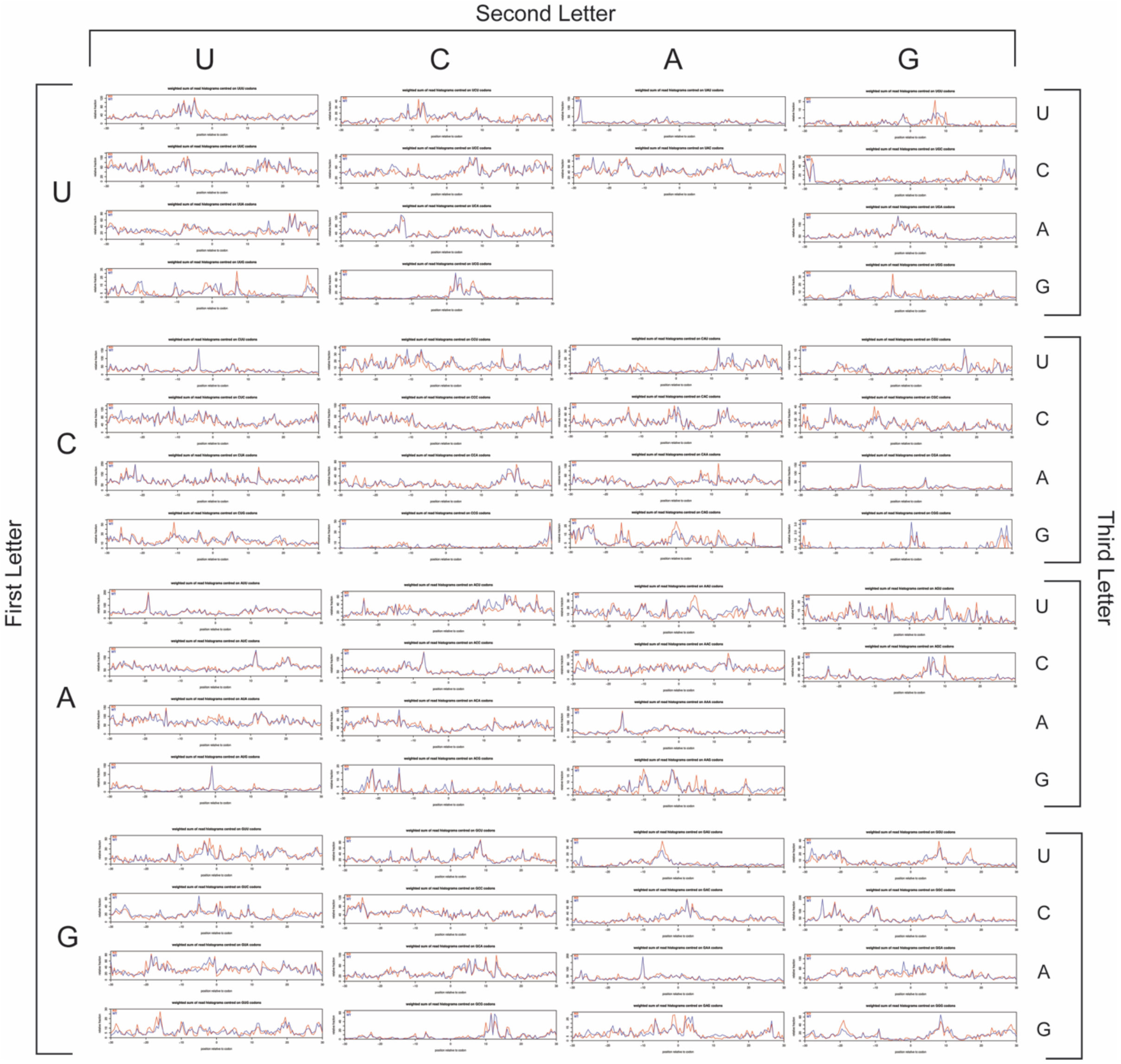

Supplementary Figure S5. Mitoribosome occupancy of individual codons in WT and C6orf203 KO as determined via MitoRiboSeq. Weighted sum of read histograms centred on each codon of the mitochondrial genetic code across all mt-ORFs. Details for determining ribosome occupancy can be found in ‘Computational analysis of MitoRibo-Seq data’ in the ‘Supplementary methods and materials’ section.

A

Organism	Class / Phylum	Gene name	% identity to *Homo sapiens*
Homo sapiens **(Human)**	Mammalia / Chordata	*C6orf203*	100%
Macaca mulatta **(Rhesus macaque)**	Mammalia / Chordata	*C4H6orf203*	95%
Canis lupus familiaris **(Dog)**	Mammalia / Chordata	*C12H6orf203*	80%
Mus musculus **(Mouse)**	Mammalia / Chordata	*N/A*	76%
Gallus gallus **(Chicken)**	Aves / Chordata	*C3H6ORF203*	70%
Boiga irregularis **(Brown tree snake)**	Reptilia / Chordata	*N/A*	66%
Xenopus laevis **(African clawed frog)**	Amphibia / Chordata	*XELAEV_18027215mg*	61%
Danio rerio **(Zebrafish)**	Actinopterygii / Chordata	*si:ch211-206k20.5*	51%
Stichopus japonicus **(Sea cucumber)**	Holothuroidea / Echinodermata	*BSL78_06441*	51%
Drosophila melanogaster **(Fruit fly)**	Insecta / Arthropoda	*Dmel\CG4884*	41%
Caenorhabditis elegans	Chromadorea / Nematoda	*C47B2.9*	39%

B

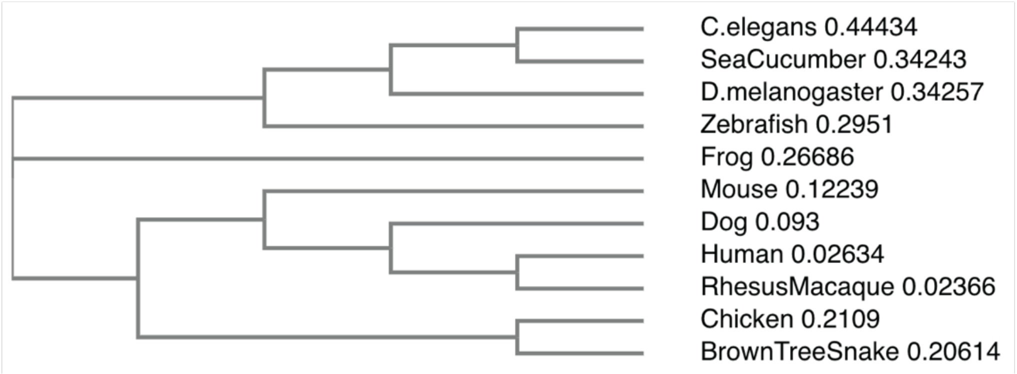

Supplementary Figure S6. Conservation of C6orf203 across animal kingdom. (A) Primary sequence of C6orf203 aligned by ClustalW with the homologous sequences, showing its conservation across the different phyla. **(B)** Phylogenetic tree generated from ClustalW showing C6orf203 of species from lower and higher phyla emerging from their respective common ancestors. Numbers indicate the distance from the common ancestors, as a measure of differences in their character sequence in contrast to human C6orf203.

SUPLEMENTARY MATERIALS AND METHODS

Liquid chromatography (LC)–mass spectrometry (MS) / MS analysis

Samples were diluted 1:10 with 20 mM Tris pH 8.3 and digested overnight at 37°C with at least 300 ng or 1:200 (protein:trypsin) of trypsin Gold (Promega). Peptides were desalted using home-made StageTips (Empore Octadecyl C18, 3M; (1) and eluted with 80 to 100 µl of 60 % acetonitrile / 0.1% formic acid buffer. The peptides were dried with a vacuum concentrator plus (Eppendorf) and resuspended with 0.1 % formic acid for mass spectrometry. Peptides were separated on a 25 cm, 75µm internal diameter PicoFrit analytical column (New Objective) packed with 1.9 µm ReproSil-Pur 120 C18-AQ media (Dr. Maisch) using an EASY-nLC 1200 (Thermo Fisher Scientific). The column was maintained at 50°C. Buffer A and B were 0.1% formic acid in water and 0.1% formic acid in 80% acetonitrile respectively. Peptides were separated on a segmented gradient from 6% to 31% buffer B for 45 mins and from 31% to 50% buffer B for 5mins at 200 nl/min. Eluting peptides were analyzed on QExactive HF mass spectrometer (Thermo Fisher Scientific). Peptide precursor m/z measurements were carried out at 60000 resolution in the 300 to 1800m/z range. The ten most intense precursors with charge state from 2 to 7 only were selected for HCD fragmentation using 25% normalized collision energy. The m/z values of the peptide fragments were measured at a resolution of 30000 using a minimum AGC target of 2e5 and 80 ms maximum injection time. Upon fragmentation, precursors were put on a dynamic exclusion list for 45sec.

The raw data were analyzed with MaxQuant version 1.6.1.0 (2) using the integrated Andromeda search engine (3). Peptide fragmentation spectra were searched against the canonical sequences of the human reference proteome (proteome ID UP000005640, downloaded September 2018 from UniProt). Methionine oxidation and protein N-terminal acetylation were set as variable modifications; cysteine carbamidomethylation was set as fixed modification. The digestion parameters were set to “specific” and “Trypsin/P,” The minimum number of peptides and razor peptides for protein identification was 1; the minimum number of unique peptides was 0. Protein identification was performed at a peptide spectrum matches and protein false discovery rate of 0.01. The “second peptide” option was on. Successful identifications were transferred between the different raw files using the “Match between runs” option. Label-free quantification (LFQ) (4) was performed using an LFQ minimum ratio count of two. LFQ intensities were filtered for at least two valid values in at least one group and imputed from a normal distribution with a width of 0.3 and down shift of 1.8. Protein quantification was performed using limma (5). Mitocarta annotations (6) were added using the primary gene name and the first of the gene name synonyms of the oldest Uniprot ID with the highest number of peptides.

Computational analysis of MitoRibo-Seq data

Adaptor sequences were trimmed using the FASTX-Toolkit, trimmed reads mapping to nuclear-encoded rRNA were discarded, and the remaining reads were mapped sequentially to mitochondrial rRNA (mt-rRNA); mitochondrial tRNA (mt-tRNA); and mitochondrial mRNA (mt-mRNA) using bowtie version 1 (7), with parameters -v 2 --best (i.e. maximum two mismatches, report best match).

When calculating the expression levels of mitochondrial genes, to normalise for different library sizes, counts for a given library were expressed as reads per million (RPM) mapped in the positive-sense orientation to nuclear-encoded mRNA in that library. Regions of CDS that overlap (ATP8/ATP6 and ND4L/ND4) were excluded due to ambiguous CDS assignment, and mitochondrial ribosome-protected fragments (mtRPFs) with 5' ends mapping within 15 nt of the start codon or 45 nt of the stop codon were excluded.

To assess ribosome occupancy of individual codons, mitoribosome-protected fragments (mtRPFs) with midpoints mapping internally to the coding regions of genes (more than 10 codons from the start and stop codons) were counted. For each codon, histograms of the counts of mtRPF midpoints in a 30 nt window centred on every instance of that codon were calculated. These values were scaled by the quotient of the total length of the coding region for a given gene and the total number of reads mapping within that coding region, to give the relative fraction of RPFs at each position in the window.

Proteinase K treatment

Isolated mitochondria were resuspended in 1 ml of 1x MSE buffer, incubated with 2U of DNase I (Invitrogen, Turbo DNase free kit) per 5 mg of mitochondria for 10 mins at 4°C, and then with 20 ug of Proteinase K (Invitrogen) per 5 mg of mitochondria for 30 mins at 4°C. After incubations, the mitochondria were washed thrice with 1mM phenylmethylsulfonyl fluoride (PMSF) (resuspended in 1x MSE buffer) to inactivate and wash away Proteinase K.

SUPPLEMENTARY TABLES

Supplementary Table 1. List of Antibodies

**Antibody**	**Company**	**Catalog no.**
Anti-FLAG	Abcam	ab1257
C6orf203	Prestige Antibodies, Sigma-Aldrich	HPA049535-100UL
GAPDH	Abcam	Ab8245
β-tubulin	Sigma-Aldrich	T6199
MRPS16	Prestige Antibodies, Sigma-Aldrich	HPA054538
MRPS35	Proteintech Group	16457-1
MRPS15	Proteintech Group	17006-1
MRPS22	Proteintech Group	10984-1
MRPS17	Proteintech Group	18881-1
MRPL49	Proteintech Group	15542-1
MRPL3	Prestige Antibodies, Sigma-Aldrich	HPA043665
MRPL37	Prestige Antibodies, Sigma-Aldrich	HPA025826
MRPL12	Prestige Antibodies, Sigma-Aldrich	HPA022853
uL1	Santa-Cruz	sc-100827
VDAC1/Porin	Abcam	128568
MT-CO2	Abcam	ab110258
SDHA	Abcam	ab14715
ATP5a	Abcam	ab14748
MT-CO1	Abcam	ab14705
NDUFB8	Abcam	ab110242
HRP secondary rabbit	GE Healthcare	NA9340V
HRP secondary mouse	GE Healthcare	NA9310V
HRP secondary goat	Santa-Cruz	sc-2354

Supplementary Table 2. List of guide RNAs

**guideRNAs for C6orf203 exon 1**	**Sequence (5'→ 3')**
gRNA_set1_Top	CACCGATGATGTTGTCCTGAAGA
gRNA_set1_Bottom	AAACCGTCTTCAGGACAACATCA
gRNA_set2_Top	CACCGTTTTCTCACTGAGACTCC
gRNA_set2_Bottom	AAACTGGGAGTCTCAGTGAGAAA
gRNA_set3_Top	CACCGTGTATGATGAAGGTGTCC
gRNA_set3_Bottom	AAACAGGGACACCTTCATCATAC

Screen deletion_C6_Fwd	TGCACTTGTGAGATAAAACTG
Screen deletion_C6_Rev	CAGGTTACCTACAAAGGTCC

Supplementary Table 3. List of primers and oligonucleotides for tRNA probe preparation

Primer/Oligo	Sequence (5'→ 3')
H_T7_Met_68_Fwd	TAATACGACTCACTATAGGGAGACtagtacgggaagggtataacc
H_Met_68_Rev	agtaaggtcagctaaataagctatc
H_T7_Val_69_Fwd	TAATACGACTCACTATAGGGAGACtcagagcggtcaagttaagttg
H_Val_69_Rev	cagagtgtagcttaacacaaagc
L_T7_Gln_72_Fwd	TAATACGACTCACTATAGGGAGACctaggactatgagaatcgaac
L_Gln_72_Rev	taggatggggtgtgatagg
L_T7_Tyr_66_Fwd	TAATACGACTCACTATAGGGAGACtggtaaaaagaggcctaaccc
L_Tyr_66_Rev	ggtaaaatggctgagtgaagc
L_T7_Pro_68_Fwd	TAATACGACTCACTATAGGGAGACtcagagaaaaagtctttaactc
L_Pro_68_Rev	cagagaatagtttaaattagaatc
L_T7_Glu_69_Fwd	TAATACGACTCACTATAGGGAGACtattctcgcacggactacaac
L_Glu_69_Rev	gttcttgtagttgaaatacaacg
L_T7SerUCN_69_Fwd	TAATACGACTCACTATAGGGAGACcaaaaaaggaaggaatcgaacc
L_SerUCN_69_Rev	gaaaaagtcatggaggccatg
L_T7_Cys_66_Fwd	TAATACGACTCACTATAGGGAGACaagccccggcaggtttgaag
L_Cys_66_Rev	agctccgaggtgattttcatattg
L_T7_Asn_73_Fwd	TAATACGACTCACTATAGGGAGACctagaccaatgggacttaaac
L_Asn_73_Rev	tagattgaagccagttgattag
L_T7_Ala_69_Fwd	TAATACGACTCACTATAGGGAGACtaaggactgcaaaaccccac
L_Ala_69_Rev	aagggcttagcttaattaaagtgg
H_T7_Phe_71_Fwd	TAATACGACTCACTATAGGGAGACtgtttatggggtgatgtgag
H_Phe_71_Rev	gtttatgtagcttacctcctc
H_T7LeuUUR_75_Fwd	TAATACGACTCACTATAGGGAGACtgttaagaagaggaattgaacctctg
H_LeuUUR_75_Rev	gttaagatggcagagcccgg
H_T7_Ile_69_Fwd	TAATACGACTCACTATAGGGAGACtagaaataagggggtttaagctc
H_Ile_69_Rev	agaaatatgtctgataaaagagttac
H_T7_Trp_68_Fwd	TAATACGACTCACTATAGGGAGACcagaaattaagtattgcaacttac
H_Trp_68_Rev	agaaatttaggttaaatacagacc
H_T7_Asp_68_Fwd	TAATACGACTCACTATAGGGAGACtaagatatataggatttagcctata
H_Asp_68_Rev	aaggtattagaaaaaccatttcat
H_T7_Lys_70_Fwd	TAATACGACTCACTATAGGGAGACtcactgtaaagaggtgttgg
H_Lys_70_Rev	cactgtaaagctaacttagcat

H_T7_Gly_68_Fwd	TAATACGACTCACTATAGGGAGACtactcttttttgaatgttgtcaaaac
H_Gly_68_Rev	actcttttagtataaatagtaccg
H_T7_Arg_65_Fwd	TAATACGACTCACTATAGGGAGACttggtaaatatgattatcataatttaatg
H_Arg_65_Rev	tggtatatagtttaaacaaaacgaa
H_T7_His_69_Fwd	TAATACGACTCACTATAGGGAGACggtaaataaggggtcgtaagc
H_His_69_Rev	gtaaatatagtttaaccaaaacatcag
H_T7SerAGY_59_Fwd	TAATACGACTCACTATAGGGAGACtgagaaagccatgttgttagac
H_SerAGY_59_Rev	gagaaagctcacaagaactg
H_T7LeuCUN_71_Fwd	TAATACGACTCACTATAGGGAGACtacttttatttggagttgcacc
H_LeuCUN_71_Rev	acttttaaaggataacagctatcc
H_T7_Thr_66_Fwd	TAATACGACTCACTATAGGGAGACtgtccttggaaaaaggttttcatc
H_Thr_66_Rev	gtccttgtagtataaactaatacac

Supplementary Table 4. List of primers and oligonucleotides for mRNA probe preparation.

**Primer**	**Sequence (5'→ 3')**
12S Fwd	CACTGAAAATGTTTAGACGGG
12S Rev	GGCTCCTCTAGAGGGATATG
16S Fwd	TAGATATAGTACCGCAAGGG
16S Rev	GACTTGTTGGTTGATTGTAG
ND1 Fwd	AACCTCAACCTAGGCCTCC
ND1 Rev	AATGCTAGGGTGAGTGGTAGG
ND2 Fwd	TCCCAGAGGTTACCCAAG
ND2 Rev	GAGTAGTGTGATTGAGGTGGAG
ND4L/ND4 Fwd	ACTACCACTGACATGACTTTCC
ND4L/ND4 Rev	GGAGTCATAAGTGGAGTCCG
ND5 Fwd	GTAGCATTGTTCGTTACATGG
ND5 Rev	ACTGCTGCGAACAGAGTG
CO1 Fwd	CTTATTCGAGCCGAGCTG
CO1 Rev	GGTATAGAATGGGGTCTCCTC
CO2 Fwd	GCGCAAGTAGGTCTACAAGACGC
CO2 Rev	GCATGAAACTGTGGTTTGCTCC
ATP8/6 Fwd	CCCATACTCCTTACACTATTCC
ATP8/6 Rev	GTTAGCGGTTAGGCGTAC
CYTB Fwd	CTACCTTCACGCCAATGG
CYTB Rev	TTTGTTAGGGACGGATCG
CO3 Fwd	CCTGAGAACCAAAATGAACG
CO3 Rev	GCCAGGGCTATTGGTTGAATG

ND3 Fwd	ATAAACTTCGCCTTAATTTTAATAATC
ND3 Rev	TAATACGACTCACTATAGGGATTCGGTTCAGTCTAATCCTTTTTGTAG
ND6 Fwd	GGGGTTTTCTTCTAAGCCTTC
ND6 Rev	TAATACGACTCACTATAGGGCCCCCGAGCAATCTCAATTAC

Supplementary Table 5. List of TaqMan probes used for qRT-PCR

TaqMan probe	Assay ID
MT-ND1	Hs02596873
MT-ND2	Hs02596874
MT-ND3	Hs02596875
MT-ND4	Hs02596876
MT-ND5	Hs02596878
MT-ND6	Hs02596879
MT-CYTB	Hs02596867
MT-COX1	Hs02596864
MT-COX2	Hs02596865
MT-COX3	Hs02596866
MT-ATP6	Hs02596862
MT-ATP8	Hs02596863
MT-RNR1	Hs02596859
MT-RNR2	Hs02596860

SUPPLEMENTARY REFERENCES

1. Rappsilber,J., Ishihama,Y. and Mann,M. (2003) Stop And Go Extraction tips for matrix-assisted laser desorption/ionization, nanoelectrospray, and LC/MS sample pretreatment in proteomics. *Anal. Chem.*, **75**, 663–670.
2. Cox,J. and Mann,M. (2008) MaxQuant enables high peptide identification rates, individualized p.p.b.-range mass accuracies and proteome-wide protein quantification. *Nat. Biotechnol.*, **26**, 1367–1372.
3. Cox,J., Neuhauser,N., Michalski,A., Scheltema,R.A., Olsen,J. V. and Mann,M. (2011) Andromeda: A peptide search engine integrated into the MaxQuant environment. *J. Proteome Res.*, **10**, 1794–1805.
4. Hein,M.Y., Luber,C.A., Paron,I., Cox,J., Nagaraj,N. and Mann,M. (2014) Accurate Proteome-wide Label-free Quantification by Delayed Normalization and Maximal Peptide Ratio Extraction, Termed MaxLFQ. *Mol. Cell. Proteomics*, **13**, 2513–2526.
5. Ritchie,M.E., Phipson,B., Wu,D., Hu,Y., Law,C.W., Shi,W. and Smyth,G.K. (2015) Limma powers differential expression analyses for RNA-sequencing and microarray studies. *Nucleic Acids Res.*, **43**, e47.
6. Calvo,S.E., Clauser,K.R. and Mootha,V.K. (2016) MitoCarta2.0: An updated inventory of mammalian mitochondrial proteins. *Nucleic Acids Res.*, **44**, D1251–D1257.
7. Langmead,B., Trapnell,C., Pop,M. and Salzberg,S.L. (2009) Ultrafast and memory-efficient alignment of short DNA sequences to the human genome. *Genome Biol.*, **10**.
8. Ban,N., Beckmann,R., Cate,J.H.D., Dinman,J.D., Dragon,F., Ellis,S.R., Lafontaine,D.L.J., Lindahl,L., Liljas,A., Lipton,J.M., *et al.* (2014) A new system for naming ribosomal proteins. *Curr. Opin. Struct. Biol.*, **24**, 165–169.
